# Plant-Based Phytochemical Screening by Targeting Main Protease of SARS-CoV-2 to Design Effective Potent Inhibitors

**DOI:** 10.3390/biology10070589

**Published:** 2021-06-26

**Authors:** Shafi Mahmud, Suvro Biswas, Gobindo Kumar Paul, Mohasana Akter Mita, Maria Meha Promi, Shamima Afrose, Md. Robiul Hasan, Shahriar Zaman, Md. Salah Uddin, Kuldeep Dhama, Talha Bin Emran, Md. Abu Saleh, Jesus Simal-Gandara

**Affiliations:** 1 Microbiology Laboratory, Department of Genetic Engineering and Biotechnology, University of Rajshahi, Rajshahi 6205, Bangladesh; shafimahmudfz@gmail.com (S.M.); gobindokumar38@gmail.com (G.K.P.); szaman@ru.ac.bd (S.Z.); salim.geb@ru.ac.bd (M.S.U.); 2 Department of Genetic Engineering and Biotechnology, University of Rajshahi, Rajshahi 6205, Bangladesh; suvrobiswas0@gmail.com (S.B.); mohasanamita26@gmail.com (M.A.M.); meha.mitsa@gmail.com (M.M.P.); shamimaafrose.ru@gmail.com (S.A.); rhonanik7@gmail.com (M.R.H.); 3 Division of Pathology, Indian Veterinary Research Institute, Izatnagar, Bareilly 243122, UP, India; kdhama@rediffmail.com; 4 Department of Pharmacy, BGC Trust University Bangladesh, Chittagong 4381, Bangladesh; 5 Nutrition and Bromatology Group, Department of Analytical and Food Chemistry, Faculty of Food Science and Technology, University of Vigo–Ourense Campus, E32004 Ourense, Spain

**Keywords:** SARS-CoV-2, phytochemicals, molecular docking, ADMET, dynamics simulation

## Abstract

**Simple Summary:**

Virtual screening schemes, including molecular docking in conjunction with molecular dynamics simulation, were accomplished, as they extend an ample opportunity to screen plausible inhibitors of the main protease from an extensive phytochemical library. The preferential phytochemicals were retrieved from Asian plants through the data mining procedure and comprehensive literature study. The three preeminent reliable phytochemical exhibited toxicity by no means during the scrutinization of ADMET prominences. Moreover, pharmacologically distinguishing characteristics and the biological activity of the lead phytochemicals were satisfying as a repurposing antiviral drug contender. Additionally, the molecular dynamics simulation exhibited thermal stability and a stable binding affinity of the protein-compound complex that refers to the appreciable efficacy of the lead optimization. Therefore, the preferable phytochemicals are worth substantial evaluation in the biological laboratory to recommend plausible antiviral drug contenders.

**Abstract:**

Currently, a worldwide pandemic has been declared in response to the spread of coronavirus disease 2019 (COVID-19), a fatal and fast-spreading viral infection caused by severe acute respiratory syndrome coronavirus 2 (SARS-CoV-2). The low availability of efficient vaccines and treatment options has resulted in a high mortality rate, bringing the world economy to its knees. Thus, mechanistic investigations of drugs capable of counteracting this disease are in high demand. The main protease (M^pro^) expressed by SARS-CoV-2 has been targeted for the development of potential drug candidates due to the crucial role played by M^pro^ in viral replication and transcription. We generated a phytochemical library containing 1672 phytochemicals derived from 56 plants, which have been reported as having antiviral, antibacterial, and antifungal activity. A molecular docking program was used to screen the top three candidate compounds: epicatechin-3-O-gallate, psi-taraxasterol, and catechin gallate, which had respective binding affinities of −8.4, −8.5, and −8.8 kcal/mol. Several active sites in the targeted protein, including Cys145, His41, Met49, Glu66, and Met165, were found to interact with the top three candidate compounds. The multiple simulation profile, root-mean-square deviation, root-mean-square fluctuation, radius of gyration, and solvent-accessible surface area values supported the inflexible nature of the docked protein–compound complexes. The toxicity and carcinogenicity profiles were assessed, which showed that epicatechin-3-O-gallate, psi-taraxasterol, and catechin gallate had favorable pharmacological properties with no adverse effects. These findings suggest that these compounds could be developed as part of an effective drug development pathway to treat COVID-19.

## 1. Introduction

In December 2019, approximately 41 patients were diagnosed with anomalous pneumonia of unfamiliar etiology in Wuhan City, China, and, within a relatively short time, this disease spread rapidly worldwide [1,2,3]. The etiology of this pneumonia, which has since been named coronavirus disease 2019 (COVID-19), was eventually identified as a novel coronavirus, which was formally designated as severe acute respiratory syndrome coronavirus 2 (SARS-CoV-2) [1]. This virus has wreaked havoc worldwide due to the scarcity of health frameworks and a lack of preparation for a disease of this scale, causing 2,470,772 deaths and infecting 111,419,939 people thus far [4].

The SARS-CoV-2 is single-stranded, positive-sense RNA (+ssRNA) viruses that belong to the *Betacaronavirus* (β-CoV) genus with a genome size that ranges from 26 to 32 kb [5]. Coronaviruses can be classified into four genera: alpha-CoV, beta-CoV, gamma-CoV, and delta-CoV. Among these, only the alpha-CoV and beta-CoV genera have been shown to infect humans [6,7]. Coughing, sneezing, respiratory droplets, and fomites represent the primary vectors for viral spread [8,9].

The SARS-CoV-2 genome consists of a 5ʹ methyl-guanosine cap structure, a 5ʹ-untranslated region (UTR), open reading frame (ORF), a 3ʹ-UTR, and a poly-adenosine (poly-A) tail [10,11,12]. ORF 1ab encodes 16 nonstructural proteins (nsp 1 to nsp 16), which are indispensable for viral replication. The remainder of the genome encodes four structural proteins, membrane protein (M), spike glycoprotein (S), envelope protein (E), and nucleocapsid protein (N), in addition to other accessory proteins, including ORFs 3a, 7a/b, 6, and 8 [13,14,15,16].

SARS-CoV-2 invades alveolar type II cells following the interaction between the S protein and the angiotensin-converting enzyme 2 (ACE-2) receptor, causing acute alveolar damage [4]. Afterward, the viral genome attaches to the host’s ribosomes, resulting in the translation of large polyproteins that are later modified by proteolysis [17,18]. The SARS-CoV-2 genome shares approximately 96% and 80% sequence identity with the bat coronavirus (BatCoV) RaTG13 and SARS-CoV, respectively [14,19,20]. Pangolin-CoV has also been found to share 91.02% sequence identity with SARS-CoV-2 [21].

A cysteine protease, known as main protease (M^pro^), plays a central role in the post-translational modification of replicase polyproteins [14,22,23]. ORF 1ab encodes the polyproteins pp1a and pp1ab, which are cleaved by M^pro^ into abundant functional units that are responsible for viral replication and transcription [14]. M^pro^ exhibits unique enzymatic activity and is involved in the processing of all viral polyproteins [22,24]. Following the translation of viral mRNA into polyproteins, Mpro exerts an autocleavage function that results in the mature enzyme, which then processes the polyprotein into 11 nsps that regulate the viral replication process [25,26]. Therefore, viral polyprotein processing, viral replication, viral transcription, and viral maturation are all dependent on M^pro^ activity [9,27,28,29,30], and the inhibition of M^pro^ should prevent viral replication and spread [27]. A specific M^pro^ inhibitor would likely be nontoxic because no human proteases share any corresponding recognition sequences with M^pro^ [27,31]. For these reasons, SARS-CoV-2 M^pro^ represents a promising target for antiviral drug discovery [25,27,32].

The currently available antiviral agents approved for clinical use have demonstrated limited efficacy and are associated with adverse reactions, including enhanced viral resistance following long-term therapy. By contrast, antiviral therapeutics that have been developed based on phytochemicals have been reported to have more tolerable side effects and can serve as a reliable alternative to synthetic antiviral drugs for the inhibition of viral replication and penetration [33,34,35,36]. In humans, 4’,5-dihydroxy-3,7,3′-trimethoxyflavone demonstrated antiviral efficacy against rhinoviruses, coxsackieviruses, and picornaviruses, whereas gingerol was shown to act against the common cold virus. Myricetin can act as an antiviral phytochemical against the human immunodeficiency virus, Rhesus lymphocryptovirus, and influenza virus. Additionally, apigenin exhibits antiviral activity against Enterovirus 71, hepatitis C virus, and African swine fever virus; quercetin inhibits the Epstein–Barr virus, influenza A, and rhinovirus; and curcumin has been characterized as an antiviral agent for Zika virus, chikungunya virus, herpes simplex virus, human papillomavirus, and cytomegalovirus [33,37]. Therefore, phytochemicals represent a plausible class of bioactive antiviral compounds that can be developed into therapeutics against various strains of SARS-CoV-2 [34].

An alkaloid named ‘Quinine’ is applied for *Plasmodium falciparum* malaria [38], whereas another phytochemical named ‘Podophyllin’ is utilized as pharmaceutics for treating perianal warts and external genital warts [39]. Additionally, another phytochemical-based hepatoprotective drug named ‘Glycyrrhizic acid’ exhibits curative effects against chronic hepatitis [40]. Notably, quinine, podophyllin, and glycyrrhizic acid have already been approved by FDA (Food and Drug Administration) for therapeutics use. Furthermore, FDA-approved antiviral drugs, including letermovir, baloxavir, marboxil, nelfinavir, and ombitasvir, are applied to treat cytomegalovirus infections, influenza, HIV infection, and hepatitis C virus (HCV) infections, respectively [41,42,43,44].

The computer-aided screening process can be a valid source to develop effective therapeutic solution against target protein. Here, a deep literature mining was conducted to enlist effective phytochemicals candidates which finally screened against the target main protease of SARS-CoV-2. The hit molecules obtained from molecular docking were finally assessed in molecular dynamics simulation due to low efficacy in binding mode, motion effect, and solvation effect in molecular docking pipelines. These compound can be further utilized in in vitro and in vivo experiments against antiviral protein of SARS-CoV-2.

## 2. Materials and Methods

### 2.1. Protein Preparation

The three-dimensional (3D) crystal structure (PDB ID: 6LU7; Method: X-ray diffraction; Resolution: 2.16 Å; Organism: SARS-CoV-2) of the SARS-CoV-2 main protease (M^pro^) was retrieved from the Protein Data Bank (PDB) database maintained by Research Collaboratory for Structural Bioinformatics (RCSB) [45]. Water molecules and every heteroatom in the protein structure were removed using Pymol [46] and Discovery Studio software [47]. The cleaned crystal structure was then optimized, verified, and energy-minimized using the GROMOS 43B1 force field via the Swiss-PDB viewer [48].

### 2.2. Ligand Preparation

A total of 1672 compounds were included in the screening (Tables S1–S3), encoded by various plants, after a profound literature review. The PubMed, Google Scholar, PubChem, Phytochemical database, Dr. Duke database, IMPPAT, and Google search were employed to enlist the phytochemicals presents in the plant compounds. Furthermore, these data sets were also validated by examining the gas chromatography-mass spectroscopy and liquid chromatography-mass spectroscopy data from the literature. The duplicates were removed to maintain the accuracy of the data sets. The 3D structures of these molecules were extracted from the PubChem database [49]. The ligand structures were optimized, cleaned, prepared, and minimized using the mmff94 force field [50], using the optimization algorithm with the steepest descent and a total number of 2000 minimization steps.

### 2.3. Molecular Docking Study

Molecular docking was performed via the PyRx Virtual Screening Tool [51] to explore all possible orientations, conformations, and binding affinities for the ligand with the potential M^pro^ binding sites. All ligand free energy values were minimized using the universal force field (UFF), combined with the conjugate gradient algorithm and 2000 minimization steps. All ligands were converted to PDBQT format to prepare them in an acceptable format for docking in AutoDock Vina. The docking was configured as protein-fixed and ligand-flexible, allowing for all ligand bonds to rotate. The center points of the grid box were set to X: −26.299, Y: 12.6039, and Z: 58.9455, and the dimensions (in Å) were X: 50.3334, Y: 67.2744, and Z: 59.2586. PyMol and BIOVIA Discovery Studio were used to visualize the non-bonding interactions between the docked protein–ligand complexes were further applied to analyze the docking pose. The best conformation was selected based on the lowest docking score (in kcal/mol).

### 2.4. ADMET

ADMET (absorption, distribution, metabolism, excretion, and toxicity) predictions were used, using reliable online servers including SwissADME [52], admetSAR [53], and pKCSM [54] to examine the pharmacokinetic properties. Canonical simplified molecular-input line-entry system (SMILES) structures for the screened phytochemicals were retrieved from the PubChem database and used as inputs for the webservers to generate predictions regarding their drug-likeness properties.

### 2.5. Molecular Dynamics Simulation

Molecular dynamics simulations for the docked complexes were performed to understand the structural variations across the simulation trajectories. The YASARA dynamics software [55] package was used to run the molecular dynamics simulations, in which the AMBER14 force field [56] was employed. A cubic simulation cell was created, and our ligand–protein complexes were incorporated into the simulation’s cells. The simulation cell was extended 20 Å more than the protein complex, to move freely. The TIP3P water solvation model was used, and the steepest gradient approaches were used to perform the energy minimization procedure using simulated annealing methods [57]. The simulation environment was neutralized at a temperature of 310 K, pH 7.4, and 0.9% NaCl. The long-range electrostatic interactions were calculated using Particle Mesh Ewald’s algorithms by setting a cutoff radius of 8 Å [58]. The simulation time was set to 1.25 fs. Each simulation was run for 100 ns, and each trajectory was saved after 100 ps. Finally, simulation snapshots were used to calculate the root-mean-square deviation (RMSD), root-mean-square fluctuation (RMSF), the radius of gyration (Rg), solvent-accessible surface area (SASA), and hydrogen bonds [59,60,61,62,63,64,65,66,67,68].

## 3. Results

### 3.1. Molecular Docking Analysis

**Figure 1:**
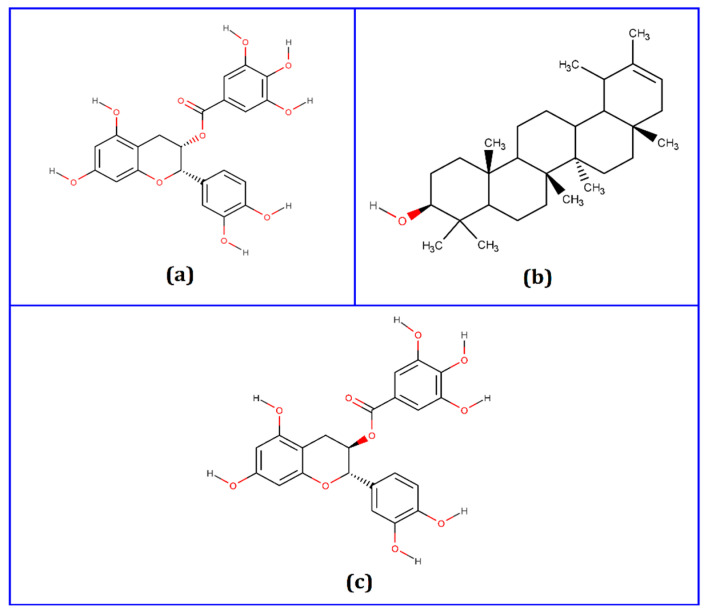
Two-dimensional (2D) chemical structures of (**a**) epicatechin-3-O-gallate, (**b**) psi-taraxasterol, and (**c**) catechin gallate. The structures were drawn using MarvinSketch software.

Based on the most significant negative docking scores, we set the thresholds of −7.5 kcal/mol [69]. The top three potential candidates (Figure 1) were selected for further analysis, including epicatechin-3-O-gallate, psi-taraxasterol, and catechin gallate, which had binding affinities of −8.4, −8.5, and −8.8 kcal/mol, respectively.

The epicatechin-3-O-gallate–M^pro^ complex formed three conventional hydrogen bonds at His164, His163, and Asn142, one carbon–hydrogen bond at Gln189, and two pi-alkyl bonds at Met165 and Pro168. In contrast, the complex between M^pro^ and psi-taraxasterol was stabilized by three alkyl bonds at Met165, Met49, and Cys145, and one pi-alkyl bond at His41 position.

**Figure 2:**
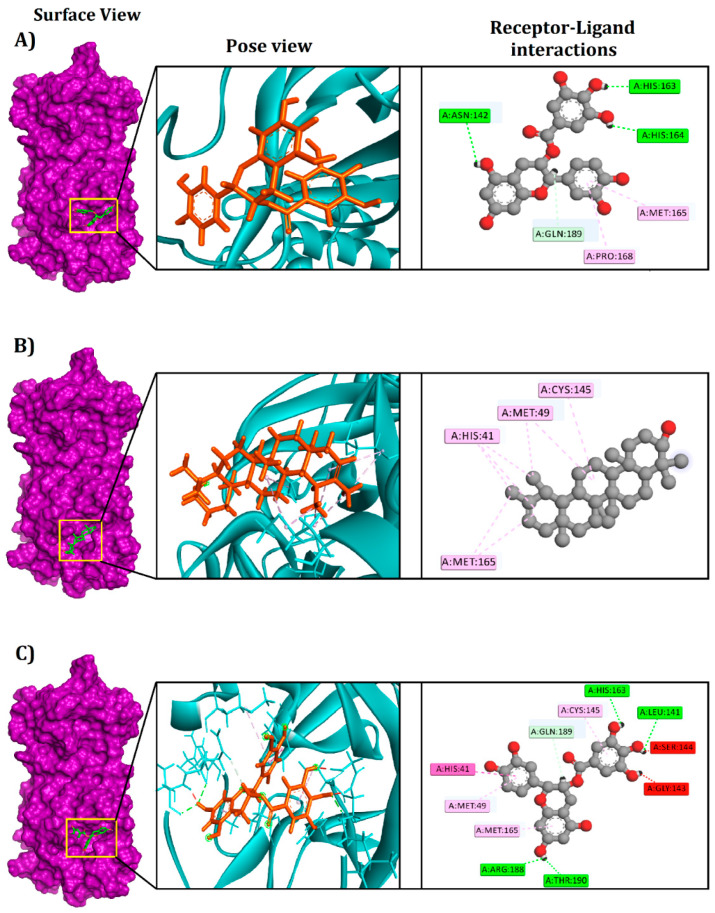
The various binding modes for the selected compounds within the active and catalytic sites of the SARS-CoV-2 main protease. (**A**) epicatechin-3-O-gallate, (**B**) psi-taraxasterol, and (**C**) catechin gallate.

Catechin gallate interacted with M^pro^ through four conventional hydrogen bonds at Leu141, His163, Arg188, and Thr190, one carbon–hydrogen bond at Gln189, one pi-pi T-shaped bond at His4, and three pi-alkyl bonds at Met165, Cys145, and Met49 position (Figure 2 and Table 1).

### 3.2. ADMET

To examine each candidate molecule’s efficiency and safety characteristics, various properties of the ligands, including toxicity and pharmacokinetics profiles, were evaluated. Additional parameters of the lead molecules, including p-glycoprotein inhibition, central nervous system (CNS) permeability, carcinogenicity, hepatotoxicity, human intestinal absorption, and cytochrome P (CYP) inhibition, were computationally predicted (Table 2). CNS permeability refers to the ability of a molecule to permeate across the selective, semipermeable border of the blood–brain barrier. CNS permeability greater than −2 (CNS > −2) is considered to indicate the capacity to penetrate the CNS. The psi-taraxasterol had the CNS permeability of about −2.0 (1.992). The lower level of efficacy and toxicity is the main driving force for the failure of the drug candidates in clinical trials. The prime determinant of the drug penetrability of the biological systems depends on how efficiently they are permeable in the central nervous system and cell membranes [70]. The top three compounds, including the psi-taraxasterol, had a better probability of crossing the biological barriers in the ADMET study, which defines the better efficacy probability of the compounds. Among the three top candidate compounds, no probability of toxicity or carcinogenicity was observed. The molecular weights (MWs) of these three compounds were 442.4, 426.7, and 442.4 g/moL for epicatechin-3-O-gallate, psi-taraxasterol, and catechin gallate, respectively. These MWs were optimal for prime molecule consideration because compounds with higher MWs transgress the Lipinski rule of five. The numbers of hydrogen bond acceptors and donors for these three ligand molecules were also evaluated. The numbers of hydrogen bond donors were reported as 7, 1, and 7, respectively, for epicatechin-3-O-gallate, psi-taraxasterol, and catechin gallate, whereas the numbers of hydrogen bond acceptors were 10, 1, and 10. All three ligands were found to maintain the Lipinski rule of five and none were found to cause acute oral toxicity or hepatotoxicity.

### 3.3. Molecular Dynamics Simulation

**Figure 3:**
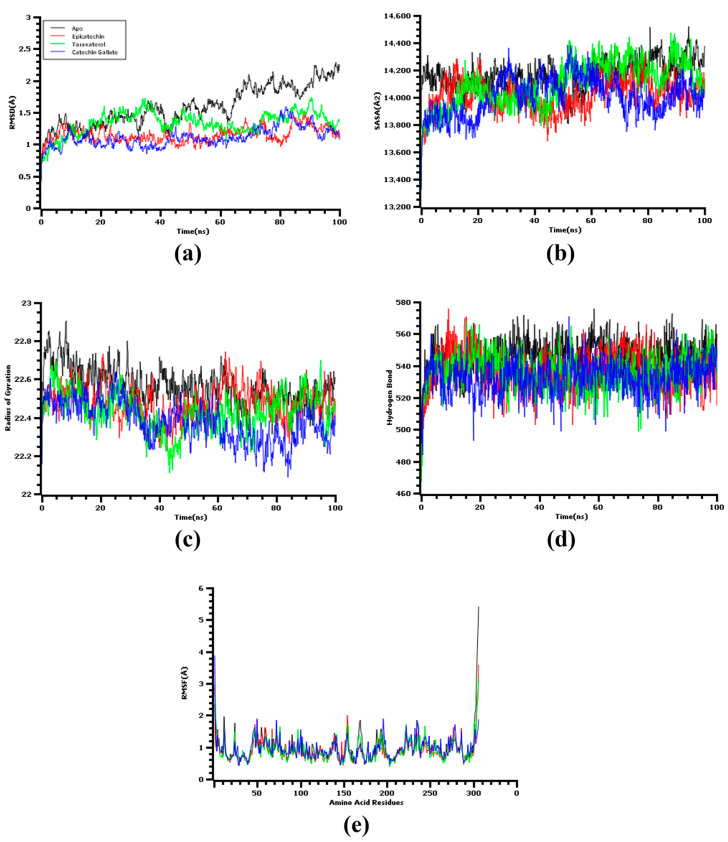
Time series analyses for all three simulated systems. (**a**) RMSD analysis for the alpha carbon atoms, (**b**) protein volume with expansion analysis, (**c**) degree of rigidity and compactness analysis, (**d**) hydrogen bond analysis, and (**e**) flexibility analysis of amino acid residues.

The RMSD of the C-alpha atoms from the simulation trajectories were assessed to understand changes in the protein–ligand complex’s stability. Figure 3a shows that the psi-taraxasterol–M^pro^ complex presented an initial increase in its RMSD profile between 0 and 40 ns due to a higher degree of instability in the protein–ligand complex. The RMSD profile decreased drastically at 40 ns before increasing again at 50 ns. The RMSD descriptors from this complex indicated the maintenance of stability after 50 ns, with few fluctuations observed. In contrast, the other two complexes, formed between M^pro^ and epicatechin or catechin gallate, both achieved stable RMSD profiles after 10 ns in the simulations. Although the psi-taraxasterol complex exhibited a higher degree of deviation compared with epicatechin-3-O-gallate and catechin gallate, psi-taraxasterol did not exceed its RMSD profile by more than 2.5 Å. The average RMSD of apo, epicatechin-3-O-gallate, psi-tarasterol, and catechin gallate were 1.591, 1.151,1.337, 1.111 Å, respectively. 

Moreover, the SASAs of the simulation complexes were analyzed to determine changes in the protein volume. A larger SASA profile indicates the expansion of the protein surface area, whereas a lower SASA profile denotes the truncation of the protein–ligand complex. The catechin gallate–M^pro^ complex displayed an expanded surface area after 20 ns in the simulation, which was stably maintained within the simulation environment. A lower degree of change in the SASA profile was observed for this compound compared with the other two compounds, but the deviations were not large. The psi-taraxasterol and epicatechin-3-O-gallate complexes had similar SASA profiles starting at the beginning of the simulations, and each compound displayed slightly expanded surface areas at 60 ns (Figure 3b). The average of the SASA from the apo, epicatechin-3-O-gallate, psi-tarasterol, and catechin gallate were 14163.56, 14013.39, 14097.55, and 14004.83 Å^2^, respectively. 

The Rg values from the simulation trajectories were examined to understand the labile nature of the drug–protein complexes. Figure 3c shows that the Rg values for the three complexes decreased from 0 to 30 ns during the simulation. After 30 ns, all three complexes showed similar, straight-line Rg profiles for the remainder of the simulation. A low Rg profile correlates with a less labile nature and the increased rigidity of the simulated complexes. The average Rg of the apo, epicatechin-3-O-gallate, psi-tarasterol, and catechin gallate were 22.567, 22.447, 22.429, and 22.378 Å, respectively. 

The formation of hydrogen bonds in the simulation system defines the integrity and stable nature of each protein–ligand complex. Figure 3d demonstrates that all three complexes formed stable hydrogen bonds throughout the 100 ns molecular dynamics simulation trajectories. The average hydrogen bond between solute and solvent systems of the apo, epicatechin-3-O-gallate, psi-tarasterol, and catechin gallate were 545.977, 536.373, 535.982, and 532.281, respectively. 

In addition, the RMSF profiles of all three compounds were analyzed to understand changes in the amino acid residues involved in the hydrogen bond formation.

Figure 3e indicates that most of the amino acid residues had low RMSF values for all three complexes, except Ser1, Leu50, Asn72, Tyr154, Thr196, Ser301, Val303, Thr304, Phe305, and Gln306.

The binding interactions of the top three docked complexes were further evaluated after 100 ns in the simulation to understand their changes after the simulation study (Table 3). Epicatechin-3-O-gallate formed had five hydrogen bonds with M^pro^, at Ser144, His163, Phe140, Cys145, and His41, and three pi-alkyl bonds were observed for Leu141, Met49, and Cys165. Psi-taraxasterol formed three hydrogen bonds with M^pro^ at Cys145, Met165, and Met49. This complex also formed a pi-alkyl bond at His41. Catechin gallate exhibited a higher number of hydrogen bonds after the 100-ns simulation, forming seven hydrogen bonds at Asn142, Leu141, Glu166, Met49, Val186, Arg188, and Met165 and two pi-alkyl bonds at Cys145 and His41.

## 4. Discussion

The SARS-CoV-2 virus causes a fatal infection of the respiratory tract, and the viral genome features 1516 nucleotide-level variations based on genome-wide annotations. Currently, no highly effective therapeutic treatments are widely available, and combating the outbreak has become a global crisis [71,72]. Computer-aided drug design can be used as an efficient method for recognizing reliable drug candidates that can be repurposed for use against SARS-CoV-2 because detailed analyses of receptor−ligand interactions and 3D structures of the main viral proteins have become available. Although no efficient inhibitor for the targeted SARS-CoV-2 protease has yet been implemented in any clinical development programs, non-covalent protease inhibitors have potential advantages for combating viral infections [30,72,73,74].

The active sites of M^pro^ are largely conserved across various coronaviruses, and M^pro^ is functionally important for viral replication and the viral life cycle; therefore, the inhibition of M^pro^ represents a feasible strategy for the generation of plausible antiviral drugs designed to suppress the ongoing SARS-CoV-2 impacts [75,76,77].

According to the 3D structure of SARS-CoV-2 M^pro^, the active M^pro^ homodimer comprises two protomers, constituting three domains. Amino acid residues 8–101 constitute Domain I, amino acid residues 102–184 constitute Domain II, and amino acid residues 201–306 constitute Domain III. The substrate-binding zone, which is found in the cleft between Domain I and Domain II, resembles the SARS-CoV-2 active site and consists of a conserved and noncanonical His41 and Cys145 catalytic dyad. His41 is a well-known conserved residue across coronaviruses [73,76].

Additionally, S1 and S2 represent densely buried subsites, with S1 comprised of Phe140, Glu166, Gly143, Cys145, Glu166, and His163 and S2 comprised of His41, Thr25, and Cys145. Moreover, the shallow S3–S5 subsites are comprised of Gln189, Met49, Glu166, Met165, Glu166, and His41 residues [30,75,76,77,78,79,80,81].

The virtual screening technique represents a feasible screening process that can be used to identify a plausible inhibitor from among an expansive array of phytochemicals. Molecular docking allows for the evaluation of interaction binding affinities between the target protein and a diverse array of ligands. Thus, the application of the molecular docking approach allows for the rapid recognition of potentially effective inhibitors through the assessment of the binding energies of a massive quantity of candidate ligands within a short period of time, which is a major advantage of virtual screening [82,83,84,85].

Based on our computational screening results, three potent inhibitors targeting the SARS-CoV-2 M^pro^ were identified that exhibit significant binding affinities and interaction with the active groove of the target protein, which is vital for targeted M^pro^ inhibition. Our first compound, epicatechin-3-O-gallate, was found to have anti-melanogenic effects [86], acts as a chemopreventative for multistage carcinogenesis [87], plays a crucial role in Alzheimer disease through the inhibition of aβ aggerates formation [88], and inhibits cell invasion and protease activity [89] within the human body. Furthermore, it can chelate Zn^2+^ and Cu^2+^ to lessen reactive oxygen species production, which can alleviate neurotoxicity induced by Cu^2+^- and Zn^2+^-Aβ40 [88]. The availability of the antimetastatic effect, the suppression of tumor growth in the A549 cells [89], along with the competency to pass through the blood–brain impediment [88], has dispensed therapeutic impacts of epicatechin-3-O-gallate as an anti-invasion and anti-cancer agent [89]. This phytochemical formed multiple non-covalent bonds with M^pro^, including five interactions that were observed in the active site of M^pro^: His164 (hydrogen), Asn142 (hydrogen), Gln189 (hydrogen), Met165 (pi-alkyl), and Pro168 (pi-alkyl). Post-molecular dynamics interactions also confirmed several interactions with active site residues with precise rigidity, which may be responsible for the inhibition [90].

The phytochemical psi-taraxasterol has been associated with multiple functions in the cell and enzymatic assays, including anti-edematous activity [91], inflammation, and the anti-inflammatory response [92]. This compound formed numerous non-bonded interactions at the active site (Met165, Met49, Cys145, His41) of M^pro^, both before and after the molecular dynamics simulation. Although this compound had a relatively higher distance in docking interactions at 0 ns, the interactions remained stable across the simulation trajectories. Moreover, their interactions were driven by alkyl and pi-alkyl interactions, but at the last simulation trajectories, they were seen to form more hydrogen bond and hydrophobic interactions at the active sites by relatively lower distance. These non-covalent interactions by engaging the π systems are crucial for the ligand–protein interactions [93]. Therefore, the interacting residues of these complexes seem to bind tightly after specific periods of simulation time, which provides crucial insights into the rigid binding of this complex. Therefore, this compound may undergo some degree of conformational changes to the docking snapshots, which ultimately led to the tight bindings after 100-ns times.

In addition, catechin gallate has shown significant effect as an antimalarial agent [94] and in the lipogenesis pathway [95] and demonstrated potential and much heftier anti-proliferative and anti-inflammatory actions in pancreatic tumor cells [96]. This phytochemical can suppress the activity of HIV-1 integrase. Moreover, it may lessen HIV-1 integrase activity by shattering its influence with virus DNA [97]. Besides, catechin gallate can inhibit consumption of methyl glucose by adipocytes [98]. The catechin gallate–M^pro^ complex interacted at residues within the active cavity, including Leu141, Thr190, Gln189, His41, Met165, Cys145, Met49, and Glu166, both before and after the molecular dynamics simulation.

The virtual screening and molecular docking study were also conducted against the main protease of SARS-CoV-2 by taking FDA-approved drugs from the ZINC database where they have found binding energy ranges from −7.8 to −6.839 kcal/mol [99], whereas we have found epicatechin-3-O-gallate, psi-taraxasterol, and catechin gallate having binding affinities of −8.4, −8.5, and −8.8 kcal/mol. Additionally, multiple studies regarding the virtual screening and molecular docking were conducted by targeting the main protease of SARS-CoV-2 where several interactions at the active points Thr26, Leu41, Cys145, Pro168, Asn142, His164, Met49, Phe140, Met165, Glu166, Gln189, and Thr190 [99,100,101,102,103] were observed, which is similar to our findings. 

The molecular dynamics simulation study was performed for the best-studied complexes to support the molecular docking assessment, and multiple descriptors from the simulation trajectories were assessed to identify binding rigidity. The RMSDs of the docked complexes were all below 2.5 Å for the C-alpha atom, although psi-taraxasterol had comparatively higher flexibility. The RMSF of the amino acid residues confirmed the less flexible area in Domain 2, with slightly more flexibility observed for Domains 1 and 3 of M^pro^. The protein volume for the three docked complexes did not show any alterations in the SASA profiles during the simulation trajectories for all three complexes, which had low degrees of variation. The quantitative assessment of hydrogen bond patterns and the Rg profiles of these three complexes were similar to other descriptors of simulations.

**Figure 4:**
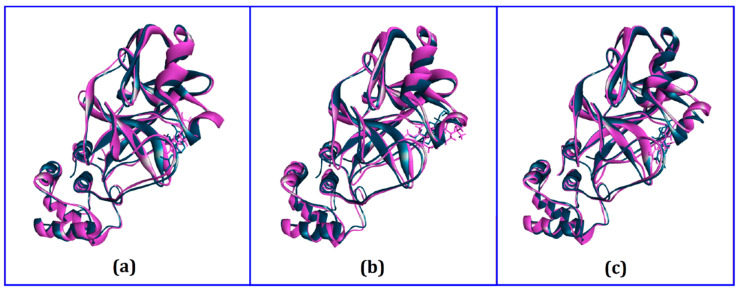
Superimpositions of the drug–protein complex before and after molecular dynamics. Purple color indicates the baseline structure and dark blue indicates the structure after molecular dynamics. (**a**) Epicatechin-3-O-gallate, (**b**) Psi-taraxasterol, and (**c**) Catechin gallate.

**Figure 5:**
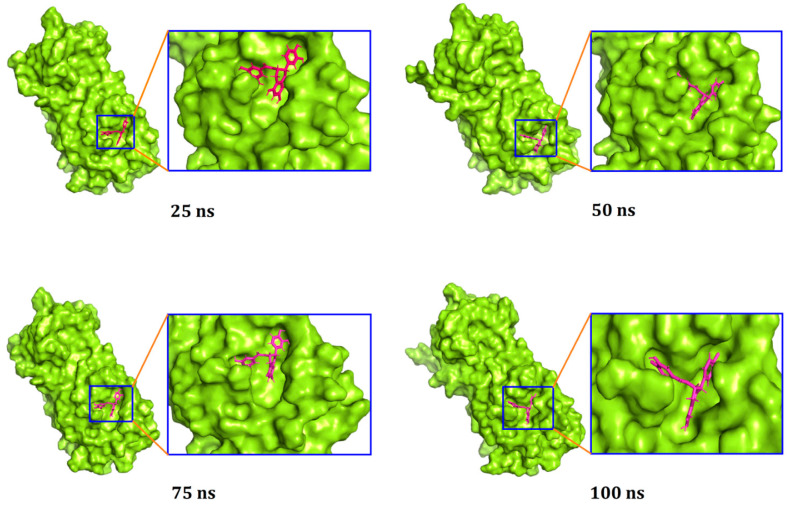
Surface views for the docked complex in the molecular dynamics simulation. The snapshots were taken at 25, 50, 75, and 100 ns, respectively, for (+)-epicatechin-3-O-gallate and the M^pro^ complex.

**Figure 6:**
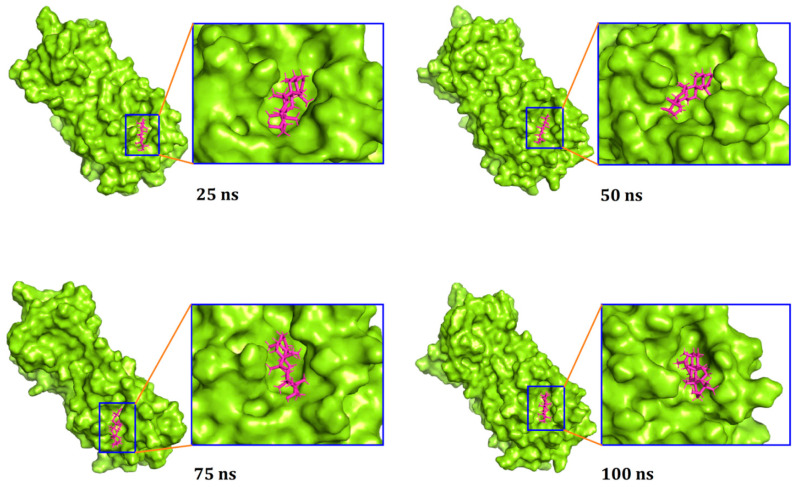
The surface view and the binding pockets between psi-taraxasterol and M^pro^ complex, with snapshots at 25, 50, 75, and 100 ns.

**Figure 7:**
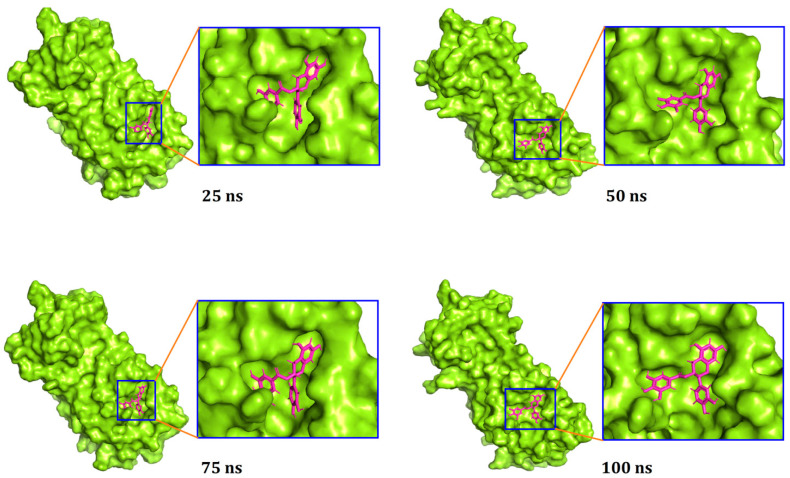
The surface view of the docked (-)-catechin gallate and M^pro^ complex, with snapshots at 25, 50, 75, and 100 ns.

Moreover, a superimposition of the structures before and after molecular dynamics was performed (Figure 4) to determine changes in the binding cavity. The top screened complexes, including epicatechin-3-O-gallate, psi-taraxasterol, and catechin gallate, had RMSD values of 1.25, 1.78, and 1.65 Å, respectively. We also took snapshots from different simulation trajectories, at 25, 50, 75, and 100 ns, for the three top complexes, and no radical alterations were observed for all three complexes (Figure 5, Figure 6 and Figure 7).

## 5. Conclusions

In this workflow, we first short-listed 1672 plant-based phytochemicals through deep literature mining. The compounds were screened against the SARS-CoV-2 M^pro^ to identify potent inhibitors that might be able to interfere with the catalytic function of M^pro^. The virtual screening and molecular docking studies identified three potent inhibitors: epicatechin-3-O-gallate, psi-taraxasterol, and catechin gallate from several large, compound data sets. Furthermore, the molecular simulation study of the docked complexes supported the stable nature and rigid conformations formed by the docked complexes, as assessed by the simulation trajectories and based on multiple descriptor analyses. The binding interactions were confirmed at several active sites, including His41, Cys145, Met49, and Glu166, and their stiffness was confirmed after molecular dynamics. This combinatorial screening was solely based on computational pipelines; therefore, additional in vitro assays must be performed to confirm the precise targeting of these compounds against SARS-CoV-2.

## Figures and Tables

**Table 1:** Non-bond interactions between SARS-CoV-2 main protease and the top three (3) compounds.

Compounds	PubChem CID	Binding Affinity (kcal/mol)	Residues in Contact	Interaction Type	Distance in Å
Epicatechin-3-O-gallate	65056	−8.4	HIS164	Conventional hydrogen bond	2.51938
			HIS163	Conventional hydrogen bond	2.28965
			ASN142	Conventional hydrogen bond	2.57732
			GLN189	Carbon hydrogen bond	2.19623
			MET165	Pi-alkyl	5.3972
			PRO168	Pi-alkyl	5.49119
Psi-taraxasterol	5270605	−8.5	MET165	Alkyl	5.04103
			MET49	Alkyl	3.86071
			CYS145	Alkyl	5.38342
			HIS41	Pi-alkyl	5.35384
Catechin gallate	6419835	−8.8	LEU141	Conventional hydrogen bond	2.05522
			HIS163	Conventional hydrogen bond	1.92422
			ARG188	Conventional hydrogen bond	2.46163
			THR190	Conventional hydrogen bond	2.05509
			GLN189	Carbon hydrogen bond	2.3535
			HIS41	Pi-Pi T-shaped	5.22838
			MET165	Pi-alkyl	4.91554
			CYS145	Pi-alkyl	5.4117
			MET49	Pi-alkyl	5.21898

**Table 2:** Pharmacological profiles for the top three potential candidates, which were derived from the SwissADME, admetSAR, and pKCSM webservers.

Parameters	Epicatechin-3-O-gallate	Psi-taraxasterol	Catechin gallate
Molecular weight	442.4 g/moL	426.7 g/moL	442.4 g/moL
H-bond acceptor	10	1	10
H-bond donor	7	1	7
CNS	−3.743	−1.992	−3.743
CYP2D6 substrate	No	No	No
CYP3A4 substrate	No	Yes	No
CYP1A2 inhibitor	No	No	No
CYP2C19 inhibitor	No	No	No
CYP2C9 inhibitor	No	No	No
CYP2D6 inhibitor	No	No	No
CYP3A4 inhibitor	No	No	No
Carcinogenicity	Non-carcinogenic	Non-carcinogenic	Non-carcinogenic
Hepatotoxicity	No	No	No
P-glycoprotein inhibitor	No	No	No
Human intestinal absorption	+0.9942	+0.9919	+0.9942
Ames mutagenesis	−0.5000	−0.8500	−0.5000
Acute oral toxicity	No	No	No
Lipinski rule of five	Yes	Yes	Yes

**Table 3:** The binding interactions of the docked complex after 100 ns in the molecular dynamics simulation study. H indicates hydrogen and PI indicates pi-alkyl bond.

Complex	Residues	Interactions	Distance
Epicatechin-3-O-gallate	Ser144 His163 Phe140 Cys145 His41 Leu141 Met49 Cys165	H H H H H PA PA PA	1.78 2.73 1.96 2.50 2.89 4.21 5.18 5.23
Psi-taraxasterol	Cys145 Met165 Met49 His41	H H H PA	1.23 2.21 2.73 4.33
Catechin gallate	Asn142 Leu141 Glu166 Met49 Val186 Arg188 Met165 Cys145 His41	H H H H H H H PA PA	1.81 2.24 1.50 3.03 2.92 1.83 2.39 5.21 5.53

## Data Availability

Available data are presented in the manuscript.

## Supplementary Materials

The following are available online: https://www.mdpi.com/article/10.3390/biology10070589/s1. Table S1: A total of 1672 compounds used in the virtual screening from 32 plants. Table S2: A total of 1672 compounds used in the virtual screening from 10 plants. Table S3: A total of 1672 compounds used in the virtual screening from 14 plants.
