# Supplementary material for: Plant-Based Phytochemical Screening by Targeting Main Protease of SARS-CoV-2 to Design Effective Potent Inhibitors
Source: Biology (Basel). 2021 Jun 26;10(7):589. doi: 10.3390/biology10070589 (PMC8301192; doi:10.3390/biology10070589)
Supplement: Supplementary file 1 [file biology-10-00589-s001.zip › biology-1251035-supplementary.pdf]

Serial No.	Plant name	Chemical name	Pubchem CID
01	*Abelmoschus manihot* var. *pungens* (Roxb.) Hochr	Quercetin-3-O-Robinobioside[1]	10371536
		Hyperin[1]	5281643
		Hibifolin[1]	5490334
		Quercetin-3'-O- Glucoside[1]	10006384
		Quercetin[1]	5280343
		Isoquercetin[1]	5280804
		Myricetin[1]	5281672
		Adenosine[2]	60961
		Stigmasterol[2]	5280794
		DPPH[2]	74358
02	*Achillea millefolium* Linn.	Cynaroside[3]	5280637
		Centaureidin[3]	5315773
		Apigenin[3]	5280443
		Artemetin[3]	5320351
		Carvacrol[3]	10364
		Cosmosiin[3]	5280704
		Casticin[3]	5315263
		Luteolin[3]	5280445
		Thymol[3]	6989
		Limonene[3]	440917
		Camphene[3]	6616
		Caffeic acid[3]	689043
		Achillinin A[3]	101805792
		Rutin[3]	5280805

		1,8-cineole[3]	2758
		Bisabolol[3]	1549992
		α-pinene[3]	6654
		β-pinene[3]	14896
		Germacrene D[3]	5373727
		Camazulene[3]	10719
		Achilleine[4]	457906
		Trigonelline[4]	5570
		Betonicine[4]	164642
		Alpha-terpineol[4]	17100
		Camphor[4]	2537
		Borneol[4]	64685
		Stachydrine[5]	115244
		Linalool[5]	6549
		Azulene[5]	9231
		Sabinene[5]	18818
03	*Achyranthes aspera* Linn	Oleanolic acid[6]	10494
		D-Glucuronic Acid[6]	94715
		6-pentatriacontanone[6]	129687427
		Hexatriacontane[6]	12412
		Tritriacontane[6]	12411
		Ecdysterone[6]	11081347
		Ferulic acid[7]	445858

		Apigenin[7]	5280443
		Salicylic acid[7]	338
		Azelaic acid[7]	2266
		Linolenic acid[7]	5280934
		Undecanedioic acid[7]	15816
		Dodecenedioic acid[7]	22904
		Traumatic acid[7]	5283028
		Lauric acid[7]	3893
		13-Hydroxy-9,11-octadecadienoic acid[7]	5282948
		Protocatechuic acid[7]	72
		Gentisic acid[7]	3469
		Caffeic acid[7]	689043
		Kaempferol-3-O-glucoside[7]	5282102
		Tiliroside[7]	5320686
		Roseoside corchoionoside C[7]	9930064
		Rubrosterone[7]	12315102
		20-Hydroxyecdysone[8]	5459840
		Myristic acid[8]	11005
		Palmitic acid[8]	985
		Stearic acid[8]	5281
		Arachidic acid[8]	10467
		Oleic acid[8]	445639

04	*Acmella calva* (DC.) R.K.Jansen	Quinone[9]	4650
		Tannin[9]	250395
		Saponin[9]	198016
		Spilanthol[10]	530394
05	*Aconitum austroyunnanense* W.T. Wang	Aconitine[11]	245005
		Mesaconitine[11]	441747
		Jesaconitine[11]	441741
		Hypaconitine[11]	441737
		Benzoylaconine[11]	20055771
		Benzoylmesaconine[11]	24832659
		Benzoylhypaconine[11]	13343337
		Heteratisine[12]	73527
		Heterophyllisine[12]	101289617
		Heterophylline[12]	251575
		Heterophyllidine[12]	132491405
		Atidine[12]	12299861
		Hetidine[12]	101685340
06	*Aconitum brachypodum* Diels	Pyrocatechol[13]	289
		Flavone[13]	10680
		Lappaconitine[14]	90479327
		Yunaconitine[14]	6918110

07	*Aconitum carmichaelii* Debx.	Hypaconine[15]	101671038
		Mesaconine[15]	101671037
		Beiwutinine[15]	101552718
		N-deethylaconine[15]	273986295
		Fuziline[16]	14163819
		Neoline[16]	120682
		Oleracein E[16]	21574476
		Isodelphinine[17]	102146471
		Benzoylmesaconitine[17]	122173204
		Coryneine[17]	165581
		Atisine[17]	441709
		Higenamine[17]	114840
		Salsolinol[17]	91588
		Chasmanine[14]	20055812
		Crassicauline A[14]	20055838
		Senbusine A[14]	158048
		Songoramine[14]	14526618
		Oxonitine[14]	6708531
		Deoxyaconitine[14]	44445634
08	*Aconitum scaposum Franch*.var.*hupehanum* Rapaics	Acetylaconitine[18]	21599000
		Napelline[18]	441749
		6-benzoylheteratisine[18]	5487064

		Songorine[18]	71456946
09	*Acorus calamus*	Xanthone[19]	7020
		Lignans[19]	443013
		Calameon[19]	181982
		camphene[19]	6616
		β- asarone[19]	5281758
		α- asarone[19]	636822
		Elemicin[19]	10248
		Cis isoeugenol	1549041
		P-cymene[19]	7463
		α- selinene[19]	10856614
		β- cadinene[19]	10657
		Camphor[19]	2537
		Terpinen- 4-ol[19]	11230
		Acorone[19]	5316254
		Acoragermacrone[19]	6452183
		Linalool[19]	6549
		preisocalamendiol[19]	85437819
		Acoradin[19]	126324
		Galangin[19]	5281616
		2, 4, 5- Trimethoxy benzaldehyde[19]	20525
		Calamendiol[19]	12302239
		Spathulenol[19]	92231
		Calarene[19]	28481
		Isoshyobunone[19]	5318673

		4- Terpineol[19]	11230
		2-Allyl-5-ethoxy-4- methoxyphenol[19]	596924
		Epieudesmin[19]	7299790
		Lysidine[19]	10798
		Nonanoic Acid[19]	8158
		2,2,5,5-Tetramethyl-3-hexanol[19]	230736
		Bornyl acetate[19]	6448
		Galgravin[19]	101749
		Retusin[19]	5352005
		Sakuranin[19]	73607
		Acetic acid[19]	176
		Isoelemicin[19]	5318557
		Acetophenone[19]	7410
		Dehydroabietic acid[19]	94391
		Dehydrodiisoeugenol	5379033
		Acorenone[19]	12480741
		Isocalamendiol[19]	12302240
		Eugenol[19]	3314
		Acorafuran[19]	91751248
		Shyobunone[19]	5321293
		Epishyobunone[19]	591309
		Myrcene[19]	31253
		β-phellandrene[19]	11142

		Terpinolene[19]	11463
		Thujane[19]	79017
		Limonene[19]	22311
		Methylisoeugenol[19]	637776
		Cedrol[19]	65575
		Dehydrofukinone[19]	177072
		β -Cyclocitral[20]	9895
		Longifolene[20]	289151
		Gamma -Asarone[20]	636750
		Eremophilone[20]	21591457
		Nootkatone[20]	1268142
10	*Acorus gramineus* Sol. ex Aiton	Methyl chavicol[21]	66957732
		Octanediol[21]	17828881
		Pyrrole[22]	8027
		Lignans[22]	443013
		4-(2-Formyl-5-(methoxymethyl)-1H-pyrrol-1-yl)butanoic acid[22]	10059539
		γ-aminobutyric acid[23]	119
		Palmitic acid[23]	985
		Phenol[23]	996
		Palmitin[23]	133065452
		β-asarone[24]	5281758
		α-asarone[24]	636822

		γ -asarone[24]	636750
		Acoramol[25]	70682800
		Magnosalin[25]	10454589
		Humulene[26]	5281520
		4- Allylanisole[26]	8815
11	*Adiantum bonatianum* Brause	Triterpenoids[27]	71597391
		Diploptene[27]	92155
		Adininaneone[27]	101170704
		Hydroxyhopane[27]	397488
		Mollugogenol A[27]	193011
		Neohopene[27]	13857695
		Glaucanol B acetate[27]	101700567
		Hydroxyadiantone[27]	21159071
		Adiantone[27]	15558363
		Adipedatol[27]	12127456
		Filicenal[27]	101596938
		Fernene[27]	441679
		Pyridine[28]	1049
		P-Cresol[28]	2879

12	*Aesculus chinensis* Bunge	Indoles[28]	139191468
		Maltol[28]	8369
		Furfural[28]	7362
		2-Pentanamine[28]	12246
		Ethyne[28]	6326
		1,4-Dioxane-2,6-dione[28]	78232
		Diazene[28]	123195
		Acetic acid[28]	176
		Acetic acid, methyl ester[28]	6584
		1,3-Cyclopentadiene[28]	7612
		Propanal[28]	527
		1-Propanol[28]	1031
		2,3-Butanedione[28]	650
		(Z)-1,3-Butadien-1-ol[28]	5362846
		2-Propanone[28]	180
		Ethanone[28]	41122
		Acetamide, N-(aminoiminomethyl)[28]	136295065
		3-Butenoic acid[28]	32743
		2,3-Pentanedione[28]	11747
		1,2-Ethanediol[28]	174
		Furan[28]	8029
		Propanoic acid[28]	1032
		Acetamide, N-2-propynyl[28]	240796

		Propanoic acid, 2-oxo-, methyl ester[28]	12166644
		1H-Pyrrole[28]	8027
		1H-Pyrrole, 1-methyl[28]	5380833
		N,N-Dimethylaminoethanol[28]	7902
		2-Propanone, 1-hydroxy[28]	586459
		2-Butenal[28]	447466
		2-Butenal, 2-methyl-, (E)-[28]	5321950
		Cyclopentanone[28]	8452
		3-Amino-s-triazole[28]	122363
		4-Methyl-3,7,9-trioxabicyclo (4,2,1) nonane[28]	538221
		3-Furaldehyde[28]	10351
		Pyrrolidine[28]	31268
		Pyridine, 2-methyl[28]	15123265
		Pyrazine[28]	9261
		2-Vinylethyl acetate[28]	74095
		2-Cyclopenten-1-one[28]	13588
		1H-Pyrrole, 3-methyl[28]	13169091
		1-Butene, 3-methyl[28]	11239
		2-Furanmethanol[28]	7361
		Benzene, 1,3-dimethyl[28]	15531006
		2(3H)-Furanone, 5-methyl[28]	19826
		Carbamic acid, phenyl ester[28]	69322
		Cyclopent-4-ene-1,3-dione[28]	70258

		Styrene[28]	7501
		2-Butenoic acid, methyl ester, (E)-[28]	638132
		2-Cyclopenten-1-one, 2-methyl-[28]	14266
		Ethanone, 1-(2-furanyl)-[28]	14505
		2(5H)-Furanone[28]	10341
		Diisoamyl ether[28]	10989
		2-Cyclopenten-1-one, 2-hydroxy[28]	528702
		2(5H)-Furanone, 5-methyl[28]	11558
		2,5-Furandione, dihydro-3-methylene[28]	75110
		Cycloheptanone[28]	10400
		Thiazole[28]	9256
		1,4-Pentanediol[28]	79083
		Phenol[28]	996
		2-Furancarboxaldehyde[28]	7362
		Hexanoic acid[28]	8892
		Aziridine, 2-methyl-3-(1-methylethyl)-, trans[28]	544210
		Aziridine[28]	9033
		3-Amino-1,2,4-triazole-5-carboxylic acid[28]	77200
		3-Cyclobutene-1,2-dione, 3,4-dihydroxy[28]	17913
		2-Furanone, 2,5-dihydro-3,5-dimethyl[28]	318158
		1H-Pyrrole, 2-ethyl-4-methyl[28]	522354
		6-Azacytosine[28]	70265
		Tricyclo[4.2.1.1 (2,5)]decane[28]	556700

		2-Cyano-1-hexene[28]	524765
		1,3-Dimethyl-1-cyclohexene[28]	137726
		2-Propyl-1-pentanol, pentafluoropropionate[28]	91692872
		1,2-Cyclopentanedione, 3-methyl[28]	61209
		3-Heptyne, 2,2-dimethyl[28]	572970
		4-Methyl-5H-furan-2-one[28]	145832
		1,3-Dioxol-2-one, 4,5-dimethyl[28]	142210
		2-Cyclopenten-1-one, 2-hydroxy-3,4-dimethyl[28]	89539
		Ethanone, 1-(1H-pyrrol-2-yl)-[28]	14079
		2,5-Dimethylfuran-3,4(2H,5H)-dione[28]	10154060
		Acetophenone[28]	7410
		2-Cyclopenten-1-one, 3-ethyl[28]	557256
		Heptanoic acid[28]	8094
		Valeraldehyde, 2,2-dimethyl-, oxime[28]	9601757
		Furyl hydroxymethyl ketone[28]	519466
		6-Amino-1,3,5-triazine-2,4(1H,3H)-dione[28]	12584
		Cyclohexanol[28]	7966
		Diazene, [1-(2,2-dimethylhydrazino) ethyl] ethyl[28]	541807
		Propanoic acid, 2-chloro-, pentyl ester[28]	522861
		3-Pyridinol[28]	7971
		2-Cyclopenten-1-one, 3-ethyl-2-hydroxy[28]	62752
		10-Methylundecan-4-olide[28]	21778194

		5-[3-Methyl-2-furyl] hydantoin[28]	572997
		(1,4,4-Trimethyl-cyclohex-2-enyl)-acetic acid[28]	572311
		Formaldehyde, methyl(2-propynyl) hydrazone[28]	536902
		1,4-Benzodioxin, octahydro-2-methylene-, trans[28]	38079
		4H-Pyran-4-one, 2,3-dihydro-3,5-dihydroxy-6-methyl[28]	119838
		3(2H)-Furanone, dihydro-5-methyl[28]	520667
		Tetracyclo[5.3.0.0.0]deca-4,8-diene[28]	141895
		2H-Pyran-2-one, tetrahydro-3,5-dimethyl[28]	544776
		5,8-Decadien-2-one, 5,9-dimethyl-, (E)-[28]	5369930
		2-Butenoic acid, 3-amino-, ethyl ester[28]	95308
		Octanoic acid[28]	379
		2-Penten-1-ol, (E)-[28]	5364920
		Pentyl acetoacetate[28]	138786
		7-Thiabicyclo[4.2.1]nonane[28]	559235
		Creosol[28]	7144
		4,7,7-Trimethyl-5-(tetrahydropyran-2-yloxy)-bicyclo[2.2.1]heptan-2-one[28]	559248
		Catechol[28]	289
		1,4:3,6-Dianhydro-.alpha.-d-glucopyranose[28]	22213879
		Benzofuran, 2,3-dihydro[28]	13664477
		Heptadecanal[28]	71552
		2,3-Anhydro-d-mannosan[28]	88413508
		2-Isopropoxyphenol[28]	20949

		Chrysanthemyl 2-hydroxy-3-methylbutanoate[28]	91711065
		Carbamic acid, (4-nitrophenyl)-, phenylmethyl ester[28]	298762
		2-Cyclohexen-1-one, 3,4,4-trimethyl[28]	28450
		5,6,6-Trimethyl-5-(3-oxobut-1-enyl)-1-oxaspiro[2.5]octan-4-one[28]	5363139
		Benzene, 1,3-dimethoxy-2-nitro[28]	324784
		7-Oxabicyclo[4.1.0]heptane, 2-methylene[28]	534042
		Phenol, 4-ethyl-2-methoxy[28]	183540
		Isopropylphosphonic acid, dimethyl ester[28]	573520
		1,3,5,7-Cyclooctatetraene[28]	637866
		1,2-Benzenediol, 4-methyl[28]	14759971
		Indole[28]	798
		4-Acetoxy-1,2,3,5,6,7,8,8a-octahydroazulene[28]	539828
		2-Methoxy-4-vinylphenol[28]	332
		8-Tetradecyn-1-ol[28]	573432
		Globulol[28]	101716
		2,4-Diaminophenol[28]	7266
		Pyran, tetrahydro-2-(3-bromo-5,5-dimethyl-2-cyclohexenyl) oxy[28]	559258
		1H-Isoindole-1,3(2H)-dione, 3a, 4,5,7a-tetrahydro-4-hydroxy-3a-methyl-2-phenyl-, [3aR-(3a.alpha.,4.beta.,7a.alpha.)][28]	573576
		Eugenol[28]	3314
		1 3-Amino-2,6-dimethoxypyridine[28]	593005

		Phenol, 2-methoxy-4-propyl[28]	3084581
		9-Borabicyclo[3.3.1]nonane, 9-(1-ethylbutyl)-[28]	535156
		1H-Imidazole-4-methanol, 5-methyl[28]	122433
		2,4-Hexadiene, 2,5-dimethyl[28]	12992
		1-Benzoxepin-2(3H)-one, octahydro[28]	558564
		11-Bromoundecanoic acid[28]	17812
		4,5,6-Trimethyl-2-pyrimidone[28]	586611
		meta-Methoxybenzenethiol[28]	84989
		Vanillin[28]	1183
		3-Allyl-6-methoxyphenol[28]	596375
		Tetrahydroionone[28]	110787
		4-Nitro-3-picoline-N-oxide[28]	70633
		Benzenamine[28]	6115
		1-Tetradecene, 14-bromo[28]	543846
		3,5-Dimethoxy-4-hydroxytoluene[28]	240925
		Trans-Isoeugenol[28]	853433
		Spiro[4.5]decan-7-one, 1,8-dimethyl-8,9-epoxy-4-isopropyl[28]	538938
		Bicyclo (3.3.1) nonane-2,6-dione[28]	140084
		Bicyclo[3.1.0]hexane-6-methanol, 2-hydroxy-1,4,4-trimethyl[28]	538067
		Oleic Acid[28]	445639
		5-Hepten-3-yn-2-ol, 6-methyl-5-(1-methylethyl)-[28]	591903

		.beta.-D-Glucopyranose, 1,6-anhydro[28]	11947765
		D-Allose[28]	439507
		2-Propanone, 1-(4-hydroxy-3-methoxyphenyl)-[28]	17262
		Dodecanoic acid, 1-methylethyl ester[28]	25068
		2,3,5,6-Tetrafluoroanisole[28]	75351
		2-Hydroxy-2,4,4-trimethyl-3-(3-methylbuta-1,3-dienyl) cyclohexanone[28]	5363147
		10-Methyldodec-2-en-4-olide[28]	21778198
		Benzofuran-4(5H)-one, 6,7-dihydro-, oxime[28]	6537039
		Phenol, 2,6-dimethoxy-4-(2-propenyl)-[28]	226486
		Pyrazole-5-carboxylic acid, 1-ethyl-4-nitro[28]	597434
		Benzeneethanamine, 2-fluoro-4,5-dimethoxy-.beta.-hydroxy-N-isopropyl-[28]	551617
		1-Chloroeicosane[28]	39150
		5,7-Dimethyl-1,3-diazaadamantan-6-one Hydrazone[28]	606599
		2-Pentadecanone, 6,10,14-trimethyl[28]	10408
		4-((1E)-3-Hydroxy-1-propenyl)-2-methoxyphenol[28]	1549095
		2,4,5-Tri-O-acetyl-3,6-di-O-methyl-D-mannonitrile[28]	560083
		Octyltrichlorosilane[28]	21354
		2-Trimethylsilyl-1,3-dithiane[28]	83413
		1,3-Benzenediol, 2,5-dimethyl[28]	68103
		(E)-2,6-Dimethoxy-4-(prop-1-en-1-yl) phenol[28]	5352905

		D-Norleucine, N-isobutoxycarbonyl-, ethyl ester[28]	91729851
		Ethanone, 1-(4-hydroxy-3,5-dimethoxyphenyl)-[28]	17198
		2(3H)-Naphthalenone, 4,4a, 5,6,7,8-hexahydro-1-methoxy[28]	534313
		Tetrazolo[1,5-a] pyrimidine, 6-(3-hydroxy-2-oxopropyl)-[28]	543051
		2-Pentanone, 1-(2,4,6-trihydroxyphenyl)[28]	597628
		Cyclotetradecane[28]	67524
		Hexadecenoic acid, Z-11-[28]	5312414
		Neophytadiene[28]	10446
		cis-7, cis-11-Hexadecadien-1-yl acetate[28]	5363265
		2-Cyclohexen-1-one, 3-(3-hydroxybutyl)-2,4,4-trimethyl[28]	520295
		Pentadecanoic acid[28]	13849
		Docosanoic acid[28]	8215
		E-8-Methyl-9-tetradecen-1-ol acetate[28]	5363273
		p-Phenylenediamine, N-benzylidene-N’-phenyl[28]	229937
		Pyrimidine-5-carbonitrile, 3,4-dihydro-6-(dimethylaminophenyl)-2-mercapto-4-oxo[28]	787657
		19-Norandrost-4-en-3,17-dione (8.beta.,9.beta.,10.alpha.)[28]	623313
		cis-1-Chloro-9-octadecene[28]	5367784
		n-Hexadecanoic acid[28]	985
		Scopoletin[28]	5280460
		Dibutyl phthalate[28]	3026
		Escin Ib[29]	6476031
		Escin Ia[29]	6476030

		Isoescin Ia[29]	6476032
		Isoescin Ib[29]	6476033
13	*Agapetes hosseana* Diels	β-sitosterol[30]	222284
		Friedelanol[30]	101341
		Friedelin[30]	91472
		Lupeol[31]	259846
		Taraxerol[31]	92097
		Taraxerone[31]	92785
		α-amyrin[31]	73170
		β-amyrin[31]	73145
		3α-friedelanol[31]	102076594
		β-amyrenonol[31]	20055661
		Isolariciresinol[31]	160521
		Epicatechin[31]	72276
		Lupeol benzoate[31]	219629
		Lupeol cinnamate[31]	5471662
		β-sitosterol benzoate[31]	102239972
		3-acetyl ursolic acid[31]	234102
		Fernenol[32]	12305178
		Daucosterol[32]	5742590

14	*Agastache rugosa* (Fisch. et Mey.) O. Kuntze	Methyl chavicol[33]	8815
		Acacetin[33]	5280442
		Tilianin[33]	5321954
		Agastachoside[33]	52857846
		Linarin[33]	5317025
		Agastachin[33]	51014580
		Maslinic acid[33]	73659
		Oleanolic acid[33]	10494
		Daucosterol[33]	5742590
		β-sitosterol[33]	222284
		Dehydroagastol[33]	126634
		3-O- acetyloleanolic acid[33]	151202
		Agastenol[33]	10073783
		Linolenic acid[33]	5280934
		Palmitic acid[33]	985
		Lauric acid[33]	3893
		Linoleic acid[33]	5280450
		Rosmarinic acid[33]	5281792
		Methyl eugenol[33]	7127
		Safrole[33]	5144
		β-pinene[33]	14896

		α-pinene[33]	6654
		Limonene[33]	22311
		P-cymene[33]	7463
		3-Octanone[33]	246728
		3-Octanol[33]	11527
		1-octen-3-ol[33]	18827
		Linalool[33]	6549
		β-elemene[33]	6918391
		β-caryophyllene[33]	5281515
		α-humulene[33]	5281520
		δ-cadinene[33]	441005
		α-ylangene[33]	442409
		β-humulene[33]	5318102
		β-farnesene[33]	10407
		γ-cadinene[33]	6432404
		Calamenene[33]	6429077
		Anisaldehyde[33]	31244
		Eugenol[33]	3314
		Anethol[33]	637563
		β-bourbonene[33]	324224

		Bornyl acetate[33]	6448
		α-cadinol[33]	10398656
		Damascenone[33]	5366074
		β-ionone[33]	638014
		Isomenthone[33]	6432469
		Myrcene[33]	31253
		(Z)-β-ocimene[33]	5320250
		7-octen-4-ol[33]	40923
		Pulegone[33]	442495
		Spathulenol[33]	92231
		1,8-cineole[33]	2758
		1-octen-3-yl acetate[33]	17121
		Camphor[33]	2537
		Menthone[33]	26447
		Rosefuran epoxide[33]	6428926
		Menthol[33]	1254
		Carvone[33]	7439
		Piperitone[33]	6987
		Menthyl acetate[33]	27867
		Dihydrocarvyl acetate[33]	30248
		2-methoxycinnamaldehyde[33]	641298

		(Z)-nerolidol[33]	5320128
		Caryophyllene oxide[33]	1742210
		δ-cadinol[33]	3084311
		Thymol[34]	6989
		D-Limonene[34]	440917
		γ-Terpinene[34]	7461
		α-Terpineol[34]	17100
		Cuminic alcohol[34]	325
		Elixene[34]	94254
		Carvacrol[34]	10364
		α-Cubebene[34]	86609
		γ-Muurolene[34]	12313020
		Germacrene D[34]	5317570
		Aromadendrene[34]	91354
		Bicyclogermacrene[34]	13894537
		α-Muurolene[34]	12306047
		α-Farnesene[34]	5281516
		Cadina-4,9-diene[34]	91748288
		Germacrene B[34]	5281519
		Spatulenol[34]	13854258
		Viridiflorol[34]	11996452
		τ-Muurolol[34]	3084331
		Acetophenone[34]	7410

		Thymoquinone[34]	10281
		Methyl hexadecanoate[35]	8181
		Ursolic acid[35]	64945
		Apigenin[35]	5280443
		Protocatechuic acid[35]	72
		Agastinol[36]	637406
		P-menthan-3-one[37]	6986
		Agastaquinone[37]	177257
		Corosolic acid[37]	6918774
		4′,5-Dihydroxy-3,3′,7-trimethoxyflavone[37]	5281677
15	*Agave sisalana* Perrine ex Engelm.	Hecogenin[38]	91453
		Gitogenin[38]	441887
		Tigogenin[38]	99516
		Neotigogenin[38]	12304433
		9(11)-dehydrohecogenin[38]	15011079
		5,7-dihydroxyflavanone[38]	238782
		kaempferol 3- rutinoside-4-glucoside[38]	44258844
		7-O-methyleucomol[38]	124355924
		3-deoxysappanone[38]	57391100
		(±)-3,9-dihydroeucomin[38]	11415348
		5,7-dihydroxy-3-(3-hydroxy-4-methoxybenzyl)-4-chromanone[38]	404571

		Dihydrobonducellin[38]	51136567
		5,7-dihydroxy-3-(4- hydroxybenzyl)-4-chromanone[38]	9971218
		Dongnoside B[39]	192451
		Dongnoside A[39]	44149635
		5,7-dihydroxyflavone[39]	5281607
		7-hydroxy-3-(4- hydroxybenzyl) chromane[39]	11708657
		Inulin[40]	24763
		Sisalagenin[40]	12305694
		Rockogenin[40]	167555
		Chlorogenin[40]	12303065
		β-sitosterol[40]	222284
		Hongguanggenin[40]	52931465
		Pectin[41]	441476
		Mannitol[41]	6251
		Succinic acid[41]	1110
		Kaempferol[41]	5280863
		Neohecogenin[42]	90473944
		Polianthoside E[42]	11434838
		Coumarin[43]	323
		2-deoxo-2-(acetyloxy)-9-oxoageraphorone[43]	124222289
		Euptox A[43]	14485504

16	*Ageratina adenophora* (Spreng.) R. M. King et H. Rob.	Muurol-4-en-7-ol[43]	102095305
		Chlorogenic acid[43]	1794427
		Neochlorogenic acid[43]	5280633
		Cryptochlorogenic acid[43]	9798666
		Chlorogenic acid methyl ester[43]	6476139
		Diacetone alcohol[43]	31256
		Bicyclo[2.2.1]heptane, 2,2-dimethyl-3-methylene-[43]	6616
		p-Mentha-1,4(8)-diene[43]	11463
		Benzene, methyl(1-methylethyl)-[43]	7463
		p-Mentha-1,5-diene[43]	7460
		β-Linalool[43]	6549
		p-Mentha-1,5-dien-8-ol[43]	519323
		Bornyl acetic ether[43]	6448
		Bicyclo[7.2.0]undec-4-ene, 4,11,11-trimethyl-8-methylene-, [1R-(1R*,4E,9S*)][43]	5281515
		2-Norpinene, 2,6-dimethyl-6-(4-methyl-3-pentenyl)-[43]	86608
		beta.-Sesquiphellandrene[43]	12315492
		1H-Benzocycloheptene, 2,4a,5,6,7,8-hexahydro-3,5,5,9-tetramethyl-, (R)-[43]	11586487
		δ-Cadinene[43]	441005
		Germacrene D[43]	5317570
		Trans-Caryophyllene[43]	5354499
		1,2,3,4,5-Pentamethylcyclopentadiene[43]	77667

		β-Bisabolene[43]	10104370
		Tricyclo[7.2.0.0(3,8)]undec-4-ene, 4,8,11,11-tetramethyl[43]	571934
		5-Isopropyl-2-methylbicyclo[3.1.0]hex-3-en[43]	561870
		Cyclohexene, 4-(1,5-dimethyl-1,4-hexadienyl)-1-methyl[43]	5352653
		Androstan-17-one, 3-ethyl-3-hydroxy-, (5.alpha.)[43]	14681481
		(6E)-3,7,11-Trimethyl-1,6,10-dodecatrien-3-ol[43]	5284507
		Andrographolide[43]	5318517
		(-)-Spathulenol[43]	13854255
		Caryophyllene oxide[43]	1742210
		α-Cedrol[43]	6432709
		Biphenylene, 1,2,3,6,7,8,8a,8b-octahydro-4,5-dimethyl[43]	583087
		Guaiol[43]	227829
		Torreyol[43]	11990360
		Isoledene[43]	530426
		α-Bisabolol[43]	10586
		4,6,6-Trimethyl-2-(3-methylbuta-1,3-dienyl)-3-oxatricyclo[5.1.0.0(2,4)]octane[43]	5369926
		9H-Cycloisolongifolene, 8-oxo[43]	600415
		Cyclodecacyclotetradecene, 14,15-didehydro-1,4,5,8,9,10,11,12,13,16,17,18,19,20-tetradecahydro[43]	5368333

		Bicyclo[4.4.0]dec-5-ene, 1,5-dimethyl-3-hydroxy-8-(1-methylene-2-hydroxyethyl-1)[43]	535386
		1,4,4,7a-Tetramethyl-2,4,5,6,7,7a-hexahydro-1H-indene-1,7-diol[43]	539247
		9-Oxoageraphorone[43]	73213927
		Stigmasterol[43]	5280794
		Octacosanoic acid[43]	10470
		Hydroxycinnamic acid[43]	637542
		Ferulic acid[43]	445858
		Caffeic acid[43]	689043
		γ-curcumene[44]	12304273
		2- carene[44]	79044
		Kolavenol[44]	6442554
		β-sitosterol[44]	222284
		Daucosterol[44]	5742590
		Succinic anhydride[44]	7922
		Encecalin[45]	114703
		4-hydroxybenzoic acid[45]	135
		Emodin[45]	3220
		Epifriedelanol[45]	119242
		n-dotriacontane[46]	11008
		Taraxasteryl acetate[46]	99738483
		2-Pentanone[46]	7895
		Amorph-4-en-7-ol[46]	91747339

		3-acetoxyamorpha-4, 7 (11)-dien-8-one[46]	91747336
		α-cadinol[46]	10398656
		Amorph-4, 7 (11)-dien-8-one[46]	102296360
		1-naphthalenol[46]	7005
		γ-cadinene[46]	92313
		γ-muurolene[46]	12313020
		β-cadinene[46]	10657
		α-terpinene[46]	7462
		b-farnesene[47]	5281517
17	*Agrimonia pilosa* var. *nepalensis* (D. Don) Nakai	Quercitrin[48]	5280459
		Hyperoside[48]	5281643
		Corosolic acid[48]	6918774
		Vitexin[48]	5280441
		Rutin[48]	5280805
		Luteolin-7- O-β-D-glucopyranoside[48]	5280637
		Quercetin[48]	5280343
		Tiliroside[48]	5320686
		Luteolin[48]	5280445
		Apigenin[48]	5280443
		Kaempferol[48]	5280863
		Tormentic acid[48]	73193
		Oleanolic acid[48]	10494
		Ursolic acid[48]	64945

		Luteolin glucuronide[49]	5280601
		Apigenin glucuronide[49]	5387370
		Apigenin 7-O-glucuronide[49]	5319484
		Agrimonolide[50]	15558543
		Agrimophol[50]	442901
		Ellagic acid[50]	5281855
18	*Ainsliaea pertyoides* Franch.	Friedelin[51]	91472
		Friedelinol[51]	146157554
		Isointermedeol[51]	527217
		Hexadecanoic acid[51]	985
		Stigmasterol[51]	5280794
		Acetyl ursolic acid[51]	619164
		Betulinic acid[51]	64971
		Macrophyllilactone F[52]	12041560
		Alantolactone[52]	72724
		Isoalantolactone[52]	73285
		11,13-dihydroisoalantolactone[52]	6451323
		Estafiatone[52]	101238032
		Dihydroestafiatone[52]	101238033
		β-Elemene[53]	6918391
		γ-elemene[53]	6432312

19	*Ainsliaea spicata* Vaniot	Zaluzanin C[54]	72646
		Estafiatone[54]	101238032
		Dihydroestafiatone[54]	101238033
		Isoamberboin[54]	14589063
		Dehydrocostuslactone[54]	73174
		Isolipidiol[54]	14589530
		epi-guaidiol A[54]	71719409
		Aphanamol I[54]	11031884
		Aphanamol II[54]	44566761
		10-hydroxy-6,10-epoxy-7(14)-isodaucane[54]	91750030
		Cyperusol C[54]	11230158
		α-dictyopterol[54]	23426951
		Kobusone[54]	6710676
		(–)-clovane 2,9-diol[54]	10681421
		Caryolane-1,9β-diol[54]	382536
		Pubescone[54]	102054514
		Japonicone A[54]	44571341
		1-o-acetyl-6-o-isobutyrylbritannilactone[54]	75528892
		(–)-α-cadinol[54]	6431302
		t-Cadinol[54]	160799

		15-oxo-T-cadinol[54]	101381938
20	*Akebia quinata* (Houtt.) Decne.	Lactic acid[55]	612
		Citric acid[55]	311
		Malic acid[55]	525
		Succinic acid[55]	1110
		Oleic acid[55]	445639
		Palmitic acid[55]	985
		Linoleic acid[55]	5280450
		Kalopanaxsaponin A[56]	73296
		Oleanolic acid[56]	10494
		Hederagenin[56]	73299
		L-ascorbic acid[57]	54670067
		Chlorogenic acid[58]	1794427
		Isochlorogenic acid A[58]	6474310
		Isochlorogenic acid C[58]	6474309
		Triterpenoid[58]	451674
21	*Alnus nepalensis*	Taraxerone[59]	92785
		Taraxerol[59]	92097
		Betulin[59]	72326
		Betulinic acid[59]	64971
		Lupeol[59]	259846
		β-sitosterol[59]	222284

		(−)-centrolobol[60]	11771038
		Oregonin[60]	14707658
		Platyphylloside[60]	9826264
		Hirsutenone[60]	637394
		Gallic acid[60]	370
		Quercetin[60]	5280343
		Quercetin-3-O-glucoside[60]	5280804
		Quercitrin[60]	5280459
		Quercetin-3-O-galactoside[60]	5281643
		Mangiferonic acid[60]	14034474
		Taraxeryl acetate[60]	5205968
		β-rosasterol[60]	241573
		Stigmasterol[60]	5280794
		Vanillin[60]	1183
		Physcion[60]	10639
		Platyphyllenone[61]	23786382
		Hirsutanonol[61]	9928190
		1-Nonacosanol[62]	243696
		Heptacosanoic acid[62]	23524
22	*Alstonia scholaris* (Linn.) R. Br.	Echitamine[63]	5364099
		α-amyrin acetate[63]	293754

		Ursolic acid[63]	64945
		Linalool[63]	6549
		Trans-linalool oxide[63]	6432254
		Cis-linalool oxide[63]	6428573
		α-terpineol[63]	17100
		2-phenylethyl acetate[63]	7654
		Terpinen-4-ol[63]	11230
		n-hexacosane[63]	12407
		Lupeol[63]	259846
		Palmitic acid[63]	985s
		β-amyrin[63]	73145
		Picrinine[63]	5320580
		Strictamine[63]	21159178
		β-sitosterol[63]	222284
		Akuammiginone[63]	101352946
		Echitaminic acid[63]	101352948
		Echitamidine N-oxide[63]	101678912
		Akuammicine N-oxide[63]	101306909
		Isoboonein[63]	10899112
		Alyxialactone[63]	14194343
		Loganin[63]	87691
		17-O-acetylechitamine[63]	101589338
		Scholarein A[63]	71523918

		Scholarein B[63]	102034145
		Scholarein C[63]	71523872
		Scholarein D[63]	71523873
		Scholarisine II[63]	49850933
		Akuammidine-N-oxide[63]	102423744
		Akuammidine[63]	15558574
		Kaempferol[63]	5280863
		Quercetin[63]	5280343
		Isorhamnetin[63]	5281654
		Quercetin-3-O-β-d-galactopyranoside[63]	5281643
		Lagunamine[63]	14706138
		Losbanine[63]	101589339
		Tubotaiwine[63]	13783720
		6,7-seco-angustilobine B[63]	13891912
		19-epischolaricine[63]	101587171
		N-methylscholaricine[63]	101587173
		N-methylburnamine[63]	101587172
		Vallesamine N-oxide[63]	71307282
		Alstonic acid A[63]	91895416
		Alstonic acid B[63]	91895417
		Scholarisine A[63]	101840191

		Picralinal[63]	46229103
		5-Methoxystrictamine[63]	102004590
		Cycloeucalenol[63]	101690
		Squalene[63]	638072
		α-Tocopherolquinone[63]	2734086
		Bis(2-ethylhexyl) phthalate[63]	8343
		Dibutyl phthalate[63]	3026
		E-Alstoscholarine[63]	16215339
		Z-Alstoscholarine[63]	16215340
		Manilamine[63]	101741721
		(E)-vallesamine[63]	146158636
		Nareline Ethyl Ether[63]	101712321
		5-Epi-Nareline Ethyl Ether[63]	101712322
		Nareline Methyl Ether[63]	101712320
		Scholaricine[63]	50900051
		Rhazimanine[63]	14109838
		19,20-E-vallesamine[63]	13783714
		Echitamidine[63]	10991442
		Nareline[63]	6443592
		ψ-akuammigine[63]	119079925

		Akuammicine[63]	10314057
		N-demethylechitamine[63]	91885202
		Oleic acid[63]	445639
		Linoleic acid[63]	5280450
		Stearic acid[63]	5281
		Alschomine[64]	11969856
		N1-Methoxymethyl Picrinine[64]	102004419
		Lupeol acetate[64]	92157
		Yohimbine[64]	8969
		Alstonidine[65]	12305773
		Alstonine[65]	441979
		Corialstonidine[65]	101656124
		Villalstonine[65]	5476353
		Pleiocarpamine[65]	5385014
		Macrocarpamine[65]	5472477
		Alpha-Amyrin Linoleate[65]	9987141
		Lupeol palmitate[65]	161739
		Chlorogenic acid[65]	1794427
		Alstovenine[65]	199742
		Reserpine[65]	5770
		Venenatine[65]	73061

		Macralstonine[65]	59052302
		Echitin[65]	101865246
		Porphyrine[65]	66868
		Corialstonine[65]	6443266
		Vallesamine[65]	101286269
		Tetrahydroalstonine[66]	72340
		Caoutchouc[66]	6557
		Akuammigine[66]	1268096
		Betulin[66]	72326
		Picraline[66]	131636670
		Angustilobine B[66]	13891905
		Erythrodiol[67]	101761
		Uvaol[67]	92802
		Oleanolic acid[67]	10494
		β-amyrin acetate[67]	92156
		Stigmasterol[67]	5280794
		Chlorophyll a[67]	12085802
		2-dodecyloxirane[67]	18604
		1,2-dimethoxy-4-(2-propenyl)benzene[67]	7127

		1,54- dibromotetrapentacontane[67]	545963
		2,6,10,15-tetramethylheptadecane[67]	41209
		Tritetracontane[67]	522398
		9-methyl-5- methylene-8-decen-2-one[67]	549791
		α-amyrin[67]	73170
		Cycloartanol[67]	12760132
23	*Amorphophallus konjac* K. Koch	Glucomannan[68]	24892726
		Trigonelline[68]	5570
		β-carotene[68]	5280489
		Choline[68]	305
		Niacin[68]	938
		Riboflavin[68]	493570
		Thiamine[68]	1130
		Serotonin[68]	5202
		Trans-N-(p-coumaroyl) serotonin[68]	5458879
		Saponin[68]	198016
24	*Amygdalus davidiana* (Carrière) de Vos ex Henry	Prupersin A[69]	102227208
		Amygdalin[69]	656516
		1-O-vanilloyl-beta-D-glucose[69]	14132344
		Vanilloloside[69]	44577222
		Androsin[69]	164648

		Prunasin[69]	119033
		Sambunigrin[69]	91434
		Naringenin[70]	439246
		Kaempferol[70]	5280863
		Dihydrokaempferol[70]	662
		β-Sitosterol glucoside[70]	5742590
		D-catechin[70]	9064
		Mumenin[70]	44257988
		Populnin[70]	10095180
		Prunin[70]	92794
		Quercimeritrin[70]	5282160
		Hesperetin-5-O-glucoside[70]	18625123
25	*Anemone rivularis* Buch.- Ham.	Acetophenone[71]	7410
		3-ethyl-2-methyl-hexane[71]	86067
		5,6-dimethyl-decane[71]	519255
		Benzaldehyde[71]	240
		Benzyl Alcohol[71]	244
		Heptanoic acid[71]	8094
		Benzeneacetaldehyde[71]	998
		Heptane,2,3-dimethyl-[71]	26375
		3-BHA[71]	8456

		Myristicin[71]	4276
		Undecane,5-methyl-[71]	94213
		Octane,4,5-diethyl-[71]	519254
		2,4-Dodecadienal[71]	5367530
		Undecane,2,4-dimethyl[71]	28476
		Patchouli alcohol[71]	10955174
		3-(6,6-Dimethyl-5-oxohept-2-enyl)-cyclohexanone[71]	5364977
		Caryophyllene[71]	5281515
		Anemoside B[72]	11636713
		Huzhangoside B[72]	49799269
		Huzhangoside D[72]	49799270
		Prosapogenin CP6[72]	15625347
		Ciwujianoside C3[72]	21626481
		Akebia Saponin D[72]	14284436
		Cauloside D[72]	21630094
		HN saponin H[72]	171596
		HN saponin F[72]	14101171
		Ursolic acid[72]	64945

		Betulinic acid[72]	64971
		β-sitosterol[72]	222284
		Oleanoic acid[72]	485707
		Ergosterol peroxide[72]	5351516
26	*Anisodus acutangulus* C. Y. Wu et C. Chen ex C. Chen et C. L. Chen	Anisodamine[73]	6918612
		Hyoscyamine[73]	154417
		Anisodine[73]	4105431
		Scopolamine[73]	11968014
27	*Ardisia crenata* Sims	Ardisiacrispin A[74]	10328746
		Ardisiacrispin B[74]	10441164
		Ardisicrenoside B[74]	10373894
		Ardisicrenoside A[74]	10260582
		Ardisicrenoside H[74]	100987880
		Ardisicrenoside G[74]	70689117
		Bergenin[75]	66065
		Friedelin[75]	91472
		Rapanone[75]	100659
		β-sitosterol[75]	222284
		Ardicrenin[75]	3083371
		Ardisiacrenoside I[76]	52918164

		Ardisicrenoside D[76]	101672539
		Primulanin[76]	44419565
		Cyclaminorin[76]	44566606
		11-O-galloylbergenin[77]	56680102
		11-O-syringylbergenin[77]	195481
		Embelin[77]	3218
28	*Ardisia japonica* (Thunb.) Bl.	Ardisianoside A[78]	16109779
		Ardisianoside B[78]	16109773
		Ardisianoside C[78]	16109780
		Ardisianoside D[78]	16109774
		Ardisianoside E[78]	16109781
		Ardisianoside F[78]	16109775
		Ardisianoside G[78]	16109776
		Ardisianoside H[78]	16109782
		Ardisianoside I[78]	16109777
		Ardisianoside J[78]	16109783
		Ardisianoside K[78]	16109778
		Ardisicrenoside A[78]	10260582
		Cyclamin[78]	441916
		Ardisiacrispin B[78]	10441164

		Primulanin[78]	44419565
		Ardisiamamilloside H[78]	11125847
		Ardisiamamilloside F[78]	44419566
		Ardisiamamillosede C[78]	44419578
		Ardisicrenoside G[78]	70689117
		Bergenin[79]	66065
		Afzelin[79]	5316673
		Quercitrin[79]	5280459
		Bauerenol[79]	111220
		Bauerenone[79]	101289675
		α-spinasterol[79]	5281331
		Ardisin[77]	417291
		Ardisinol II[77]	6454482
		Embelin[77]	3218
		Ilexol[77]	287684
		Myricitrin[77]	5281673
		Quercetin[77]	5280343
		Rapanone[77]	100659
		Maesanin[77]	5384838

		Cyclamiretin A[80]	12305336
		Tri-O-methylnorbergenin[81]	11360257
		Norbergenin[81]	73192
29	*Areca catechu* L.	Arecoline[82]	2230
		Arecaidine[82]	10355
		Arecolidine[82]	5319882
		Ethyl N-methyl-l,2,5,6-tetrahydro-pyridine-3-carboxylate[82]	34167
		Methyl Nicotinate[82]	7151
		Ethyl Nicotinate[82]	69188
		Ethyl N-methylpiperidine-3-carboxylate[82]	97981
		Nicotine[82]	89594
		Isoguvacine[82]	3765
		Isorhamnetin[82]	5281654
		Chrysoeriol[82]	5280666
		Luteolin[82]	5280445
		Quercetin[82]	5280343
		Liquiritigenin[82]	114829
		Jacareubin[82]	5281644

		Procyanidin A1[82]	5089889
		Procyanidin B1[82]	11250133
		Procyanidin B2[82]	122738
		Arecatannin B1[82]	14237657
		Arecatannin C1[82]	9876038
		Arecatannin A3[82]	16162334
		Arecatannin B2[82]	71448962
		Ursonic acid[82]	9890209
		Arborinol[82]	12305177
		Arborinol methyl ether[82]	101600057
		Fernenol[82]	12305178
		Arundoin[82]	12308619
		Cycloartenol[82]	92110
		β-sitosterol[82]	222284
		Lauric acid[82]	3893
		Myristic acid[82]	11005
		Palmitic acid[82]	985
		Stearic acid[82]	5281
		Oleic acid[82]	445639
		Chrysophanol[82]	10208

		Physcion[82]	10639
		P-hydroxybenzoic acid[82]	135
		Epoxyconiferyl alcohol[82]	57403796
		Protocatechuic acid[82]	72
		Isovanillic acid[82]	12575
		Resveratrol[82]	445154
		Ferulic acid[82]	445858
		Vanillic acid[82]	8468
		De-O-methyllasiodiplodin[82]	14562693
		Cyclo-(Leu-Tyr)[82]	15550385
		Gallic acid[83]	370
		Epicatechin[83]	72276
		Rutin[83]	5280805
		Naringin[83]	442428
		Ursolic acid[83]	64945
		Ursolic acid acetate[83]	15917992
		Cylindrin[83]	189045
		Ergosterol peroxide[83]	5351516

		B-sitostenone[83]	60123241
		Linoleic acid[83]	5280450
		α-lipoic acid[83]	6112
		Decanoic acid[83]	2969
		Guvacoline[83]	160492
		Guvacine[83]	3532
		2,6-Di-t-butyl-4-methylphenol[83]	31404
		Methyl cinnamate[83]	637520
		Nonanoic acid[83]	8158
		2,4-Di-t-butylphenol[83]	7311
		Benzoic acid[83]	243
		Pentadecanoic acid[83]	13849
		Catechin[84]	9064
		Leucocyanidin[84]	71629
		Dodecenoic acid[84]	96204
		Tetradecenoic acid[84]	162384
		Hexadecenoic acid[84]	5282743
		Choline[85]	305

		Trimyristin[85]	11148
30	*Aristolochia cucurbitoides* C.F. Liang	(+/-)-Licarin A[86]	5281836
		(-)-Licarin-B[86]	10860310
		Eupomatenoid-7[86]	10314175
		Aristolochic acid[87]	2236
31	*Aristolochia debilis* Sieb. et Zucc	(3R,4S,5R,7R,10R)-3,4-epoxy-11-hydroxy-1-pseudoguaiene[88]	23642712
		Aristolochic acid I[88]	2236
		β-sitosterol[88]	222284
		Daucosterol[88]	296119
		2',3' -dihydroxypropyl pentadecanoate[88]	190750
		β-Ocimene[89]	18756
		Myrcene[89]	31253
		Limonene[89]	22311
		1,8-Cineole[89]	2758
		Borneol[89]	64685
		Bornyl acetate[89]	6448
		Camphor[89]	2537
		Camphene[89]	6616
		α-Pinene[89]	6654
		β-Pinene[89]	14896

		β-Elemene[89]	6918391
		β-Caryophyllene[89]	5281515
		Calarene[89]	28481
		1(10)-Aristolen-13-al[89]	10398499
		γ-Cadinene[89]	6432404
		δ-Cadinene[89]	441005
		Calamenene[89]	6429077
		α-Guaiene[89]	5317844
		δ-Guaiene[89]	94275
		α-Cubebene[89]	86609
		α-Santalene[89]	94164
		β-Chamigrene[89]	442353
		Aristolochic acid C[90]	165274
		Aristolochic acid IVa[90]	161218
		Aristolochic acid II[90]	108168
		Aristolochic acid IV[90]	167493
		Ariskanin B[90]	10315388
		Ariskanin A[90]	5320068
		Magnoflorine[91]	73337
		Aristolactam I[91]	96710

32	*Aristolochia transsecta* (Chatterjee) C. Y. Wu ex S. M. Hwang	Aristolochic acid Va[92]	1941
		Aristolochic acid IVa[92]	161218
		Aristololactam II[92]	148745
		Aristolochic acid II[92]	108168
		Aristololactam I[92]	96710
		Aristolochic acid I[92]	2236

References

1. Lai, X., Liang, H., Zhao, Y. & Wang, B. Simultaneous determination of seven active flavonols in the flowers of Abelmoschus manihot by HPLC. *J. Chromatogr. Sci.* **47**, 206–210 (2009).

2. Venkatrao Mohite, A. & Vithoba Gurav, R. Phytochemical and Nutritional Studies in the Genus Abelmoschus Medik . *Bioact. Compd. [Working Title]* 1–13 (2020) doi:10.5772/intechopen.93019.

3. Ali, S. I., Gopalakrishnan, B. & Venkatesalu, V. Pharmacognosy, Phytochemistry and Pharmacological Properties of Achillea millefolium L.: A Review. *Phyther. Res.* **31**, 1140–1161 (2017).

4. Lakshmi, T., Geetha, R. V., Roy, A. & Aravind Kumar, S. Yarrow (Achillea millefolium Linn.) a herbal medicinal plant with broad therapeutic use - A review. *Int. J. Pharm. Sci. Rev. Res.* **9**, 136–141 (2011).

5. Bais, S. Review on Phytochemical and Pharmacological Activity of Yarrow (Achilla millefolium Linn). *Der Pharma Chem.* **9**, 89–96 (2017).

6. Singh, N. & Thakur, R. A Review on Pharmacological aspects of Tagetes erecta Linn. *PharmaTutor* (2019) doi:10.23880/ipcm-16000188.

7. Sinan, K. I. *et al.* Qualitative phytochemical fingerprint and network pharmacology investigation of achyranthes aspera linn. extracts. *Molecules* **25**, (2020).

8. Dey, A. Achyranthes aspera L: Phytochemical and pharmacological aspects. *Int. J. Pharm. Sci. Rev. Res.* **9**, 72–82 (2011).

9. Yogeshwari, C. & Kalaichelvi, K. Comparitive phytochemical screening of Acmella calva (dc.) r. k. Jansen and crotalaria ovalifolia wall: Potential medicinal herbs. *J. Med. Plants Stud. JMPS* **277**, 277–279 (2017).

10. Moin, S., Shibu, S., Wesley, S. & B, C. D. A c a d e m i c S c i e n c e s. **4**, (2012).

11. PHYTOCHEMICAL CONSTITUENT OF ACONITUM S PECIES-A REVIEW Gajalakshmi S , Jeyanthi P , Vijayalakshmi S , Devi Rajeswari V * School of Biosciences and Technology , VIT University ISSN 0976-4550 Medicinal uses Table1 : List of various species of Aconitum and . 121–127 (2011).

12. Nyirimigabo, E. *et al.* A review on phytochemistry, pharmacology and toxicology studies of Aconitum. *J. Pharm. Pharmacol.* **67**, 1–19 (2015).

13. Li, Y., Meng, Y., Shen, S. & Wang, Y. Karyological studies of aconitum brachypodum and related species. *Cytologia (Tokyo).* **77**, 491–498 (2012).

14. Kilmer, P. D. Review Article: Review Article. *Journal. Theory, Pract. Crit.* **11**, 369–373 (2010).

15. Liu, X. X. *et al.* Cardioactive C 19-diterpenoid alkaloids from the lateral roots of Aconitum carmichaeli 'Fu Zi'. *Chem. Pharm. Bull.* **60**, 144–149 (2012).

16. Xiong, L. *et al.* Alkaloids isolated from the lateral root of Aconitum carmichaelii. *Molecules* **17**, 9939–9946 (2012).

17. Yu, M., Yang, Y. X., Shu, X. Y., Huang, J. & Hou, D. Bin. Aconitum carmichaelii Debeaux, cultivated as a medicinal plant in western China. *Genet. Resour. Crop Evol.* **63**, 919–924 (2016).

18. Singhuber, J., Zhu, M., Prinz, S. & Kopp, B. Aconitum in Traditional Chinese Medicine-A valuable drug or an unpredictable risk? *J. Ethnopharmacol.* **126**, 18–30 (2009).

19. Chandra, D. & Prasad, K. Phytochemicals of Acorus calamus (Sweet flag). (2017).

20. Joshi, R. K. Acorus calamus Linn.: phytoconstituents and bactericidal property. *World J. Microbiol. Biotechnol.* **32**, (2016).

21. Kim, K. H. *et al.* A new aliphatic alcohol and cytotoxic chemical constituents from Acorus gramineus rhizomes. *Biosci. Biotechnol. Biochem.* **79**, 1402–1405 (2015).

22. Kim, K. H. *et al.* Alkaloids from. **26**, 3–8 (2015).

23. Moon, J. M., Sung, H. M., Jung, H. J., Seo, J. W. & Wee, J. H. In vivo evaluation of hot water extract of Acorus gramineus root against benign prostatic hyperplasia. *BMC Complement. Altern. Med.* **17**, 1–10 (2017).

24. Mezzocannone, V. & Index-acorus, W. Allelochemical Activity of Phenylpropanes. **28**, 2319–2321 (1989).

25. Kim, K. H. *et al.* Phenolic constituents from the rhizomes of Acorus gramineus and their biological evaluation on antitumor and anti-inflammatory activities. *Bioorganic Med. Chem. Lett.* **22**, 6155–6159 (2012).

26. Perrett, S. & Whitfield, P. J. Anthelmintic and Pesticidal Activity of Acorus gramineus (Araceae) is Associated with Phenylpropanoid Asarones. **9**, 405–409 (1995).

27. Pan, C. *et al.* Phytochemical constituents and pharmacological activities of plants from the genus Adiantum: A review. *Trop. J. Pharm. Res.* **10**, 681–692 (2011).

28. Li, Y. Y. *et al.* Molecules and functions of Aesculus chinensis Bunge Bark volatiles. *Emirates J. Food Agric.* **30**, 809–819 (2018).

29. Chen, J. *et al.* Determination of four major saponins in the seeds of Aesculus chinensis Bunge using accelerated solvent extraction followed by high-performance liquid chromatography and electrospray-time of flight mass spectrometry. *Anal. Chim. Acta* **596**, 273–280 (2007).

30. Alongkornsopit, J., Wipasa, J., Luangkamin, S. & Wongkham, W. Anticancer activity of ethyl

acetate and n-butanol extracts from rhizomes of Agapetes megacarpa W.W. Smith. *African J. Biotechnol.* **10**, 3455–3462 (2011).

31. Address, E. -amyrin และ 3. 5–8.

32. Мещерякова, Л. М. & Понтак, Л. С. химияNo Title.

33. Dũng, N. X. *et al.* Constituents of the leaf and flower oils of agastache rugosa (fisch. et mey) o. Kuntze from Vietnam. *J. Essent. Oil Res.* **8**, 135–138 (1996).

34. Li, H. Q., Liu, Q. Z., Liu, Z. L., Du, S. S. & Deng, Z. W. Chemical composition and nematicidal activity of essential oil of agastache rugosa against meloidogyne incognita. *Molecules* **18**, 4170–4180 (2013).

35. Cao, P. *et al.* Chemical constituents and coagulation activity of Agastache rugosa. *BMC Complement. Altern. Med.* **17**, 1–8 (2017).

36. Lee, C., Kim, H. & Kho, Y. Agastinol and agastenol, novel lignans from Agastache rugosa and their evaluation in an apoptosis inhibition assay. *J. Nat. Prod.* **65**, 414–416 (2002).

37. Zielińska, S. & Matkowski, A. Phytochemistry and bioactivity of aromatic and medicinal plants from the genus Agastache (Lamiaceae). *Phytochem. Rev.* **13**, 391–416 (2014).

38. Debnath, M., Pandey, M., Sharma, R., Thakur, G. S. & Lal, P. Biotechnological intervention of Agave sisalana: A unique fiber yielding plant with medicinal property. *J. Med. Plants Res.* **4**, 177–187 (2010).

39. Tewari, D., Tripathi, Y. C. & Anjum, N. Agave Sislana: a Plant With High Chemical Diversity and Medicinal Importance. *world J. Pharm. Res.* **3**, 238–249 (2014).

40. Sharma, S. & Varshney, V. K. Chemical Analysis of Agave Sisalana Juice for Its. *Acta Chim. Pharm. Indica* **2**, 60–66 (2012).

41. Santos, J. D. G., Vieira, I. J. C., Braz-Filho, R. & Branco, A. Chemicals from agave sisalana biomass: Isolation and identification. *Int. J. Mol. Sci.* **16**, 8761–8771 (2015).

42. Chen, P. Y. *et al.* Cytotoxic steroidal saponins from Agave sisalana. *Planta Med.* **77**, 929–933 (2011).

43. Pradesh, U. No Title. **11**, 2510–2520 (2020).

44. King, S. R. M. *et al.* Evaluation of Wound Healing Activity of Ageratina. **5**, 1873–1876 (2017).

45. Zheng, G. *et al.* Phytochemistry Specialized metabolites from Ageratina adenophora and their inhibitory activities against pathogenic fungi. *Phytochemistry* **148**, 57–62 (2018).

46. Tripathi, Y. C., Saini, N., Anjum, N. & Verma, P. K. A Review of Ethnomedicinal , Phytochemical , Pharmacological and Toxicological Aspects of Eupatorium adenophorum Spreng. **8**, 25–35 (2018).

47. Subba, B. & Kandel, R. C. Chemical Composition and Bioactivity of Essential Oil of Ageratina adenophora Chemical Composition and Bioactivity of Essential Oil of Ageratina adenophora from Bhaktapur District of Nepal. (2014) doi:10.3126/jncs.v30i0.9350.

48. Liu, X. *et al.* Glucosidase inhibitory activity and antioxidant activity of flavonoid compound and triterpenoid compound from Agrimonia Pilosa Ledeb. *BMC Complement. Altern. Med.* **14**, (2014).

49. Kubínová, R., Švajdlenka, E. & Jankovská, D. Anticholinesterase, antioxidant activity and phytochemical investigation into aqueous extracts from five species of Agrimonia genus. *Nat. Prod. Res.* **30**, 1174–1177 (2016).

50. Kunwar, R. M., Chundamani Burlakoti, •, Chhote, •, Chowdhary, L. & Bussmann, R. W. Medicinal and Aromatic Plant Science and Biotechnology Medicinal Plants in Farwest Nepal: Indigenous Uses and Pharmacological Validity. (2010).

51. Li, X., Wang, W., Jaeger, F. & Kreyenschmidt, J. Ainsliaolide D: A new sesquiterpene lactone from Ainsliaea pertyoides. *Nat. Prod. Res.* **28**, 115–118 (2014).

52. Shi, Z. R. *et al.* Structurally novel C17-sesquiterpene lactones from Ainsliaea pertyoides. *RSC Adv.* **5**, 91640–91644 (2015).

53. Wang, S. A review of medicinal plant species with elemene in China. *African J. Pharm. Pharmacol.* **6**, 3032–3040 (2012).

54. Shi, Z. R. *et al.* Sesquiterpenoids from Ainsliaea spicata and their cytotoxic and NO production inhibitory activities. *Phytochem. Lett.* **18**, 87–94 (2016).

55. Li, L., Yao, X., Zhong, C., Chen, X. & Huang, H. Akebia: A potential new fruit crop in China. *HortScience* **45**, 4–10 (2010).

56. Choi, J., Jung, H., Lee, K. & Park, H. Obtained from the Stem of Akebia quinata. **8**, 78–85 (2005).

57. Ochmian, I., Kubus, M. & Guan, T. Charakterystyka i ocena właściwości chemicznych owoców akebii pięciolistkowej akebia quinata (houtt.) decne i palecznika chińskiego (p. fargesa) decaisnea insignis (griff.) hokk. f. and thomson uprawianych w szczecinie i w arboretum w glinnej (północno-za. *J. Elem.* **19**, 1073–1084 (2014).

58. Shin, S. *et al.* Ameliorating effect of Akebia quinata fruit extracts on skin aging induced b advanced glycation end products. *Nutrients* **7**, 9337–9352 (2015).

59. Sati, S. C., Sati, N. & Sati, O. P. Bioactive constituents and medicinal importance of genus Alnus. *Pharmacogn. Rev.* **5**, 174–183 (2011).

60. Ren, X. *et al.* The genus Alnus, a comprehensive outline of its chemical constituents and biological activities. *Molecules* **22**, (2017).

61. Yadav, D. *et al.* Antifilarial diarylheptanoids from Alnus nepalensis leaves growing in high altitude areas of Uttarakhand, India. *Phytomedicine* **20**, 124–132 (2013).

62. Phan, M. G., Chinh Truong, T. T., Phan, T. S., Matsunami, K. & Otsuka, H. Mangiferonic acid, 22-hydroxyhopan-3-one, and physcion as specific chemical markers for Alnus nepalensis. *Biochem. Syst. Ecol.* **38**, 1065–1068 (2010).

63. Kaushik, D., Rana, A., Kaushik, P. & Sharma, N. Alstonia scholaris: It's Phytochemistry and pharmacology. *Chronicles Young Sci.* **2**, 71 (2011).

64. Dey, A. Alstonia scholaris R.Br. (Apocynaceae): Phytochemistry and pharmacology: A concise review. *J. Appl. Pharm. Sci.* **1**, 51–57 (2011).

65. Baliga, M. S. Alstonia scholaris Linn R Br in the treatment and prevention of cancer: Past, present, and future. *Integr. Cancer Ther.* **9**, 261–269 (2010).

66. Baliga, M. S. Review of the phytochemical, pharmacological and toxicological properties of Alstonia Scholaris Linn. R. Br (Saptaparna). *Chin. J. Integr. Med.* (2012) doi:10.1007/s11655-011-0947-0.

67. Ragasa, C. Y., Batarra, T. C., Tan, M. C. S. & van Altena, I. A. Chemical constituents of Alstonia scholaris (L.) R. Br. *Der Pharma Chem.* **8**, 193–196 (2016).

68. Chua, M., Baldwin, T. C., Hocking, T. J. & Chan, K. Traditional uses and potential health benefits of Amorphophallus konjac K. Koch ex N.E.Br. *J. Ethnopharmacol.* **128**, 268–278 (2010).

69. Chen, X. Y. *et al.* Aromatic glucosides from the seeds of Prunus davidiana. *J. Nat. Prod.* **76**, 1528–1534 (2013).

70. Choi, J. S., Woo, W. S., Young, H. S. & Park, J. H. Phytochemical study on Prunus davidiana. *Arch. Pharm. Res.* **13**, 374–378 (1990).

71. Baojun Shi. Chemical composition, antibacterial and antioxidant activity of the essential oil of Anemone rivularis. *J. Med. Plants Res.* **6**, 4221–4224 (2011).

72. Anh Minh, C. T. *et al.* A new saponin and other constituents from Anemone rivularis Buch.-Ham. *Biochem. Syst. Ecol.* **44**, 270–274 (2012).

73. Kai, G. *et al.* Enhancing the production of tropane alkaloids in transgenic Anisodus acutangulus hairy root cultures by over-expressing tropinone reductase i and hyoscyamine-6β-hydroxylase. *Mol. Biosyst.* **8**, 2883–2890 (2012).

74. Liu, D., Wang, N., Zhang, X. & Yao, X. Three New Triterpenoid Saponins from Ardisia crenata The roots of Ardisia crenata S ims are used as the traditional Chinese medicine Zhu Sha Gen for the treatment of respiratory tract infections and menstrual disorders in China [8]. We have reported sev. **94**, 693–702 (2011).

75. M, D., S, K. & K M, R. Phytochemical Screening of Ardisia Blatteri Gamble: an Endemic Plant of Southern Western Ghats, Tamil Nadu, India. *Int. Res. J. Pharm.* **7**, 31–35 (2016).

76. Zheng, Z. F., Xu, J. F., Feng, Z. M. & Zhang, P. C. Cytotoxic triterpenoid saponins from the roots of Ardisia crenata. *J. Asian Nat. Prod. Res.* **10**, 833–839 (2008).

77. Kobayashi, H. & De Mejía, E. The genus Ardisia: A novel source of health-promoting compounds and phytopharmaceuticals. *J. Ethnopharmacol.* **96**, 347–354 (2005).

78. Chang, X. *et al.* Biologically active triterpenoid saponins from Ardisia japonica. *J. Nat. Prod.* **70**, 179–187 (2007).

79. Yu, K. Y. *et al.* A new compound, methylbergenin along with eight known compounds with cytotoxicity and anti-inflammatory activity from Ardisia japonica. *Nat. Prod. Res.* **31**, 2581–2586 (2017).

80. Zhou, L. Characterization of Three New Triterpenoid. **56**, 1669–1675 (1993).

81. Piacente, S., Pizza, C., De Tommasi, N. & Mahmood, N. Constituents of Ardisia japonica and their in vitro anti-HIV activity. *J. Nat. Prod.* **59**, 565–569 (1996).

82. Peng, W. *et al.* Areca catechu L. (Arecaceae): A review of its traditional uses, botany, phytochemistry, pharmacology and toxicology. *J. Ethnopharmacol.* **164**, 340–356 (2015).

83. Xiao, Y., Yang, Y., Yong, J. & Lu, C. Chemical Components and Biological Activities of Areca. *Biomed. Res. Rev. Rev.* **3**, 1–4 (2019).

84. Station, R. & Nadu, T. Amudhan et al. ,. **3**, 4151–4157 (2012).

85. Rani, S., Rahman, K. & Idris, M. Ethnomedicinal , Pharmacological and Phytochemical Screening of Supari (Areca catechu Linn .): A Review. **8**, 6–20 (2018).

86. Enriquez, R. G. & Chavez, M. A. Phytochemical Investigations of Plants of the Genus. **47**, 896–899 (1984).

87. Christodoulakis, N. S., Kotsironi, K., Tsafantakis, N., Stefi, A. L. & Fokialakis, N. Leaf structure and phytochemical analysis of Aristolochia baetica, a traditionally used pharmaceutical plant. *J. Herbs, Spices Med. Plants* **25**, 88–103 (2019).

88. Wang, X. *et al.* A new sesquiterpene, a new monoterpene and other constituents with anti-inflammatory activities from the roots of Aristolochia debilis. *Nat. Prod. Res.* **34**, 351–358 (2020).

89. Wu, T. S., Damu, A. G., Su, C. R. & Kuo, P. C. Terpenoids of Aristolochia and their biological activities. *Nat. Prod. Rep.* **21**, 594–624 (2004).

90. Jou, J. H., Li, C. Y., Schelonka, E. P., Lin, C. H. & Wu, T. S. Analysis of the analogues of aristolochic acid and aristolactam in the plant of Aristolochia genus by HPLC. *J. Food Drug Anal.* **12**, 40–45 (2004).

91. Fan, Y., Li, Z. & Xi, J. Recent developments in detoxication techniques for aristolochic acid-containing traditional Chinese medicines. *RSC Adv.* **10**, 1410–1425 (2020).

92. Zhang, C. *et al.* Simultaneous determination of five aristolochic acids and two aristololactams in Aristolochia plants by high-performance liquid chromatography. *Biomed. Chromatogr.* **20**, 309–318 (2006).

Serial No.	Plant name	Chemical name	Pubchem CID
01	*Angelica sinensis* (Oliv.) Diels	Z-Ligustilide[1]	5319022
		Senkyunolide F[1]	11241196
		E-Ligustilide[1]	5877292
		3-Butylphthalide[1]	61361
		Senkyunolide G[1]	10013283
		Senkyunolide[1]	3085257
		Senkyunolide H[1]	13965088
		Senkyunolide J[1]	24121290
		Z-6-Hydroxy-7-methoxydihydroligustilide[1]	44575249
		6,7-Dihydroxyligustilide[1]	6433088
		Z-6,7-Epoxyligustilide[1]	5317139
		Brefeldin A[1]	5287620
		Z-Butylidenephthalide[1]	642376
		3-Butylidene-4-hydroxyphthalide[1]	642373
		3-Butylidene-7-hydroxyphthalide[1]	5281559
		10-Angeloylbutylphthalide[1]	11572826
		Riligustilide[1]	6442656
		Levistolide A[1]	70698035
		Angelicolide[1]	494308
		Angelicide[1]	5316848

		Tokinolide B[1]	11090206
		Ansaspirolide[1]	44575265
		Ferulic acid[1]	445858
		Cis-Ferulic acid[1]	1548883
		Caffeic acid[1]	689043
		E-Coniferin[1]	3496897
		Ferulic aldehyde[1]	5280536
		Isoeugenol[1]	853433
		Guaiacylglycerol[1]	14579
		3-O-Caffeoyl-D-quinic acid[1]	1794426
		Chlorogenic acid[1]	1794427
		Coniferyl ferulate[1]	6441913
		Angeliferulate[1]	11654315
		P-Hydroxyphenethyl trans-ferulate[1]	637308
		Magnolol[1]	72300
		Eleutheroside B1[1]	12302278
		Isoimperatorin[1]	68081
		Imperatorin[1]	10212
		Bergapten[1]	2355

		α-Pinene[1]	6654
		Verbenone[1]	29025
		Carvacrol[1]	10364
		Allo-Ocimene[1]	5368821
		Camphanic acid[1]	565594
		Myrcene[1]	31253
		β-Ocimene[1]	5281553
		β-Bisabolene[1]	10104370
		Acoradiene[1]	90351
		Trans-β-Farnesene[1]	5281517
		γ-Elemene[1]	6432312
		Cuparene[1]	86895
		β-Cedrene[1]	11106485
		Limonene[1]	22311
		Safranal[1]	61041
		Copaene[1]	12303902
		Eucarvone[1]	136330
		γ-Cadinene[1]	92313
		δ-Cadinene[1]	441005

		Octadecane[1]	11635
		6-Butyl-1,4-cycloheptadiene[1]	556470
		2-Methyldodecan-5-one[1]	5319599
		Phenol[1]	996
		N-Butylbenzenesulfonamide[1]	19241
		Ethylbenzene[1]	7500
		Acetophenone[1]	7410
		4-(2-Hydroxy-1-methoxyethyl)-phenol[1]	22297411
		Phthalic acid[1]	1017
		Bis (2-ethylhexyl) phthalate[1]	8343
		Dibutyl phthalate[1]	3026
		P-Cresol[1]	2879
		O-Cresol[1]	335
		2,3-Dimethylphenol[1]	10687
		P-Ethylphenol[1]	31242
		m-Ethylphenol[1]	12101
		4-Ethylresorcinol[1]	17927
		2,4-Dihydroxyacetophenone[1]	6990
		Guaiacol[1]	460

		p-Hydroxybenzoic acid[1]	135
		Protocatechuic acid[1]	72
		Vanillic acid[1]	8468
		Vanillin[1]	1183
		Anisic acid[1]	7478
		p-Ethylbenzaldehyde[1]	20861
		3,4-Dimethylbenzaldehyde[1]	22278
		2,4,6-Trimethylbenzaldehyde[1]	10254
		2,3,6-Trimethylbenzoic acid[1]	17314
		Folinic acid[1]	135403648
		Folic acid[1]	135398658
		Baicalin[1]	64982
		Hyperoside[1]	5281643
		2″-O-(2‴-Methylbutyryl)-isoswertisin[1]	21578034
		3R,8S-Falcarindiol[1]	5281148
		Oplopandiol[1]	6474833
		Harman[1]	5281404
		Flazine[1]	5377686

		Nicotinic acid[1]	938
		Choline[1]	305
		β-Sitosterol[1]	222284
		α-Spinasterol[1]	5281331
		Stigmasterol[1]	5280794
		Daucosterol[1]	296119
		Dodecan-1-ol[1]	8193
		Tetradecan-1-ol[1]	8209
		2-Ethyl-hexan-1-ol[1]	7720
		Linoleic acid[1]	5280450
		Linoleic acid ethyl ester[1]	5282184
		Myristic acid[1]	11005
		Palmitic acid[1]	985
		Hexadecanoic acid ethyl ester[1]	12366
		Lignoceric acid[1]	11197
		Butanedioic acid[1]	1110
		Azelaic acid[1]	2266
		Sebacic acid[1]	5192

		1,3-Dilinolenin[1]	45934043
		Adenine[1]	190
		Allantoin[1]	204
		Uracil[1]	1174
		Hypoxanthine-9-β-D-ribofuranoside[1]	135398641
		Apiole[2]	10659
		(Z)-β-Ocimene[2]	5320250
		Octanal[2]	454
		Pentyl benzene[2]	10864
		β-Caryophyllene[2]	5281515
		Aromadendrene[2]	91354
		β-Barbatene[2]	14109421
		(Z)-β-Farnesene[2]	5317319
		Muurola-4,11-diene[2]	6429206
		α-Terpineol[2]	17100
		β-Chamigrene[2]	442353
		2,3,6-Trimethylbenzaldehyde[2]	10236014
		Globulol[2]	12304985
		Rosifoliol[2]	527256

		Elemicine[2]	10248
		6-Undecanone[3]	13561
		2-Methoxy-4-vinylphenol[3]	332
		1,4-Cyclohexadiene-1,2-dicarboxylic anhydride[3]	138348
		Dehydroaromadendrene[3]	91746711
		Linoleic acid, methyl ester[3]	5284421
		Oleic acid, methyl ester[3]	5364509
		Mono(2-ethylhexyl) phthalate[3]	20393
		1,9-Dioxa-4,6-diazacycloundecane-5-thione[3]	5375180
		Butanal[4]	261
		3,5-dimethylbenzaldehyde[4]	34225
		Camphene[4]	6616
		α-cedrene[4]	6431015
		α-terpinolene[4]	11463
		Isobutanal[4]	6561
		4-hydroxy-3-butylphthalide[4]	11074544
		2-Methylbutanal[4]	7284
		3-Methylbutanal[4]	11552
		Tridecane[4]	12388
		Decanal[4]	8175

		Bergamiol[4]	8294
		5-Methylfurfural[4]	12097
		δ-Guaiene[4]	94275
		6-undecanol[4]	32045
		4-methyl-6-hepten-3-one[4]	117297
		3-carene[4]	26049
		4-octanone[4]	11516
		Decursin[4]	442126
		3,7-dimethylnonane[4]	28458
		2,4-dimethylbenzaldehyde[4]	61814
		Butanoic acid[4]	264
		2,4,6-trimethyloctane[4]	545612
02	*Arctium lappa* L	Diarctigenin[5]	16215736
		Arctiin[5]	100528
		Arctigenin[5]	64981
		3-benzyl-6-(1-hydroxyethyl)- 2,5-piperazinedione[5]	75012092
		3-benzyl-2,5- piperazinedione[5]	138409
		Arctignan A[5]	73425485
		Arctignan B[5]	73425486
		Arctignan C[5]	73425487

		Arctignan D[5]	73425493
		Arctignan E[5]	73425494
		Lappaol A[5]	323894
		Lappaol B[5]	46173977
		Isolappaol C[5]	16105447
		Lappaol C[5]	323896
		Lappaol D[5]	46174000
		Lappaol E[5]	73425454
		Lappaol F[5]	73425459
		Lappaol H[5]	24758070
		Neoarctin A[5]	46173974
		Neoarctin B[5]	46173975
		Matairesinoside[5]	486612
		Matairesinol[5]	119205
		Pinoresinol[5]	73399
		Styraxlignolide E[5]	11432381
		Styraxlignolide D[5]	11179934
		Syringaresinol[5]	100067
		Trachelogenin[5]	452855
		β-eudesmol[5]	91457
		Ursolic acid[5]	64945

		Oleanolic acid[5]	10494
		Arctiopicrin[5]	5281423
		Onopordopicrin[5]	6440861
		Dehydrovomifoliol[5]	688492
		Loliolide[5]	100332
		Dehydromelitensin[5]	10333020
		Melitensin[5]	14162547
		Baicalin[5]	64982
		Luteolin[5]	5280445
		Rutin[5]	5280805
		Quercitrin[5]	5280459
		Quercetin[5]	5280343
		Quercetin 3-O-glucuronide[5]	5274585
		Quercetin 3-vicianoside[5]	44259139
		Genestein[5]	5280961
		Nobiletin[5]	72344
		Tangeretin[5]	68077
		β-sitosterol[5]	222284
		Daucosterol[5]	5742590
		Methyl palmitate[5]	8181
		Methyl linoleate[5]	5284421

		Methyl linolenate[5]	5319706
		Methyl stearate[5]	8201
		Methyl oleate[5]	5364509
		Hexadecanoic acid[5]	985
		Linoleic acid[5]	5280450
		Linolenic acid[5]	5280934
		Stearic acid[5]	5281
		Oleic acid[5]	445639
		Arctinone-a[5]	71587386
		Arctinone-b[5]	10014817
		Arctinol-a[5]	13939273
		Arctinol-b[5]	46842526
		Arctinal[5]	13779265
		Arctic acid-b[5]	71587385
		Arctic acid-c[5]	71587384
		Methyl arctate-b[5]	71587387
		Dehydrocostus lactone[5]	73174
		Lappaphen-a[5]	13856336
		Lappaphen-b[5]	13856337
		Caffeic acid[5]	689043

		Caffeic acid 4-O-glucoside[5]	6148082
		Chlorogenic acid[5]	1794427
		p-coumaric acid[5]	637542
		Benzoic Acid[5]	243
		Cynarin[5]	205954
		1-O-caffeoylquinic acid[5]	131751066
		4-O-caffeoylquinic acid[5]	9798666
		3 5-O-caffeoylquinic acid[5]	5280633
		1,3-di-O-caffeoylquinic acid[5]	6474640
		1,3,5-tri-O-caffeoylquinic acid[5]	10190081
		3,4-dicaffeoylquinic acid[5]	5281780
		1,4-di-O-caffeoylquinic acid[5]	12358846
		3,5-di-O-caffeoylquinic acid[5]	13604687
		4,5-dicaffeoylquinic acid[5]	10324242
		3-feruloyl-5-caffeoylquinic acid[5]	101710864
		3,4,5-tricaffeoylquinic acid[5]	6440783
		5-hydroxymaltol[5]	70627
		Succinic acid[5]	1110

		Arabinogalactan[5]	24847856
		Galacturonic acid[5]	439215
		Raffinose[5]	439242
		Inulin[5]	24763
		Sorbitol[5]	5780
		Mannitol[5]	6251
		Crocin[5]	5281233
		Aplotaxene[5]	5352710
		Clovene[5]	521210
		Dihydroaplotaxene[5]	5352709
		Docosane[5]	12405
		Eicosane[5]	8222
		1-Heptadecene[5]	23217
		Heptacosane[5]	11636
		2-Naphthalenemethanol[5]	74128
		1-Pentadecene[5]	25913
		Pentacosane[5]	12406
		Pentadecane[5]	12391

		Tetracosane[5]	12592
		Benzaldehyde[5]	240
		Butanal[5]	261
		Decanal[5]	8175
		Dodecanal[5]	8194
		Heptanal[5]	8130
		Hexanal[5]	6184
		(Z)-3-Hexenal[5]	643941
		(E)-2-Hexenal[5]	5281168
		2-Methylpropanal[5]	6561
		3-Methylbutanal[5]	11552
		Nonanal[5]	31289
		Octanal[5]	454
		Phenylacetaldehyde[5]	998
		Pentanal[5]	8063
		Propanal[5]	527
		Tridecanal[5]	25311
		4-Methoxybenzaldehyde[5]	31244
		Undecanal[5]	8186

		2-Methoxy-3-methylpyrazine[5]	17898
		2-Methoxy-3- propylpyrazine[5]	528308
		2-sec-Butyl-3-methoxypyrazine[5]	520098
		2-Isobutyl-3-methoxypyrazine[5]	32594
		2-Butyl-3- methoxypyrazine[5]	528313
		2-Isoamyl-3-methoxypyrazine[5]	528319
		Acetic acid[5]	176
		Butyric acid[5]	264
		Cinnamic acid[5]	444539
		Costic acid[5]	6451579
		Decanoic acid[5]	2969
		Dodecanoic acid[5]	3893
		Ethyl oleate[5]	5363269
		Hexanoic acid[5]	8892
		(E)-3-Hexenoic acid[5]	5282708
		Heptanoic acid[5]	8094
		(E)-3-Heptenoic acid[5]	5282710
		2-Methylpropionic acid[5]	6590
		2-Methylbutyric acid[5]	8314
		3-Methoxybenzoic acid[5]	11461
		Nonanoic acid[5]	8158
		Nonanedioic acid[5]	2266
		(E)-3-nonenoic acid[5]	5282723

		Octanoic acid[5]	379
		(E)-3-Octenoic acid[5]	5282716
		Pentanoic acid[5]	7991
		Phenylacetic acid[5]	999
		Propionic acid[5]	1032
		Pentadecanoic acid[5]	13849
		Salicylic acid[5]	338
		Tridecanoic acid[5]	12530
		Tetradecanoic acid[5]	11005
		Undecanoic acid[5]	8180
		Carvomenthone[5]	10362
		Geraniol[5]	637566
		Linalool[5]	6549
		Thymol[5]	6989
		Caryophyllene oxide[5]	1742210
		β-Costol[5]	12304104
		Aromadendrene[5]	91354
		Caryophyllene[5]	5281515
		γ-Cadinene[5]	92313
		Cyperene[5]	99856
		β-Elemene[5]	6918391
		α-Guaiene[5]	5317844
		Isoaromadendrene epoxide[5]	534398
		Limonene[5]	22311

		α-Myrcene[5]	519324
		α-Pinene[5]	6654
		Squalene[5]	638072
03	*Artemisia argyi*	γ-Terpinene[6]	7461
		O-Cymene[6]	10703
		Terpinolene[6]	11463
		α-Thujene[6]	17868
		2,5,5-Trimethyl-2,6-heptadien-4-one[6]	68346
		Yomogi alcohol[6]	5315406
		α-Thujone[6]	261491
		β-Thujone[6]	91456
		Trans-Sabinene hydrate[6]	12315151
		2,2,4-Trimethyl-3-cyclohexene-1-carbaldehyde[6]	102680
		(+)-2-Bornanone[6]	159055
		Trans-Pinocamphone[6]	11038
		Umbellulone[6]	442504
		Cis-2-Menthenol[6]	13918681
		Trans-Chrysanthenyl acetate[6]	10899521
		Bornyl acetate[6]	6448
		Dill ether[6]	126537

		1-Terpinen-4-ol[6]	11230
		Trans-Dihydrocarvone[6]	6432474
		Benihinal[6]	61130
		Trans-2,8-p-Mentha-dien-1-ol[6]	12618691
		(−)-Trans-Pinocarveol[6]	1201530
		Verbenol[6]	61126
		Borneol[6]	64685
		Cis-Sabinol[6]	12315160
		Verbenone[6]	29025
		α-Terpineol[6]	17100
		Piperitone[6]	6987
		α-Phellandren-8-ol[6]	519323
		Cis-Chrysanthenol[6]	527032
		Trans-Piperitol[6]	85568
		Myrtenol[6]	10582
		Trans-p-Mentha-1(7),8-dien-2-ol[6]	6428442
		4-Isopropyl-1,5-cyclohexadiene-1-methanol[6]	519721
		Dihydrocarveol[6]	12072

		Cis-Carveol[6]	330573
		P-Cymene-8-ol[6]	14529
		Trans-Shisool[6]	519954
		β-Ionone[6]	638014
		P-Isopropylbenzyl alcohol[6]	325
		Thymol[6]	6989
		α-Cubebene[6]	86609
		(−)-Cyperene[6]	12308843
		β-Bourbonene[6]	324224
		β-Ylangene[6]	519779
		β-Caryophyllene[6]	5281515
		α-Humulene[6]	5281520
		a-Cyperene[6]	99856
		Alloaromadendrene[6]	12305247
		Germacrene D[6]	5317570
		β-Selinene[6]	442393
		Longifolene[6]	289151
		δ-Cadinene[6]	92313
		Trans-Calamenene[6]	6429022

		Chamazulene[6]	10719
		Caryophyllene oxide[6]	1742210
		Salvial-4(14)-en-1-one[6]	42608172
		Junenol[6]	6452077
		Nerolidol[6]	5284507
		Spathulenol[6]	92231
		Neointermedeol[6]	11877394
		11,11-Dimethyl-4,8-dimethylenebicyclo[7.2.0]undecan-3-ol[6]	91715484
		Costol[6]	12304105
		3,7,11,15-Tetramethyl-2-hexadecen-1-ol[6]	5366244
		Cis-Sabinyl acetate[6]	6428460
		Bornyl isovalerate[6]	23623651
		Palmitic acid[6]	985
		Santolina triene[7]	519872
		Tricyclene[7]	79035
		α-Pinene[7]	6654
		n-Propyl-2-methyl butyrate[7]	162239
		Camphene[7]	6616
		α-Phellandrene[7]	7460

		β-Pinene[7]	14896
		2,3-Dehydro-1,8-cineole[7]	523035
		p-Cymene[7]	7463
		Eucalyptol[7]	2758
		Artemisia alcohol[7]	100197
		Isopinocarveol[7]	102667
		Trans-3-Caren-2-ol[7]	576906
		Cis-Geraniol[7]	643820
		2-Octen-4-ol[7]	5366203
		3-Carene[7]	26049
		L-Carveol[7]	11084068
		Perilla alcohol[7]	10819
		Camphor[7]	2537
		Pinocarvone[7]	121719
		Cis-3-Hexenyl isovalerate[7]	5367681
		Lavandulyl acetate[7]	30247
		Copaene[7]	19725
		Methyl eugenol[7]	7127
		β-Farnesene[7]	5281517
		β-Cubebene[7]	93081
		2-Vinylnaphthalene[7]	13230
		β-Guaiene[7]	15560252

		α-Himachalene[7]	520909
		Cembrene[7]	6430770
		Aurantiamide acetate[8]	124319
		Camelliagenin A[8]	12302281
		Apigenin[8]	5280443
		Jaceosidin[8]	5379096
		Luteolin[8]	5280445
		Eupatilin[8]	5273755
		β-sitosterol[8]	222284
		Quercetin[8]	5280343
		Umbelliferone[8]	5281426
		Daphnetin[8]	5280569
		Eriodictyol[8]	440735
		Rhamnetin[8]	5281691
		Hispidulin[8]	5281628
		Stigmasterol[8]	5280794
		Daucosterol[8]	296119
		4-hydroxy-4-methyl-2-pentanone[8]	31256
		3,3,6,8-tetramethyl-1-tetralone[8]	79420
		Selina-6-en-4-ol[8]	527220
		L-borneol[8]	10049
		Globulol[8]	101716
		Isobornyl formate[8]	62387

		Seychellene[8]	519743
		p-mentha-1,8-dien-10-ol[8]	527143
		Casticine[9]	5315263
		6-methoxytricin[9]	14034284
		Salicylic acid[9]	338
		Juniper camphor[10]	521214
		Terpinol[10]	6651
		Erucylamide[11]	5365371
		1-decene, 4-methyl-[11]	518719
		Myo-Inositol[11]	892
		α-Cadinol[11]	10398656
		2- Pyrrolidinone[11]	12025
		3-Ethylthiolane[11]	575673
		Phenylephrine[11]	6041
		Demecolcine[11]	220401
		2-Ethylacridine[11]	610161
		Artemisolide[12]	102246304
		Neochlorogenic acid[8]	5280633
		Chlorogenic acid[8]	1794427
		Cryptochlorogenic acid[8]	9798666
		Caffeic acid[8]	689043
		1,3-dicaffeoylquinic acid[8]	6474640

04	*Artemisia capillaris* Thunb.	3,4-dicaffeoylquinic acid[8]	5281780
		3,5-dicaffeoylquinic acid[8]	6474310
		4,5-dicaffeoylquinic acid[8]	10324242
		7-hydroxy-coumarin[8]	5281426
		5,7-dimethoxy-coumarin[8]	2775
		7,8-dihydroxy-coumarin[8]	5280569
		Quercetin[8]	5280343
		Kaempferol[8]	5280863
		7-methoxycoumarin[8]	10748
		4-hydroxyacetophenone[8]	7469
		Nicotinic acid[8]	938
		Thymidine[8]	5789
		Isoquercitrin[8]	5280804
		Isorhamnetin-3-O-glucoside[8]	5318645
		Ursolic acid[8]	64945
		Oleanolic acid[8]	10494
		β-sitosterol[8]	222284
		α-Pinene[8]	6654
		β-pinene[8]	14896

		Limonene[8]	22311
		1,8-cineole[8]	2758
		Piperitone[8]	6987
		β-caryophyllene[8]	5281515
		Capillin[8]	10321
		Palmitic acid[8]	985
		9,12,15-octadecatrienoic acid[8]	860
		Falcarinol[8]	5281149
		Germacrene D[8]	5317570
		(Z)-Ocimene[13]	5320250
		γ-Terpinene[13]	7461
		2-Methyl-6-methylene-1,7-octadien-3-one[13]	93231
		Cis-p-Menth-2-en-1-ol[13]	122484
		4-Terpineol[13]	11230
		p-Menth-1-en-8-ol[13]	17100
		Citronellol[13]	8842
		p-Vinylguaiacol[13]	332
		γ-Pyronene[13]	578237

		Eugenol[13]	3314
		Copaene[13]	19725
		β-Cubebene[13]	93081
		α-Cedrene[13]	6431015
		β-Farnesene[13]	5281517
		Eremophilene[13]	12309744
		Bicyclogermacrene[13]	13894537
		[illegible]-Cadinene[13]	441005
		β-Sesquiphellandrene[13]	12315492
		Trans-Nerolidol[13]	5284507
		Longicamphenylone[13]	91747202
		Spathulenol[13]	92231
		Globulol[13]	101716
		epi-α-Muurolol[13]	3084331
		α-Cadinol[13]	10398656
		Phytol[13]	5280435
		Camphor[13]	2537
		β-Elemene[13]	6918391

		α-humulene[13]	5281520
		β-myrcene[13]	31253
		Capillene[13]	3083613
		Sabinene[14]	18818
		α-Terpinene[14]	7462
		P-cymene[14]	7463
		Terpinolene[14]	11463
		Linalool[14]	6549
		Chrysanthenone[14]	442463
		Borneol[14]	64685
		Geraniol[14]	637566
		Norcapillene[14]	10983572
		α-Copaene[14]	70678558
		Geranyl acetate[14]	1549026
		Methyl eugenol[14]	7127
		β-Selinene[14]	442393
		Caryophyllene oxide[14]	1742210
		(E)-β-ocimene[15]	5281553
		α-Phellandrene[15]	7460
		Hexamethyl-benzene[15]	6908

		γ-Curcumene[15]	12304273
		Germacrene D-4-ol[15]	5352847
		epi-α-Cadinol[15]	160799
		β-Eudesmol[15]	91457
		Scoparone[16]	8417
		Cirsimaritin[16]	188323
		Arcapillin[16]	158311
		Capillone[16]	5315675
		Capillarin[16]	3083811
		Cirsilineol[16]	162464
		Capillarisin[16]	5281342
		Scopoletin[16]	5280460
		Isoscopoletin[16]	69894
		Artepillin[16]	5472440
		Isochlorogenic acid[16]	5315832
		Pumilaside A[16]	10526066
		Achillin[17]	291264
		Coumarin[17]	323
		Apigenin[17]	5280443
		Hesperidin[17]	10621
		9,12,15- Octadecatrienal[10]	5283384
		Cyclopentaneundecanoic acid[10]	534549

05	*Artemisia carvifolia* Buch.- Ham. ex Roxb.	Octane[18]	356
		Trans-2-Hexenal[18]	5281168
		Cis-3-Hexenol[18]	5281167
		2-Hexenol[18]	5318042
		1-Nonene[18]	31285
		Santolina triene[18]	519872
		Tricyclene[18]	79035
		α-Thujene[18]	17868
		α-Pinene[18]	6654
		2,7-Dimethyloxepin[18]	578868
		Camphene[18]	6616
		Ethyl tiglate[18]	5281163
		Benzaldehyde[18]	240
		Artemiseole[18]	521927
		α-Phellandrene[18]	7460
		α-Terpinene[18]	7462
		p-Cymene[18]	7463
		Limonene[18]	22311
		1,8-Cineole[18]	2758
		cis-β-Ocimene[18]	5320250

		trans-β-Ocimene[18]	5281553
		γ-Terpinene[18]	7461
		Artemisia ketone[18]	68346
		Terpinolene[18]	11463
		Methyl benzoate[18]	7150
		cis-Thujone[18]	249286
		6-Methyl-3,5-heptadien-2-one[18]	5370101
		Trans-Thujone[18]	91456
		Chrysanthenone[18]	442463
		Trans-Pinocarveol[18]	10931630
		Camphor[18]	2537
		Trans-Verbenol[18]	89664
		Borneol[18]	64685
		Terpinen-4-ol[18]	11230
		α-Terpineol[18]	17100
		Myrtenal[18]	61130
		Myrtenol[18]	10582
		Trans-Piperitol[18]	85568
		Grandisol[18]	169202
		Carvone[18]	7439
		Piperitone[18]	6987

		Bornyl acetate[18]	6448
		Eugenol[18]	3314
		α-Copaene[18]	70678558
		β-Caryophyllene[18]	5281515
		γ-Elemene[18]	6432312
		Humulene[18]	5281520
		β-Farnesene[18]	5281517
		γ-Himachalene[18]	577062
		β-Selinene[18]	442393
		Cis-Eudesma-6,11-diene[18]	639284
		Valencene[18]	9855795
		Bicyclogermacrene[18]	13894537
		α-Muurolene[18]	12306047
		γ-Cadinene[18]	92313
		d-Cadinene[18]	441005
		Eremophilene[18]	12309744
		Cis-Nerolidol[18]	5320128
		Caryophyllene oxide[18]	1742210
		Viridiflorol[18]	11996452
		Humulene epoxide II[18]	10704181

		α-Cadinol[18]	10398656
		Chamazulene[18]	10719
		Myristic acid[18]	11005
		Oleic acid[18]	445639
		Methyl palmitate[18]	8181
		Adipic acid, bis(2-ethylhexyl)ester[18]	7641
		Artemisinin[19]	68827
		Artesunate[19]	6917864
		Dihydroartemisinin[19]	456410
		Artemether[19]	68911
06	*Artemisia japonica* Thunb.	β-Amyrin[8]	73145
		Triacontanoic acid[8]	10471
		β-sitosterol[8]	222284
		Stigmasterol[8]	5280794
		7,8- dimethoxycoumarin[8]	142768
		6,7-dimethoxycoumarin[8]	8417
		Capillarisin[8]	5281342
		3,5-dihydroxy-6,7,3′,4′-tetramethoxyflavone[8]	5316832
		Cinnamic acid[8]	444539

		p-methoxybenzoic acid[8]	7478
		Ferulic acid[8]	445858
		α-Pinene[20]	6654
		Sabinene[20]	18818
		β-Pinene[20]	14896
		α-Phellandrene[20]	7460
		1,8-Cineole[20]	2758
		(Z)-β-ocimene[20]	5320250
		(E)-β-ocimene[20]	5281553
		γ-terpinene[20]	7461
		Linalool[20]	6549
		Artemisia alcohol[20]	100197
		Borneol[20]	64685
		B-Caryophyllene[20]	5281515
		Germacrene D[20]	5317570
		γ-Cadinene[20]	92313
		Trans-linalool oxide[20]	6432254
		p-Cymene[20]	7463
		Spathulenol[20]	92231

		β-elemene[20]	6918391
		α-Cubebene[21]	86609
		β-bourbonene[21]	324224
		Trans-β-farnesene[21]	5281517
		α-farnesene[21]	5281516
		δ-cadinene[21]	441005
		γ-elemene[21]	6432312
		Aromadendrene[21]	91354
		γ-Muurolene[21]	12313020
		6-isopropenyl-4,8a-dimethyl-1,2,3,5,6,7,8,8a-octahydro-naphthalen-2-ol[21]	594234
		1-pentatriacontanol[22]	558047
		Tricosanoic acid[22]	17085
		Eupatorin[22]	97214
		Berberine[23]	2353
		Palmatine[23]	19009
		Magnoflorine[23]	73337
		Jatrorrhizine[23]	72323
		Berbamine[24]	275182

07	*Berberis deinacantha* Schneid.	Isotetrandrine[24]	457825
		Columbamine[25]	72310
		Oxyacanthine[25]	442333
		Cyanidin[26]	128861
		Peonidin[26]	441773
		Petunidin[26]	441774
		Malvidin[26]	159287
		Delphinidin[26]	128853
		Pelargonidin[26]	440832
		Isoquinoline[27]	8405
		Bisbenzylisoquinoline[27]	22169421
		Phenylheptatriyne[28]	77981
		Tridec-1-ene-3,5,7,9,11-pentayne[28]	441552
		1,2-Dihydroxytrideca-5,7,9,11-tetrayne[28]	11264113
		Astragalin[28]	5282102
		Axillaroside[28]	44259807
		Apigenin 7-O-glucoside[28]	12304093
		Rutin[28]	5280805

08	*Bidens pilosa* Linn.	Querciturone[28]	5274585
		Centaurein[28]	5489090
		Jacein[28]	44259819
		Luteoside[28]	72188972
		Quercetin 3-O-glucoside[28]	5280804
		Quercetin 3-O-β-D-galactopyranoside[28]	5281643
		Sulfuretin[28]	5281295
		Okanin 3'-glucoside[28]	14213549
		Apigenin[28]	5280443
		Butein[28]	5281222
		Okanin[28]	5281294
		Centaureidin[28]	5315773
		Digitoflavone[28]	5280445
		5-O-Methylhoslundin[28]	15726099
		Benzoic acid[28]	243
		Caffeic acid[28]	689043
		Chlorogenic acid[28]	1794427
		3,4-di-O-Caffeoylquinic acid[28]	5281780
		3,5-di-O-Caffeoylquinic acid[28]	13604687
		4,5-di-O-Caffeoylquinic acid[28]	6474309

		Neochlorogenic acid[28]	5280633
		4-O-Caffeoylquinic acid[28]	9798666
		Dimethoxyphenol[28]	78828
		Eugenol[28]	3314
		Ethyl caffeate[28]	5317238
		Ferulic acid[28]	445858
		Gallic acid[28]	370
		Iso-Vanillin[28]	12127
		p-Coumaric acid[28]	637542
		Pyrocatechin[28]	289
		p-Hydroxybenzoic acid[28]	135
		Protocatechuic acid[28]	72
		p-Vinylguaiacol[28]	332
		Salicylic acid[28]	338
		Tannic acid[28]	16129778
		Vanillic acid[28]	8468
		2-Phenyl-ethanol[28]	6054
		2-Hydroxy-6-methylbenzaldehyde[28]	585174
		4-Ethyl-1,2-benzenediol[28]	70761

		Camphene[28]	6616
		(E)-β-Ocimene[28]	5281553
		m-Cymol[28]	10812
		Myrcene[28]	31253
		Limonene[28]	22311
		Perillene[28]	68316
		Sabinene[28]	18818
		Trans-Pinocarveol[28]	10931630
		Terpinolene[28]	11463
		(Z)-β-Ocimene[28]	5320250
		γ-Terpinene[28]	7461
		α-Pinene[28]	6654
		α-Phellandrene[28]	7460
		β-Pinene[28]	14896
		β-Phellandrene[28]	11142
		3-Carene[28]	26049
		(4E,6Z)-2,6-Dimethyl-2,4,6-octatriene[28]	5371125
		Borneol[28]	64685
		cis-Verbenol[28]	164888
		Linalool[28]	6549
		p-Cymen-8-ol[28]	14529

		Terpinen-4-ol[28]	11230
		Trans-Verbenol[28]	89664
		α-Terpineol[28]	17100
		1,8-Cineole[28]	2758
		Acorenone B[28]	21674978
		Allo-Aromadendrene[28]	42608158
		Bicyclogermacrene[28]	13894537
		(+)-Epi-bicyclosesquiphellandrene[28]	521496
		Cis-Calamenen-10-ol[28]	91749818
		Cyclosativene[28]	519960
		Daucene[28]	177773
		Epi-Longipinanol[28]	91746617
		Elixene[28]	94254
		(E)-β-Farnesene[28]	10407
		Germacrene A[28]	5835162
		Germacrene-D[28]	6436582
		Humulene oxide II[28]	10704181
		Isoledene[28]	530426
		Selina-3,7(11)-diene[28]	522296
		Trans-Calamenen-10-ol[28]	10798883
		Valencene[28]	9855795

		β-Cedrene[28]	11106485
		β-Selinene[28]	442393
		α-Cadinol[28]	10398656
		α-Calacorene[28]	12302243
		α-Bergamotene[28]	86608
		α-Copaene[28]	70678558
		α-Cubebene[28]	86609
		α-Gurjunene[28]	15560276
		α-Humulene[28]	5281520
		α-Muurolene[28]	12306047
		α-Ylangene[28]	442409
		β-Bourbonene[28]	324224
		β-Bisabolene[28]	10104370
		β-Caryophyllene[28]	5281515
		β-Cubebene[28]	93081
		β-Elemene[28]	6918391
		β-Gurjunene[28]	6432176
		γ-Cadinene[28]	92313
		γ-Muurolene[28]	12313020
		[illegible]-Cadinol[28]	12302222
		[illegible]-Elemene[28]	12309449
		[illegible]-Cadinene[28]	441005
		1-epi-Cubenol[28]	519857

		Caryophyllene oxide[28]	1742210
		Epi-cedrol[28]	6713078
		(E)-nerolidol[28]	5284507
		Precocene 1[28]	28619
		Spathulenol[28]	92231
		T-Muurolol[28]	3084331
		Pimaradiene[28]	440909
		Phytol[28]	5280435
		Phytenic acid[28]	5282676
		1-Eicosene[28]	18936
		Friedelin[28]	91472
		Lupeol[28]	259846
		Lupeol acetate[28]	92157
		Squalene[28]	638072
		β-Amyrin[28]	73145
		B-Carotene[28]	5280489
		Bornyl acetate[28]	6448
		Caryophylla-4(14),8(15)-dien-5-ol[28]	6428430
		Cis-3-Hexen-1-ol[28]	5281167
		Cis-3-Hexenyl acetate[28]	5363388
		Cis-Chrysanthenyl acetate[28]	6431301
		Diphenylenemethane[28]	6853
		(E)-Geranyl acetone[28]	1713001

		Hexadecanol[28]	2682
		Hexahydrofarnesylacetone[28]	10408
		Hexadecyl acetate[28]	12393
		Isophorone[28]	6544
		Mesitylene[28]	7947
		Methyl hexadecanoate[28]	8181
		Methyl linoleate[28]	5284421
		n-Tricosane[28]	12534
		n-Decane[28]	15600
		n-Dodecane[28]	8182
		n-Docosane[28]	12405
		n-Tetradecane[28]	12389
		n-Hexadecane[28]	11006
		n-Heptadecane[28]	12398
		n-Heneicosane[28]	12403
		n-Octadecane[28]	11635
		n-Pentadecane[28]	12391
		Pentadecanal[28]	17697
		Octadecadienol[28]	71317672
		Nonanal[28]	31289
		Pseudocumene[28]	7247
		1-Heptadecene[28]	23217
		1-Octadecene[28]	8217

		2,5,9-Trimethylcycloundeca-4,8 dienone[28]	5369030
		6-Methyl-5-hepten-2-one[28]	9862
		Decanal[28]	8175
		Tridecane[28]	12388
		Pheophytin a[28]	135398712
		Behenic acid[28]	8215
		2-Butoxyethyl linoleate[28]	87553426
		Methyl linolenate[28]	5319706
		Linolenic acid[28]	5280934
		Capric acid[28]	2969
		Elaidic acid[28]	637517
		Myristic acid[28]	11005
		Lauric acid[28]	3893
		Linoleic acid[28]	5280450
		Palmitic acid[28]	985
		Palmitoleic acid[28]	445638
		Campestrol[28]	134688997
		Daucosterol[28]	296119
		Stigmasterol[28]	5280794
		β-Sitosterol[28]	222284
		Aesculetin[28]	5281416
		Caffeine[28]	2519
		(E)-Butenedioic acid[28]	444972

		Butanedioic acid[28]	1110
		2-Butoxy ethanol[28]	8133
		α-Tocopheryl quinone[28]	24205
		Tetracosane[29]	12592
		Pentacosane[29]	12406
		Hexacosane[29]	12407
		Heptacosane[29]	11636
		Nonacosane[29]	12409
		Triacontane[29]	12535
		Hentriacontane[29]	12410
		Dotriacontane[29]	11008
		Tritriacontane[29]	12411
		Tetracosan-1-ol[29]	10472
		Hexacosan-1-ol[29]	68171
		1-Octacosanol[29]	68406
		1-Hentriacontanol[29]	68345
		Stearic acid[29]	5281
		Arachidic acid[29]	10467
		Oleic acid[29]	445639
		Ethyl linoleate[29]	5282184
		Ethyl linolenate[29]	5367460
		2-Butoxyethyl oleate[29]	6436064
		1,11-Tridecadiene3,5,7,9-tetrayne[29]	5322026
		(2R,3E,11E)-3,11-Tridecadiene-5,7,9-triyne-1,2-diol[29]	6442707

		Pilosol A[29]	21593834
		7-Phenyl-2,4,6- heptatriyn-1-ol[29]	3085176
		Luteolin 7-O- [illegible]-Dglucopyranoside[29]	13093777
		5-Hydroxy-2- (3-hydroxy-4-methoxyphenyl)- 6,7-dimethoxy-4H-chromen-4-one[29]	97214
		Quercetin[29]	5280343
		E-Caryophyllene[29]	5281522
		Germacrene D[29]	5317570
		Phytanic acid[29]	26840
		[illegible]-Sitosterol glucoside[29]	5742590
		Vanillin[29]	1183
		Bidenphytin A[29]	101838305
		Bidenphytin B[29]	101838306
		Thymidine[29]	5789
		2-Acetyl-thiophene[29]	6920
09	*Bletilla formosana* (Hayata) Schltr.	Nudol[30]	158975
		2,7-dihydroxy-3,4,6-trimethoxyphenanthrene[30]	356766
		3,7-dihydroxy-2,4-dimethoxyphenanthrene[30]	10445823
		Erianthridin[30]	10401022
		Ephemeranthoquinone[30]	10038025
		Blestrianol A[30]	14863073
		Phochinenin K[30]	102477417
		Cirrhopetalanthrin[30]	442695

		Agrostonin[30]	44600287
		3-O-methyldihydropinosylvin[30]	636980
		Batatasin III[30]	10466989
		3′-O-methylbatatasin III[30]	442711
		Gigantol[30]	3085362
		3,3′-dihydroxy-4-(4-hydroxybenzyl)-5-methoxybibenzyl[30]	102316541
		Bulbocodin D[30]	102316584
		3,3′-dihydroxy-2-(4-hydroxybenzyl)-5-methoxybibenzyl[30]	91542987
		4-hydroxybenzyl ethyl ether[30]	93781
		4-hydroxybenzaldehyde[30]	126
		Militarin[30]	171638
		Shancigusin I[30]	102582111
		Gastrodin[30]	115067
		Dactylorhin A[30]	10819499
		5- hydroxymethylfuraldehyde[30]	237332
		Densiflorol B[30]	637413
		3,5-dimethoxy-3′-hydroxybibenzyl[30]	132427497
		β-sitosterol[30]	222284
		Stigmasterol[30]	5280794
		Ethyl (E)-4-hydroxycinnamate[30]	676946
		Excelsioside[31]	101637168

		Gymnoside IX[31]	11651021
		Benzyl alcohol[31]	244
		Coelonin[31]	11390848
		Cinnamic acid[31]	444539
10	*Blumea balsamifera* (L.) DC.	L-borneol[32]	10049
		Isoborneol[32]	6321405
		(+)-Limonene[32]	440917
		(−)-Limonene[32]	439250
		(Z)-β-Ocimene[32]	5320250
		β-Myrcene[32]	31253
		Camphene[32]	6616
		α-Pinene[32]	6654
		β-Pinene[32]	14896
		Terpinen-4-ol[32]	11230
		Perillyl alcohol[32]	10819
		Chrysanthenone[32]	442463
		Bornyl acetate[32]	6448
		Sabinene[32]	18818
		α-Thujene[32]	17868
		Trans-linalool oxide[32]	6432254

		Linalooloxide[32]	22310
		Camphor[32]	2537
		1,8-Cineole[32]	2758
		Perilla aldehyde[32]	16441
		Cuminaldehyde[32]	326
		Myrtenal[32]	61130
		Thymohydroquinone dimethyl ether[32]	6427071
		α-Gurjunene[32]	15560276
		Alloaromadendrene[32]	12305247
		(+)-Aromadendrene[32]	11095734
		Aromadendrene[32]	91354
		Aromadendrene oxide[32]	91753455
		Aromadendrene, dehydro[32]	589433
		Longifolene[32]	289151
		β-Caryophyllene[32]	5281515
		Caryophyllene oxide[32]	1742210
		Guaia-3,9-diene[32]	585005
		γ-Cadinene[32]	92313
		δ-Cadinene[32]	441005

		β-Selinene[32]	442393
		β-Gurjunene[32]	6432176
		(+)-γ-Gurjunene[32]	15560285
		β-Elemene[32]	6918391
		Globulol[32]	101716
		(−)-Guaiol[32]	227829
		Ledol[32]	92812
		γ-Muurolene[32]	12313020
		Elemol[32]	92138
		β-Eudesmol[32]	91457
		γ-Eudesmol[32]	6432005
		Carotol[32]	442347
		Cubenol[32]	519857
		16-Kaurene[32]	520687
		Phytol[32]	5280435
		(11Z)-11-hexadecenoic acid[32]	5312414
		Trans-2-undecenoic acid[32]	5282728
		9-Hexadecenoic acid[32]	5282745
		Capric acid[32]	2969

		Palmitic acid[32]	985
		Xanthoxylin[32]	66654
		Eugenol[32]	3314
		Dimethoxydurene[32]	601765
		1-Octen-3-ol[32]	18827
		3-Octanol[32]	11527
		3-Octanone[32]	246728
		Paracymene[32]	7463
		3-Nitrophthalic acid[32]	69043
		Luteolin[32]	5280445
		Luteolin-7-methyl-ether[32]	5318214
		Diosmetin[32]	5281612
		Chrysoeriol[32]	5280666
		Quercetin[32]	5280343
		3,5,3',4'-Tetrahydroxy-7-methoxyflavone[32]	5281691
		3,5,3'-Trihydroxy-7,4-dimethoxyflavone[32]	5320287
		Tamarixetin[32]	5281699
		3,5-Dihydroxy-3',4',7-trimethoxyflavone[32]	5280682
		5,7-Dihydroxy-3,3',4',-trimethoxyflavone[32]	5383438
		Chrysosplenol C[32]	189065

		4',5,7-Trihydroxy-3,3'-dimethoxyflavone[32]	5316900
		Hyperoside[32]	5281643
		Isoquercitrin[32]	5280804
		Blumeatin[32]	70696494
		Eriodictyol[32]	440735
		5,7,3',5'-Tetrahydroxyflavanone[32]	11483087
		3,3',4',5-Tetrahydroxy-7-methoxyflavanone[32]	12313900
		3,3',5-Trihydroxy-4',7-dimethoxyflavanone[32]	11256019
		Catechin[32]	9064
		Davidioside[32]	42607667
		Davidigenin[32]	442342
		Blumealactone A[32]	14021255
		Blumealactone B[32]	14021258
		Blumealactone C[32]	14021261
		β-Sitosterol[32]	222284
		Daucosterol[32]	296119
		Cryptomeridiol[32]	4655876
		Austroinulin[32]	70698052
		Syringaresinol[32]	100067
		7-hydroxycoumarin[32]	5281426
		5,7-Dihydroxychromone[32]	5281343

		6-undecanol[33]	32045
		Limonene[33]	22311
		Linalool[33]	6549
		Borneol[33]	64685
		Neryl acetate[33]	1549025
		Acetic acid[33]	176
		Patchoulene[33]	91746471
		Epicedrol[33]	522667
		Geranyl iso-valerate[33]	5362830
		Germacrene -D-4-ol[33]	5352847
		Tetracyclo [6,3,2,0,(2.5).0(1,8) tridecan-9-ol, 4,4-dimethyl][33]	585744
		Cycloisolongifolene, 8,9-dehydro[33]	594593
		Hotrienol[34]	5366264
		α-Terpineol[34]	17100
		(Z)-Carveol[34]	330573
		Geraniol[34]	637566
		Thymol[34]	6989
		δ-Elemene[34]	12309449
		α-Cubenene[34]	86609
		Isocaryophyllene[34]	5281522
		Geranyl acetone[34]	1549778
		α-Selinene[34]	10856614
		α-Muurolene[34]	12306047
		α-Calacorene[34]	12302243

		Nerolidol[34]	5284507
		Caryolan-8-ol[34]	91746499
		Caryophyllene epoxide[34]	14350
		Viridiflorol[34]	11996452
		Rosifoliol[34]	527256
		Juniper camphor[34]	521214
		Pentadecanal[34]	17697
		(2Z,6E)- Farnesol[34]	1549108
		Zierone[34]	91752839
		Myristic acid[34]	11005
		1-Nonadecene[34]	29075
		Pentadecanoic acid[34]	13849
		Isophytol[34]	10453
		Ethanol[35]	702
		3-hydroxy-2-butanone[35]	179
		Thujopsene[35]	442402
		4,4-dimethyl-3-(3-methylbut-3-enylidene)-2-methylenebicyclo[4.1.0]heptane[35]	5371839
		7-methyl-3-methylene-6-octen-1-ol[35]	518689
		3-t-butyl-4-methoxyphenol methyl derivative[35]	88792
		Diepicedrene-1-oxide[35]	534683
		1,2-dimethoxy-4-(2-propenyl)-benzene[35]	7127
		Epiglobulol[35]	11858788

		2-methoxy-3-(2-propenyl)-phenol[35]	596373
		Samboginone[36]	52936973
		Velutin[36]	5464381
		Pachypodol[36]	5281677
		4'-O-methyldihydroquercetin[36]	482576
		2,4-Dicumylphenol[37]	76013
		p-Hydroxybenzoic acid[37]	135
		Gentisic acid[37]	3469
		β-Daucosterol[37]	5742590
		3,4',5-Trihydroxy-3',7-dimethoxyflavanone[38]	14353345
		3',4',5-trihydroxy-7-methoxyflavanone[38]	181132

References

1. Ma, J. P., Guo, Z. B., Jin, L. & Li, Y. D. Phytochemical progress made in investigations of Angelica sinensis (Oliv.) Diels. *Chin. J. Nat. Med.* **13**, 241–249 (2015).

2. Tabanca, N. *et al.* Chemical composition and antifungal activity of Angelica sinensis essential oil against three Colletotrichum species. *Nat. Prod. Commun.* **3**, 1073–1078 (2008).

3. Champakaew, D. *et al.* Assessment of Angelica sinensis (Oliv.) Diels as a repellent for personal protection against mosquitoes under laboratory and field conditions in northern Thailand. *Parasites and Vectors* **9**, 1–14 (2016).

4. Wei, W. L., Zeng, R., Gu, C. M., Qu, Y. & Huang, L. F. Angelica sinensis in China-A review of botanical profile, ethnopharmacology, phytochemistry and chemical analysis. *J. Ethnopharmacol.* **190**, 116–141 (2016).

5. Wang, D. *et al.* Arctium species secondary metabolites chemodiversity and bioactivities. *Front. Plant Sci.* **10**, (2019).

6. Guan, X. *et al.* Chemical Composition and Antimicrobial Activities of Artemisia argyi Lévl. Et vant essential oils extracted by simultaneous distillation-extraction, subcritical extraction and hydrodistillation. *Molecules* **24**, (2019).

7. Jiang, Z. T., Tan, J., Tan, J. & Li, R. Chemical Components and Molecular Microcapsules of Folium Artemisia argyi Essential Oil with β-Cyclodextrin Derivatives. *J. Essent. Oil-Bearing Plants* **19**, 1155–1169 (2016).

8. Nigam, M. *et al.* Bioactive compounds and health benefits of Artemisia species. *Nat. Prod. Commun.* **14**, (2019).

9. Zhang, L. Bin, Lv, J. L., Chen, H. L., Yan, X. Q. & Duan, J. A. Chemical constituents from Artemisia argyi and their chemotaxonomic significance. *Biochem. Syst. Ecol.* **50**, 455–458 (2013).

10. Abad, M. J., Bedoya, L. M., Apaza, L. & Bermejo, P. The Artemisia L. genus: A review of bioactive essential oils. *Molecules* **17**, 2542–2566 (2012).

11. Ahmed, M. *et al.* Determination of phytochemicals, antioxidant activity and biochemical composition of Chinese Mugwort (Artemisia argyi L.) leaf extract from Northeast China. *Appl. Ecol. Environ. Res.* **17**, 15349–15362 (2019).

12. Kim, J. H. *et al.* New sesquiterpene-monoterpene lactone, artemisolide, isolated from Artemisia argyi. *Tetrahedron Lett.* **43**, 6205–6208 (2002).

13. Liu, Z. L., Chu, S. S. & Liu, Q. R. Chemical composition and insecticidal activity against sitophilus zeamais of the essential oils of artemisia capillaries and artemisia mongolica. *Molecules* **15**, 2600–2608 (2010).

14. Verma, R. S. *et al.* Chemical composition of volatile fraction of fresh and dry artemisia capillaris thunb. from kumaon Himalaya. *J. Essent. Oil-Bearing Plants* **13**, 118–122 (2010).

15. Joshi, R. K., Padalia, R. C. & Mathela, C. S. Phenyl alkynes rich essential oil of Artemisia capillaris. *Nat. Prod. Commun.* **5**, 815–816 (2010).

16. Jang, E., Kim, B. J., Lee, K. T., Inn, K. S. & Lee, J. H. A survey of therapeutic effects of artemisia capillaris in liver diseases. *Evidence-based Complement. Altern. Med.* **2015**, (2015).

17. Koul, B. & Taak, P. The Artemisia Genus: A Review on Traditional Uses, Phytochemical Constituents, Pharmacological Properties and Germplasm Conservation. *J. Glycomics Lipidomics* **07**, 1–7 (2018).

18. Yang, Y. *et al.* CHEMICAL COMPOSITION AND ANTIMICROBIAL ACTIVITY OF THE ESSENTIAL OIL FROM Artemisia carvifolia LEAVES. **51**, 140–141 (2015).

19. Pala, Z., Shukla, V., Alok, A. & Kudale, S. Enhanced production of an anti-malarial compound artesunate by hairy root cultures and phytochemical analysis of Artemisia pallens Wall . *3 Biotech* **6**, 1–8 (2016).

20. Joshi, R. K. Volatile oil composition of Artemisia japonica Thunb . from Western Himalaya of Uttarakhand. **3**, 96–97 (2015).

21. Kerala, R. T. R., Francis, M. S. & Soumya, M. Essential oil composition of Artemisia japonica Thunb. *J. Pharmacogn. Phytochem.* **3**, 160–163 (2014).

22. Giang, P. M., Binh, N. T., Matsunami, K. & Son, P. T. Three new eudesmanes from Artemisia japonica. *Nat. Prod. Res.* **28**, 631–635 (2014).

23. Belwal, T. *et al.* Phytopharmacology and Clinical Updates of Berberis Species Against Diabetes and Other Metabolic Diseases. *Front. Pharmacol.* **11**, (2020).

24. Berberidaceae, I. N. B. Review article : A REVIEW ON BIOLOGICAL AND CHEMICAL DIVERSITY. *EXCLI J.* 247–267 (2015).

25. Bhardwaj, D. & Kaushik, N. Phytochemical and pharmacological studies in genus Berberis. *Phytochem. Rev.* **11**, 523–542 (2012).

26. Gholizadeh-Moghadam, N., Hosseini, B. & Alirezalu, A. Classification of barberry genotypes by multivariate analysis of biochemical constituents and HPLC profiles. *Phytochem. Anal.* **30**, 385–394 (2019).

27. Sequeda-Castañeda, L. G. *et al.* Preliminary phytochemical analysis of Berberis goudotii Triana & Planch. ex wedd. (berberidaceae) with anticariogenic and antiperiodontal activities. *Sci. Pharm.* **87**, (2019).

28. Xuan, T. D. & Khanh, T. D. Chemistry and pharmacology of Bidens pilosa: an overview. *J. Pharm. Investig.* **46**, 91–132 (2016).

29. Bartolome, A. P., Villaseñor, I. M. & Yang, W. C. Bidens pilosa L. (Asteraceae): Botanical properties, traditional uses, phytochemistry, and pharmacology. *Evidence-based Complement. Altern. Med.* **2013**, (2013).

30. Lin, C. W. *et al.* Chemical Constituents of the Rhizomes of Bletilla formosana and Their Potential Anti-inflammatory Activity. *J. Nat. Prod.* **79**, 1911–1921 (2016).

31. Wu, T. Y. & Lay, H. L. Effect of growth stages, culture media, and processing methods on the component variations of Bletilla formosana and comparison of its component contents to commercial Rhizoma Bletillae crude drugs. *J. Food Drug Anal.* **21**, 404–413 (2013).

32. Pang, Y. *et al.* Blumea balsamifera- A phytochemical and pharmacological review. *Molecules* **19**, 9453–9477 (2014).

33. Laboratories, B. & Cantonment, P. O. C. - Short communication CHEMICAL COMPONENTS IN VOLATILE OIL FROM. **38**, 107–109 (2009).

34. Wang, Y. H. & Zhang, Y. R. Variations in compositions and antioxidant activities of essential oils from leaves of Luodian Blumea balsamifera from different harvest times in China. *PLoS One* **15**, 1–15 (2020).

35. Jiang, Z. L., Zhou, Y., Ge, W. C. & Yuan, K. Phytochemical compositions of volatile oil from Blumea balsamifera and their biological activities. *Pharmacogn. Mag.* **10**, 346–352 (2014).

36. Saifudin, A., Tanaka, K., Kadota, S. & Tezuka, Y. Chemical constituents of Blumea balsamifera of Indonesia and their protein tyrosine phosphatase 1B inhibitory activity. *Nat. Prod. Commun.* **7**, 815–818 (2012).

37. Tan, D., Yan, Q. & Kang, H. Chemical constituents from Blumea balsamifera. *Chem. Nat. Compd.* **48**, 1072–1073 (2013).

38. Ali, D. M. H., Wong, K. C. & Lim, P. K. Flavonoids from Blumea balsamifera. *Fitoterapia* **76**, 128–130 (2005).

Serial No.	Plant name	Chemical name	Pubchem CID
01	*Bletilla striata* (Thunb. ex A. Murray) Rchb. f.	Dactylorhin A[1]	10819499
		Gymnoside II[1]	11619501
		Gymnoside V[1]	11693717
		Gymnoside IX[1]	11651021
		Gymnoside X[1]	11586164
		Militarine[1]	171638
		Gastrodin[1]	115067
		Blestritin A[1]	101845671
		Blestritin B[1]	101845672
		Blestritin C[1]	101845673
		Bulbocodin C[1]	102316583
		Bulbocodin D[1]	102316584
		Bulbocol[1]	102316540
		Shancigusin B[1]	42642927
		Shanciguol[1]	101995286
		Batatasin III[1]	10466989
		Gigantol[1]	3085362
		3’-O-methylbatatasin III[1]	442711
		3,3’-dihydroxy-5-methoxy-2,5’,6-tris(p -hydroxybenzyl) bibenzyl[1]	87579595
		3,3’,5-trimethoxybibenzyl[1]	15693460

		3,5-dimethoxybibenzyl[1]	10538117
		3,5-dimethoxyphenanthrene-2,7-diol[1]	44572330
		1,5-dimethoxyphenanthrene-2,7-diol[1]	158976
		2,4,7-trimethoxyphenanthrene[1]	15693458
		2,3,4,7-tetramethoxyphenanthrene[1]	11437978
		2,7-dihydroxy-3,4-dimethoxyphenanthrene[1]	158975
		Blespirol[1]	102440970
		1,8-dihydroxy-3-methoxy-6-methylanthracene-9,10-dione[1]	10639
		2-methylanthraquinone[1]	6773
		7-hydroxy-2-methoxyphenanthrene-3,4-dione[1]	127031491
		Blestrin A[1]	14583570
		Blestrin B[1]	14583572
		Blestrin C[1]	101636138
		Blestrin D[1]	101634591
		Blestriarene B[1]	442695
		Blestriarene C[1]	9982511
		Blestrianol A[1]	14863073
		Blestrianol B[1]	14863075
		Blestrianol C[1]	14863077
		4,7-dihydroxy-2-methoxy-9,10-dihydrophenanthrene[1]	442702

		2,4,7-trimethoxy-9,10-dihydrophenanthrene[1]	15693459
		Pleionesin C[1]	102097659
		Shanciol[1]	44257051
		β-sitosterol[1]	222284
		β-sitosterol palmitate[1]	13747834
		Stigmasterol[1]	5280794
		3-epiruscogenin[1]	12315110
		3-epineoruscogenin[1]	21626040
		Cyclomargenol[1]	101658822
		Cyclomargenone[1]	101658823
		Cycloneolitsol[1]	101306728
		Cyclobalanone[1]	101306798
		24-methylenecycloartanol palmitate[1]	101933290
		Cyclolaudenol[1]	101729
		Cyclolaudenone[1]	21592246
		p-hydroxybenzoic acid[1]	135
		Protocatechuic acid[1]	72
		Cinnamic acid[1]	444539
		Caffeic acid[1]	689043
		2-hydroxysuccinic acid[1]	525

		Palmitic acid[1]	985
		Syringaresinol[1]	100067
		Pinoresinol[1]	73399
		p-hydroxybenzaldehyde[1]	126
		Ferulic acid[1]	445858
		3-hydroxycinnamic acid[1]	637541
		4-hydroxybenzylamine[1]	97472
		4,4'-dihydroxydiphenylmethane[1]	12111
		5-(hydroxymethyl)-2-furaldehyde[1]	237332
		Schizandrin[1]	3001664
		Tupichinol A[1]	637885
		4-hydroxybenzyl alcohol[2]	125
		4-hydroxybenzyl β-D-glucopyranoside[2]	49871127
		Shancigusin I[3]	102582111
		3,7-dihydroxy-2,4-dimethoxyphenanthrene[4]	10445823
		Bibenzyl[5]	7647
		Biphenanthrene[5]	21709721
		Benzylphenanthrene[5]	21569412
		Dihydrophenanthropyran[5]	87098366
		Coelonin[6]	11390848

		Gymnoside III[6]	134715148
		Flavanthrinin[6]	14777892
		3,3′-Dihydroxy-2,6-bis(4-hydroxybenzyl)-5-methoxybibenzyl[6]	11282492
02	*Boenninghausenia albiflora* (Hook.) Rchb. ex Meisn.	α-Pinene[7]	6654
		Sabinene[7]	18818
		β-Pinene[7]	14896
		β-Myrcene[7]	31253
		p-Cymene[7]	7463
		Limonene[7]	22311
		(E)-β-Ocimene[7]	5281553
		γ-Terpinene[7]	7461
		Terpinolene[7]	11463
		1,8-Cineole[7]	2758
		Cis-Sabinene hydrate[7]	101629835
		Linalool[7]	6549
		Perillene[7]	68316
		Cis-p-Menth-2-en-1-ol[7]	122484
		Borneol[7]	64685
		Terpinen-4-ol[7]	11230
		p-Cymen-8-ol[7]	14529

		Verbenone[7]	29025
		Cumin aldehyde[7]	326
		Bornyl acetate[7]	6448
		α-Terpinyl acetate[7]	111037
		p-Cymen-7-ol acetate[7]	100990
		α-Cubebene[7]	86609
		β-Cubebene[7]	93081
		β-Bourbonene[7]	324224
		β-Elemene[7]	6918391
		β-Caryophyllene[7]	5281515
		Seychellene[7]	519743
		α-Humulene[7]	5281520
		Germacrene D[7]	5317570
		Bicyclogermacrene[7]	13894537
		Germacrene A[7]	9548705
		δ-Cadinene[7]	441005
		Selina-3,7(11)-diene[7]	522296
		Germacrene B[7]	5281519
		Epi-Cubebol[7]	91753433
		(E)-Nerolidol[7]	5284507
		Germacren D-4-ol[7]	5352847
		Spathulenol[7]	92231

		Caryophyllene oxide[7]	1742210
		Globulol[7]	101716
		Fokienol[7]	5352449
		Guaiol[7]	227829
		β-Oplopenone[7]	14038847
		Humulene epoxide II[7]	10704181
		Epi-α-Cadinol[7]	160799
		α-Muurolol[7]	100949538
		α-Eudesmol[7]	92762
		α-Cadinol[7]	10398656
		Bulnesol[7]	90785
		Khusinol[7]	91746535
		Epi-α-Bisabolol[7]	1201551
		Xanthorrhizol[7]	93135
		n-Decanal[7]	8175
		Decyl acetate[7]	8167
		n-dodecanol[7]	8193
		Geijerene[7]	12310053
		Isogeijerene C[7]	85863764
		Pregeijerene[7]	21160126
		Dictamnol[7]	91746503

		α-Thujene[8]	17868
		Camphene[8]	6616
		(Z)-β-Ocimene[8]	5320250
		(Z)-Sabinene hydrate[8]	62367
		(Z)-p-Menth-2-en-1-ol[8]	13918681
		(E)-Pinocamphone[8]	11038
		Myrtenol[8]	10582
		2-Undecanone[8]	8163
		2-Undecanol[8]	15448
		δ-Elemene[8]	12309449
		Piperitenone[8]	381152
		Methyl perillate[8]	14159029
		(E)-β-Farnesene[8]	10407
		γ-Gurjunene[8]	90805
		(Z)-Nerolidol[8]	5356544
		4-(4-tert-butyl phenoxy)-Benzaldehyde[8]	10824840
		Benzaldehyde[9]	240
		1-Propylcyclopentanol[9]	98267
		δ-3-Carene[9]	26049
		Salicylaldehyde[9]	6998

		4-Methyl-4-vinylbutyrolactone[9]	10975491
		Acetophenone[9]	7410
		Cis-Linalool oxide[9]	6428573
		Trans-Linalool Oxide[9]	6432254
		α-Campholenal[9]	1252759
		Nopinone[9]	32735
		Linderol[9]	1201518
		Hydrocinnamaldehyde[9]	7707
		3,7-Dimethyloct-1,5-dien-3,7-diol[9]	5352451
		α-Terpineol[9]	17100
		(Z)-Cinnamaldehyde[9]	6428995
		Bornyl formate[9]	518472
		(E)-cinnamaldehyde[9]	637511
		(E)-Cinnamyl alcohol[9]	5315892
		Ethyl-Hydrocinnamate[9]	16237
		Cinnamyl formate[9]	5354883
		Cis-Cinnamic acid[9]	5372954
		α-Copaene[9]	70678558
		Hydrocinnamyl acetate[9]	31226
		Cinnamyl propyl ether[9]	5369728
		(Z)-ethyl-Cinnamate[9]	5284656
		β-selinene[9]	442393
		Guaia-1(10),11-diene[9]	520826

		α-Muurolene[9]	12306047
		γ-Cadinene[9]	92313
		Trans-Calamenene[9]	6429022
		Caryophylla-4(12),8(13)-dien-5-alpha-ol[9]	527418
		Cadin-4-en-10-ol[9]	519662
		α- Costol[9]	13006421
		Kaur-16-ene[9]	5318786
		Linalyl cinnamate[9]	5355858
		Coumarin[10]	323
		Murraxocin[10]	188750
		Murralongin[10]	179620
03	*Bombax ceiba* L.	Quercetin[11]	5280343
		Quercetin-3-O-β-D-glucopyranoside[11]	5280804
		Quercetin-3-O-β-D-glucuronopyranoside[11]	5274585
		Rutin[11]	5280805
		Vitexin[11]	5280441
		Isovitexin[11]	162350
		Vicenin[11]	3084407
		Kaempferol-3-O-rutinoside[11]	5318767
		Isomangiferin[11]	5318597
		Mangiferin[11]	5281647
		Esculetin[11]	5281416

		Scopoletin[11]	5280460
		Fraxetin[11]	5273569
		Scopoline[11]	261184
		Blumenol C glucoside[11]	14135395
		Benzyl-β-Dglucopyranoside[11]	13254166
		Phenylethyl rutinoside[11]	11166301
		Chlorogenic acid[11]	1794427
		Methyl chlorogenate[11]	6476139
		Vanillic acid[11]	8468
		Lupeol[12]	259846
		β- sitosterol[12]	222284
		Potassium nitrate[12]	24434
		7- hydroxycadalene[12]	608115
		Kaempferol[12]	5280863
		Ethyl palmitate[12]	12366
		Bombasin[12]	24787302
		Bombalin[12]	101839172
		Neochlorogenic acid[12]	5280633
		Quercetagetin[12]	5281680
		Cholesterol[12]	5997
		Stigmasterol[12]	5280794
		Campesterol[12]	173183
		α-amyrin[12]	73170

		Vicenin 2[12]	442664
		Linarin[12]	5317025
		Saponarin[12]	441381
		Cosmetin[12]	5280704
		Xanthomicrol[12]	73207
		Apigenin[12]	5280443
		Fructose[12]	2723872
		Glucose[12]	5793
		Galactose[12]	6036
		Arabinose[12]	439195
		Sucrose[12]	5988
		Lactose[12]	6134
		N-hexacosanol[12]	68171
		Myristic acid[12]	11005
		Arachidic acid[12]	10467
		Behenic acid[12]	8215
		Galacturonic acid[12]	439215
		Rhamnose[12]	25310
		Palmitic acid[12]	985
		Stearic acid[12]	5281
		Oleic acid[12]	445639
		Linoleic acid[12]	5280450
		Bombaxquinone B[13]	328066

		Hemigossypolone[13]	182249
		Bombamalone A[13]	23642715
		Bombamalone B[13]	23642716
		Bombamalone C[13]	23642717
		Bombamalone D[13]	44445623
		Gossypol[13]	3503
		Hemigossypol[13]	115300
		Isohemigossypol-1-methyl ether[13]	157642
		Isohemigossypol-2-methyl ether[13]	186727
		Lacinilene C[13]	170551
		Gallic acid[13]	370
		Ethyl gallate[13]	13250
		1-Galloyl-beta-d-glucose[13]	124021
		Tannic acid[13]	16129778
		Alpha-Cedrol[13]	65575
		Triacontanol[13]	68972
		3-Methyl-2(3H)-benzofuranone[13]	577536
		Vernolic acid[13]	6449780
		Octyl palmitate[13]	85651
		Octadecyl palmitate[13]	75778
		Cellulose[13]	16211032
		Pentosan[13]	125409

04	*Bryophyllum pinnatum* (Lam.) Oken	Hexadecanoic acid, ethyl ester[14]	12366
		9-Octadecenoic acid, methyl ester[14]	5280590
		Linoleic acid ethyl ester[14]	5282184
		9,12-Octadecadienoic acid, ethyl ester[14]	5365672
		Syringic acid[15]	10742
		Caffeic acid[15]	689043
		4-hydroxy-3-methoxy-cinnamic acid[15]	709
		4-hydroxybenzoic acid[15]	135
		p-hydroxycinnamic acid[15]	637542
		Ferulic acid[15]	445858
		Protocatechuic acid[15]	72
		Phosphoenolpyruvate[15]	1005
		Astragalin[15]	5282102
		3,8-dimethoxy-4,5,7-trihydroxyflavone[15]	13983738
		Friedelin[15]	91472
		Luteolin[15]	5280445
		Rutin[15]	5280805
		Kaempferol[15]	5280863
		Quercetin[15]	5280343
		α-amyrin[15]	73170
		β-amyrin[15]	73145
		B-amyrinacetate[15]	92156

		Taraxerol[15]	92097
		Ψ-taraxasterol[15]	5270605
		Glutinol[15]	9932254
		β-sitosterol[15]	222284
		Bryophyllin B[15]	101424435
		Bryophyllin A[15]	5488801
		Bersaldegenin-3-acetate[15]	21768173
		Bryotoxin A[15]	441848
		Bryotoxin B[15]	5489391
		Campesterol[15]	173183
		Isofucosterol[15]	5281326
		Clionasterol[15]	457801
		Codisterol[15]	13833114
		Peposterol[15]	5321504
		22- dihydrobrassicasterol[15]	312822
		Clerosterol[15]	5283638
		24-epiclerosterol[15]	185472
		25-methyl-5α-ergost-24(28)-en-3β-ol[15]	60077056
		ergosta-5-24(28)-dien-3-β-ol[15]	6428671
		5α-stigmast-24-en-3β-ol[15]	21604800
		Stigmasterol[15]	5280794
		Patuletin[15]	5281678

		Palmitic acid[15]	985
		Stearic acid[15]	5281
		Arachidic acid[15]	10467
		Behenic acid[15]	8215
		Oxalic acid[15]	971
		Citric acid[15]	311
		Isocitric acid[15]	1198
		Oxaloacetate[15]	970
		Malic acid[15]	525
		Succinic acid[15]	1110
		Raffinose[15]	439242
		Lactose[15]	6134
		Sucrose[15]	5988
		Glucose[15]	5793
		Galactose[15]	6036
		Fructose[15]	2723872
		n-triacontane[15]	12535
		Hentriacontane[15]	12410
		(24S)-stigmast-25-enol[16]	129725970
		Bryophyllin C[16]	101049584
		Gallic acid[16]	370
		kaempferol-3-O-rutinoside[16]	5318767
		Kaempferitrin[16]	5486199

		Stigmast-24-enol[16]	129726020
		Afzelin[16]	5316673
		luteolin 7-O—glucoside[16]	5280637
		Bufalin[16]	9547215
		Quercitrin[17]	5280459
		α-rhamnoisorobin[17]	25079965
		1-Penten-3-one[18]	15394
		Hexanal[18]	6184
		1,3-Octadiene[18]	517653
		2-Hexenal[18]	5281168
		2-Heptanone[18]	8051
		Heptanal[18]	8130
		2,4-Hexadienal (E,E)[18]	637564
		1-Octen-3-ol[18]	18827
		Octanal[18]	454
		2-Octenal (E)[18]	5283324
		2-Octen-1-ol (E)[18]	5318599
		Nonanal[18]	31289
		2-Nonenal (E)[18]	5283335
		Decanal[18]	8175
		Dodecane[18]	8182
		Tridecane[18]	12388
		Tetradecane[18]	12389

		Pentadecane[18]	12391
		Butylated Hydroxytoluene[18]	31404
		Phenol, 2,4-bis (1,1-dimethylethyl)[18]	93344
		Pentanoic acid[18]	7991
		Hexadecane[18]	11006
		Butyrolactone[19]	7302
		3, 4 –Epoxytetrahydrothiophene-1,1-dioxide[19]	20597
		3,5-Dihydroxy-6-methyl-2,3-dihydro-4H-pyran-4-one[19]	119838
		Benzaldehyde[19]	240
		Alpha-D-Glucopyranoside, methyl[19]	64947
		Oleic acid[19]	445639
05	*Buddleja officinalis* Maxim.	Crocusatin C[20]	11105753
		Acacetin[20]	5280442
		Lariciresinol[20]	332427
		Pinoresinol[20]	73399
		Syringaresinol[20]	100067
		Mimengoside B[20]	54586748
		Songarosaponin A[20]	197452
		Clerodendrin[21]	5488004
		Quercetin 7-O-glucoside[21]	5282160
		Luteolin 7-O-rutinoside[21]	14032966
		Luteolin 7-O-glucoside[21]	5280637

		Luteolin 7-O-glucuronide[21]	5280601
		Isorhoifolin[21]	9851181
		Diosmetin 7-O-rutinoside[21]	5281613
		Apigenin 7-O-glucoside[21]	5280704
		Apigenin 7-O-glucuronide[21]	5319484
		Neobudofficide[21]	44257901
		Apigenin 7-(6”-malonylglucoside)[21]	5281602
		Linarin[21]	5317025
		Acacetin 7-O-glucoside[21]	5321954
		Luteolin[21]	5280445
		Acacetin 7-O-glucuronide[21]	5488681
		Apigenin[21]	5280443
		Salidroside[21]	159278
		Cistanoside F[21]	101688189
		Hebitol II[21]	53359965
		Syringin[21]	5316860
		3-Caffeoylquinic acid[21]	1794427
		Echinacoside[21]	5281771
		Isocampneoside II[21]	102277956
		Campneoside II[21]	102000758
		Hebeoside[21]	101390415
		Forsythoside A[21]	5281773
		Acteoside[21]	5281800

		Isoacteoside[21]	6476333
		Globusintenoside[21]	102072501
		Crocin I[21]	5281233
		Crocin II[21]	9940690
		Crocin III[21]	10461942
		Mimengoside C[22]	44566639
		Mimengoside D[22]	21602030
		Mimengoside E[22]	44566640
		Mimengoside F[22]	21602032
		Mimengoside G[22]	44566641
06	*Bupleurum hamiltonii* Balakr.	Camphene[23]	6616
		Alpha-Pinene[23]	6654
		Sabinene[23]	18818
		Beta-Pinene[23]	14896
		Beta-Myrcene[23]	31253
		p-Cymene[23]	7463
		Limonene[23]	22311
		Trans-Ocimene[23]	5281553
		Gamma-Terpinene[23]	7461
		Linalool[23]	6549
		Bornyl acetate[23]	6448
		Beta-Elemene[23]	6918391
		Alpha-Copaene[23]	19725

		Trans-Caryophyllene[23]	5281515
		Alpha-Humulene[23]	5281520
		Trans--Farnesene[23]	5281517
		Germacrene D[23]	5317570
		Alpha-Selinene[23]	10856614
		(E,E)-alpha-farnesene[23]	5281516
		Gamma-Cadinene[23]	92313
		Delta-Cadinene[23]	441005
		(E)-nerolidol[23]	5284507
		Spathulenol[23]	92231
		Caryophyllene oxide[23]	1742210
		Iso-Longifolol[23]	91746504
		(Z)-lanceol[23]	15560069
07	*Bupleurum marginatum* Wall.ex DC.	Hexanal[24]	6184
		Heptanal[24]	8130
		Nonane[24]	8141
		Alpha-Thujene[24]	17868
		Heptanol[24]	8129
		6-Methyl-5-heptene-2-one[24]	9862
		Octan-2-one[24]	8093
		Pentylfuran[24]	19602
		Octanal[24]	454
		Limonene[24]	22311

		Cis-Linalool oxide[24]	6428573
		P-Cymenene[24]	62385
		Beta-Linalool[24]	6549
		n-Undecane[24]	14257
		Trans-Pinocarveol[24]	88302
		DL-Menthone[24]	6986
		(E)-2-Nonenal[24]	5283335
		Borneol[24]	64685
		Alpha-Terpineol[24]	17100
		Myrtenol[24]	10582
		b-Cyclocitral[24]	9895
		Cis-Carveol[24]	330573
		Piperitone[24]	6987
		(E)-Anethole[24]	637563
		Thymol[24]	6989
		3-Undecanol[24]	98970
		2-Undecanol[24]	15448
		n-Tridecane[24]	12388
		Alpha-Cubebene[24]	442359
		(+)Longicyclene[24]	71311545
		Alpha-Copaene[24]	70678558
		Alpha-Bourbonene[24]	530816
		Iso-Longifolene[24]	11127402

		Beta-Elemene[24]	6918391
		Beta-Caryophyllene[24]	5281515
		Beta-Gurjunene[24]	6432176
		Geranyl acetone[24]	1549778
		Alpha-Humulene[24]	5281520
		Beta-Farnesene[24]	5281517
		Germacrene D[24]	5317570
		Beta-Selinene[24]	442393
		Alpha-Muurolene[24]	12306047
		Alpha-Farnesene[24]	5281516
		n-Pentadecane[24]	12391
		Delta-Cadinene[24]	441005
		Alpha-Calacorene[24]	12302243
		Spathulenol[24]	92231
		Beta-Caryophyllene oxide[24]	1742210
		Davanone[24]	519782
		Viridiflorol[24]	11996452
		Salvial-4(14)-en-1-one[24]	42608172
		Cubenol[24]	519857
		t-Cadinol[24]	160799
		Delta-Cadinol[24]	3084311
		Cis-alpha-Santalol[24]	5281531
		(Z,E)-Farnesol[24]	1549108

		Alpha-Cyperone[24]	6452086
		n-Heptadecane[24]	12398
		Hexadecanal[24]	984
		Hexahydrofarnesyl acetone[24]	10408
		Neophytadiene[24]	10446
		Hexadecanol[24]	2682
		Palmitic acid[24]	985
		Phytol[24]	5280435
		Chinensin[25]	5315827
		(–)-Matairesinol[25]	119205
		Octacosanoic acid[25]	10470
		Diphyllin[25]	100492
		Kaerophyllin[25]	6440534
		Alpha-peltatin[25]	92129
		Butyrolactone[25]	7302
		Demethylyatein[25]	31651
		Suchilactone[25]	132350840
		Vanillic acid 4-β-D-glucoside[25]	14132336
		Kaempferol 3-O-neohesperidoside[25]	5318761
		Citroside A[25]	14312562
		Dehydrodiconiferyl alcohol 4-O-β-D-glucopyranoside[25]	5316442
		Styraxlignolide C[25]	11398272
		Clinoposaponin XI[25]	190938

		Quercetrin[26]	5280459
		Isorhamnetin[26]	5281654
		Narcissin[26]	5481663
		Alpha-spinasterol[26]	5281331
		Rutin[26]	5280805
		Stigmasterol[26]	5280794
		Beta-sitosterol[26]	222284
		Daucosterol[26]	5742590
		Alpha-spinasterol glucoside[26]	12960498
		Saikosaponin A[27]	167928
		Saikosaponin D[27]	107793
		Hydroxysaikosaponin a[27]	101690817
		Saikosaponin b1[27]	9875547
		Saikosaponin f[27]	21598300
		Saikosaponin b2[27]	21637642
		Saikosaponin b4[27]	21637636
		Saikochromoside A[27]	70697379
		Rotundifolioside A[27]	11061975
		Malonylsaikosaponin d[27]	102059427
		Saikosaponin n[27]	100962153
		Malonylsaikosaponin a[27]	102059426
		Saikosaponin b3[27]	21637635
		1, 2, 3, 7-Tetramethoxyxanthone[27]	14528828

		Rotundioside F[27]	101093904
		Rotundifolioside J[27]	101426597
		Rotundifolioside I[27]	10865866
		(3,4-dimethoxybenzyl)-2-(3,4-methylenedioxybenzyl) butyrolactone[28]	129848926
		Myricetin[29]	5281672
08	*Buxus bodinieri* Lévl.	Buxbodine B[30]	91895280
		Buxbodine D[30]	91895277
09	*Caesalpinia sappan* Linn.	Caesalpin J[31]	127260
		(E)-7-hydroxy-3-(4-hydroxybenzylidene)chroman-4-one[31]	44443280
		Sappanone B[31]	13888976
		3’-deoxy-4-O-methylsappanol[31]	13846680
		Caesalpiniaphenol F[31]	14522836
		3’-deoxyepisappanol[31]	23259347
		3’-deoxy-4-O-methylepisappanol[31]	71463283
		4,4 -dihydroxy-2 -methoxychalcone[31]	5319688
		Sappanchalcone[31]	5319493
		Protosappanin A[31]	128001
		(-)-Protosappanin B[31]	13846689
		10-O-Methylprotosappanin B[31]	5319768
		Brazilin[31]	73384
		(-)-Balanophonin[31]	23252258

		(-)-syringaresinol[31]	11604108
		(-)-episyringaresinol[31]	45482321
		Coniferyl aldehyde[31]	5280536
		4–hydroxy-3-methoxybenzaldehyde[31]	604791
		2,4-dihydroxybenzoic acid[31]	1491
		(-)-3-deoxysappanone B[31]	57391100
		Brazelein[32]	6453902
		Brazilide A[32]	102501911
		2,4,5-Trihydroxybenzaldehyde[32]	643387
		Euxanthone[32]	5281631
		3,8,9-Trihydroxy-6H-benzo[c] chromen-6-one[32]	60198001
		3-Deoxysappanone B[32]	15703606
		Butein[32]	5281222
		Protosappanin C[32]	13846692
		Protosappanin D[32]	101405825
		Phanginin I[33]	24824767
		Phaginin A[33]	101844809
		Phanginin D[33]	101844812
		Phanginin H[33]	24854208
		Phanginin J[33]	24854209
		Caesalpiniaphenol G[34]	102499318
		Quercetin[34]	5280343

		5-hydroxy-1,4-naphthoquinone[35]	3806
		5-hydroxy-2-methyl-1,4-naphthoquinone[35]	10205
		1,4-naphthoquinone[35]	8530
		1,2-naphthoquinone[35]	10667
		Methoxychalcone[35]	5367166
		7,10-Dihydroxy-11-methoxydracaenone[36]	3081036
		1,5-Dihydroxyxanthone[36]	5480299
		3-Allyl-6-methoxyphenol[37]	596375
		4-Hydroxy-3,5-dimethoxybenzaldehyde[37]	8655
		4-Hydroxy-3-methoxybenzaldehyde[37]	1183
		Squalene[37]	638072
		Friedelan-3-one[37]	91472
		Tetradecanoic acid[37]	11005
		n-Hexadecanoic acid[37]	985
		(9Z,12Z)-Octadeca-9,12-dienoic acid[37]	5280450
		p-Methoxycinnamic acid ethyl ester[37]	5281783
		Benzyl benzoate[37]	2345
		Campesterol[37]	173183
		Stigmasta-5,22-dien-3-ol[37]	53870683
		22,23-Dihydrostigmasterol[37]	222284

		(+)-lyoniresinol[38]	11711453
		3'-O-methylbrazilin[38]	13846641
		Caesalpiniaphenol A[39]	71454364
		Caesalpiniaphenol B[39]	71457914
		Caesalpiniaphenol C[39]	71452598
		Caesalpiniaphenol D[39]	71457913
		Quercetin-3,7-di-O-methyl ether[39]	5280417
		10,11-dihydroxydracaenone C[39]	71450773
		Beta-amyrin[40]	73145
		2-deoxyribose[40]	5460005
		Sappanol[40]	13846649
		3’-deoxysappanol[40]	13846660
		4,4’-dihydroxy-2’-methoxychalcone[40]	6442675
		Ombuin[40]	5320287
		4,4’-dihydroxy-2’-methoxychalcone[40]	129864633
		8-methoxybonducellin[40]	73299135
		Neosappanone A[40]	101353537
		Capric acid[40]	2969
		Lauric acid[40]	3893
		Palmitoleic acid[40]	445638
		Arachidic acid[40]	10467
		Tetraacetylbrazilin[40]	192761

		Hematoxylin[40]	442514
10	*Calliandra haematocephala* Hassk.	(epi)catechin[41]	72276
		(epi)afzelechin[41]	443639
		Gallocatechin[41]	65084
		Catechin[41]	9064
		Epigallocatechin-3-O-gallate[41]	65064
		Catechin benzylthioether[41]	101607233
		Epicatechin benzylthioether[41]	101607232
		Myricetin[42]	5281672
		Quercetin[42]	5280343
		Pipecolic acid[43]	849
		Cis-5-hydroxypipecolic acid[43]	11008043
		p-hydroxybenzoic acid[44]	135
		Protocatechuic acid[44]	72
		Caffeic acid[44]	689043
		Astilbin[44]	119258
		Betulinic acid[44]	64971
		Niclosamide[44]	4477
		Oxyclozanide[44]	16779
		Bithionol[44]	2406
		Cholestanol[45]	6665
		Campesterol[45]	173183
		Stigmasterol[45]	5280794

		Stigmastanol[45]	241572
		β- Sitosterol[45]	222284
		Lauric acid[45]	3893
		Myristic acid[45]	11005
		Palmitic acid[45]	985
		Stearic acid[45]	5281
		Oleic acid[45]	445639
		Linoleic acid[45]	5280450
		Linolenic acid[45]	5280934
11	*Calystegia hederacea* Wall. ex Roxb.	Tiglic acid[46]	125468
		11S-hydroxyhexadecanoic acid[46]	12575964
		p-bromophenacyl 2-methylbutyrate[47]	14033789
		p-bromophenacyl tiglate[47]	25231770
		p-bromophenacyl nilate[47]	14033791
		Methyl 11-hydroxyhexadecanoate[47]	602938
		Methyl 12-hydroxyhexadecanoate[47]	15569763
		Calysolin II[47]	56833467
		Calysolin III[47]	56833554
12	*Camellia sinensis* (L.) O. Ktze.	Epigallocatechin gallate[48]	65064
		Epicatechin 3-gallate[48]	65056
		Epigallocatechin[48]	72277
		Epicatechin[48]	72276

		Catechin[48]	9064
		(−)-5-(3′,4′, 5′-trihydroxyphenyl)-gamma valerolactone[48]	44389277
		(−)-5-(3′, 4′-dihydroxyphenyl)-gamma valerolactone[48]	152432
		Caffeine[48]	2519
		Theobromine[49]	5429
		Theaflavin[49]	135403798
		L-theanine[49]	439378
		Myricetin[49]	5281672
		Theaflavin3-gallate[49]	136825044
		Theaflavin-3′-gallate[49]	136825043
		Theaflavin-3,3′-digallate[49]	135403795
		Theaflavate C[49]	101446897
		Bistheaflavate A[49]	101446898
		Proepitheaflagallin[49]	101438247
		Theasinensin A[49]	442543
		Theasinensin B[49]	467315
		Theasinensin C[49]	467317
		Theasinensin F[49]	467316
		Quercetin-3-rutinoside[49]	5280805
		Gallic acid[49]	370
		Quinic acid[49]	6508
		Caffeoylquinic acid[49]	1794427

		Xanthosine[49]	64959
		7-methylxanthosine[49]	23724732
		Fructose[49]	2723872
		Glucose[49]	5793
		Sucrose[49]	5988
		Rhamnose[49]	25310
		Galactose[49]	6036
		Lactose[49]	6134
		Arabinose[49]	439195
		Xylose[49]	135191
		Mannose[49]	18950
		Ribose[49]	10975657
		Galacturonic acid[49]	439215
		Glucuronic acid[49]	94715
		Linalool[49]	6549
		(Z)-hex-3-enal[49]	643941
		(Z)-1,5-octadien 3-one[49]	6429343
		4-mercapto-4-methyl-2-pentanone[49]	88290
		Methional[49]	18635
		3-methylnonane-2,4-dione[49]	529481
		2-acetyl-1-pyrroline[49]	522834
		2-ethyl-3,5-dimethylpyrazine[49]	26334
		2,3- diethyl-5-methylpyrazine[49]	28905

		2-acetyl-2-thiazoline[49]	169110
		β-damascone[49]	5374527
		β-damascenone[49]	5366074
		(Z)- methyl jasmonate[49]	6430765
		Phenylmethyl acetate[49]	8785
		Phenylethyl acetate[49]	7654
		2-methylpropyl benzoate[49]	61048
		Puerin A[49]	101377924
		Puerin B[49]	101377925
		Teadenol A[49]	68196392
		Teadenol B[49]	68196394
		Fuzhuanin A[49]	71813433
		Fuzhuanin B[49]	71813434
		Fuzhuanin C[49]	101879768
		Fuzhuanin D[49]	101879769
		Fuzhuanin E[49]	101879770
		Fuzhuanin F[49]	101879771
		Planchol A[49]	11543679
		Xanthocerin[49]	134771583
		Beta-glucogallin[50]	124021
		Galloylquinic acid[50]	129650210
		Quercetin 3-O-glucoside[50]	5280804
		Myricetin 3-O-glucoside[50]	22841567

		Gallocatechin[50]	65084
		1,2,6-Trigalloylglucose[50]	440308
		Myricetin 3-O-galactoside[50]	5491408
		Quercetin 3-O-glucosylrutinoside[50]	102332276
		Kaempferol 3-O-galactoside[50]	5462193
		Kaempferol-3,7-di-O-α-L-rhamnoside[51]	5486199
		Quercetin-3,7-di-O-α-L-rhamnopyranoside[51]	15953752
		Myricetin -3-O-α-L-rhamnopyranoside[51]	5352000
		Strictinin[51]	73330
		Pinoresinol 4′-O-β-D-glucopyranoside[51]	486614
		Methyl gallate[51]	7428
		Catechol[51]	289
		Pyrogallol[51]	1057
		4,4′-methylenebis[1,2,3-benzenetriol][51]	21896293
		p-hydroxyphenethyl alcohol[51]	10393
		3,4-dihydroxybenzoic acid[51]	72
		2-(4-hydroxyphenyl)ethyl β-D-glucopyranoside[51]	159278
		Coniferin[51]	5280372
		Theogallin[51]	442988
		Theophylline[52]	2153

		Linoleic acid[52]	5280450
		Linolenic acid[52]	5280934
		Catechin gallate[52]	6419835
13	*Cardiocrinum giganteum* (Wall.) Makino	Quercetin[53]	5280343
		Apigenin[53]	5280443
		Kaempferol[53]	5280863
14	*Carex baccans* Nees	(+)-alpha-viniferin[54]	196402

References

1. Xu, D., Pan, Y. & Chen, J. Chemical constituents, pharmacologic properties, and clinical applications of bletilla striata. *Front. Pharmacol.* **10**, 1–19 (2019).

2. Hu, M. *et al.* Chemical Composition of Tubers of Bletilla striata. *Chem. Nat. Compd.* **55**, 555–556 (2019).

3. Zhao, Y. *et al.* Chemical constituents from Bletilla striata and their NO production suppression in RAW 264.7 macrophage cells. *J. Asian Nat. Prod. Res.* **20**, 385–390 (2018).

4. Woo, K. W., Park, J. E., Choi, S. U., Kim, K. H. & Lee, K. R. Phytochemical constituents of bletilla striata and their cytotoxic activity. *Nat. Prod. Sci.* **20**, 91–94 (2014).

5. Wu, T. Y., Chen, C. C. & Lay, H. L. Study on the components and antioxidant activity of the Bletilla plant in Taiwan. *J. Food Drug Anal.* **18**, 279–289 (2010).

6. Nishidono, Y. *et al.* Effect of heat processing on the chemical constituents and NO-suppressing activity of Bletilla Tuber. *J. Nat. Med.* **74**, 219–228 (2020).

7. Verma, R. C. P. R. S., Chauhan, A. & Á, E. Á. B. Á. G. D. Compositional Variations in Volatile Constituents of Boenninghausenia albiflora Reichb . from Western Himalaya. **36**, 635–640 (2013).

8. Taylor, P., Padalia, R. C., Verma, R. S., Chauhan, A. & Chanotiya, C. S. Natural Product Research : Formerly Natural Product Letters Chemical composition of leaf and root essential oils of Boenninghausenia albiflora Reichb . from northern India. 37–41.

9. Journals, I. Essential Oil Constituents of Boenninghausenia albiflora Reichb . (Rutaceae) from Gangolihat , (Distt-Pithoragarh). (2018).

10. Joshi, B. *et al.* Medicinal Plants of Nepal Selected Based on Ethnobotanical Evidence. **2020**, (2020).

11. Joshi, K. R., Devkota, H. P. & Yahara, S. NPC Natural Product Communications. **8**, 7–8 (2013).

12. Karole, S., Gautam, G. & Gupta, S. PROFILE OF BOMBAX CEIBA. **6**, (2017).

13. Refaat, J. *et al. Bombacaceae : A phytochemical review Bombacaceae : A phytochemical review*. vol. 0209 (2013).

14. Ot, O. Chemical Composition and Antioxidant Activity of Bryophyllum pinnatum Root. *Nat. Prod. Chem. Res.* **3**, (2015).

15. Kamboj, A. & Saluja, A. K. PHCOG REV .: Review Article Bryophyllum pinnatum (Lam .) Kurz .: Phytochemical and Pharmacological Profile : A Review. 364–374 (2009).

16. Fernandes, J. M., Cunha, L. M., Azevedo, E. P., Fernandes-pedrosa, M. F. & Zucolotto, S. M. Kalanchoe laciniata and Bryophyllum pinnatum : an updated review about ethnopharmacology , phytochemistry , pharmacology and toxicology. **29**, 529–558 (2019).

17. Latif, A. *et al.* PHYTOCHEMICAL AND PHARMACOLOGICAL PROFILE OF THE MEDICINAL HERB : BRYOPHYLLUM PINNATUM. **29**, (2019).

18. Zawirska-wojtasiak, R., Jankowska, B., Piechowska, P. & Mildner-, S. Vitamin C and aroma composition of fresh leaves from Kalanchoe pinnata and Kalanchoe daigremontiana. *Sci. Rep.* 1–8 (2019) doi:10.1038/s41598-019-56359-1.

19. Uchegbu, R. I., Ahuchaogu, A. A., Amanze, K. O. & Ibe, C. O. Chemical Constituents Analysis of the Leaves of Bryophyllum pinnatum by GC-MS. **3**, 19–22 (2017).

20. Park, T. W. *et al.* Chemical constituents from buddleja officinalis and their inhibitory effects on nitric oxide production. *Nat. Prod. Sci.* **22**, 129–133 (2016).

21. Xie, G. *et al.* Chemical profiles and quality evaluation of Buddleja officinalis flowers by HPLC-DAD and HPLC-Q-TOF-MS/MS. *J. Pharm. Biomed. Anal.* **164**, 283–295 (2019).

22. Guo, H. *et al.* Saponins from the Flower Buds of Buddleja officinalis. *J. Nat. Prod.* **67**, 10–13 (2004).

23. Pande, C., Tewari, G., Singh, C. & Singh, S. Chemical composition of the essential oil from the flowering aerial parts of Bupleurum hamiltonii Balak from Uttarakhand, India. *Nat. Prod. Res.* **26**, 1442–1445 (2012).

24. Ashour, M. L. *et al.* Chemical composition and biological activity of the essential oil obtained from <I>Bupleurum marginatum</I> (Apiaceae). *J. Pharm. Pharmacol.* **61**, 1079–1087 (2009).

25. Liu, X. *et al.* Bupleurum marginatum Wall . ex DC in Liver Fibrosis : Pharmacological Evaluation , Differential Proteomics , and Network Pharmacology. **9**, 1–14 (2018).

26. Ashour, M. L., Youssef, F. S., Gad, H. A. & El-readi, M. Z. Evidence for the anti-inflammatory activity of Bupleurum marginatum (Apiaceae) extracts using in vitro and in vivo experiments supported by virtual screening. (2018) doi:10.1111/jphp.12904.

27. Liang, Z., Zhang, J., Yang, G., Chen, H. & Zhao, Z. Chemical profiling and histochemical analysis of Bupleurum marginatum roots from different growing areas of Hubei province. *Acta Pharm. Sin. B* **3**, 193–204 (2013).

28. Ashour, M. L., El-Readi, M. Z., Tahrani, A., Eid, S. Y. & Wink, M. A novel cytotoxic aryltetraline

lactone from Bupleurum marginatum (Apiaceae). *Phytochem. Lett.* **5**, 387–392 (2012).

29. Lei, Z. *et al.* A new triterpenoid and a new flavonoid glycoside isolated from Bupleurum marginatum and their anti-inflammatory activity. *Nat. Prod. Res.* **0**, 1–7 (2019).

30. Zhang, J. *et al.* Chemical Constituents of Plants from the Genus Buxus. *Chem. Biodivers.* **12**, 1289–1306 (2015).

31. Ji, Y. *et al.* Chemical constituents from heartwoods of Caesalpinia sappan with antiplatelet aggregation activities. **11**, 423–428 (2019).

32. Nirmal, N. P., Rajput, M. S., Prasad, R. G. S. V & Ahmad, M. Asian Paci fi c Journal of Tropical Medicine Brazilin from Caesalpinia sappan heartwood and its pharmacological activities : A review. *Asian Pac. J. Trop. Med.* **8**, 421–430 (2015).

33. Tran, M. H. *et al.* Cytotoxic constituents from the seeds of Vietnamese Caesalpinia sappan. *Pharm. Biol.* **00**, 1–6 (2015).

34. Nguyen, V. B. *et al.* Phenolic Compounds from Caesalpinia sappan. **12**, 410–414 (2020).

35. Zanin, J. L. B. *et al.* The Genus Caesalpinia L. (Caesalpiniaceae): Phytochemical and Pharmacological Characteristics. 7887–7902 (2012) doi:10.3390/molecules17077887.

36. Zhao, M. *et al.* Two New Phenolic Compounds from the Heartwood of Caesalpinia sappan L. 1–8 (2014) doi:10.3390/molecules19010001.

37. Linn, S. & Saiai, A. PHYTOCHEMICAL AND CYTOTOXIC INVESTIGATIONS OF THE HEARTWOOD OF CAESALPINIA. **11**, 11–14 (2018).

38. Fu, L. *et al.* A New 3-Benzylchroman Derivative from Sappan Lignum (Caesalpinia sappan). 1923–1930 (2008) doi:10.3390/molecules13081923.

39. Cuong, T. D. *et al.* Phenolic Compounds from Caesalpinia sappan Heartwood and Their Anti-in fl ammatory Activity. 10–16 (2012).

40. Pawar, C. R., Landge, A. D. & Surana, S. J. Phytochemical and Pharmacological Aspects of Caesalpinia sappan. **1**, 131–138 (2008).

41. Taylor, P., Wei, S., Chen, H. & Lin, Y. Comparison of Chemical Compositions and Antioxidant Activities of Condensed Tannins From Different Parts of Calliandra haematocephala. 37–41 doi:10.1080/02773813.2014.919596.

42. Punnagai, K. & I, G. J. ALPHA-AMYLASE AND ALPHA-GLUCOSIDASE INHIBITORY EFFECTS OF CALLIANDRA HAEMATOCEPHALA AND ITS POTENTIAL ROLE IN DIABETES MELLITUS. **11**, (2018).

43. Brenner, S. A. & Romeo, J. T. Fungitoxic Effects of Nonprotein Imino Acids on Growth of Saprophytic Fungi Isolated from the Leaf Surface of Calliandra haematocephala. **51**, 690–693 (1986).

44. Tiwari, J. & Shukla, A. Investigations on Calliandra haematocephala flowers extract for in-vitro anthelmintic activity. **1**, 17–20 (2016).

45. Abo-elhamd, A. M. *et al.* Journal of Chemical and Pharmaceutical Research , 2016 , 8 (4): 828-845 Research Article. **8**, 828–845 (2016).

46. Ono, M., Saito, N., Minamishima, H., Yasuda, S. & Nohara, T. Two new glycosidic acids , calyhedic acids E and F , in crude resin glycoside fraction from Calystegia hederacea. *Nat. Prod. Res.* **0**, 1–8 (2020).

47. Ono, M. *et al.* Identification and characterization of organic and glycosidic acids in crude resin glycoside fraction from Calystegia hederacea. *J. Nat. Med.* (2019) doi:10.1007/s11418-019-01366-9.

48. Reto, M., Figueira, M. E., Filipe, H. M. & Almeida, C. M. M. Chemical composition of green tea (Camellia sinensis) infusions commercialized in Portugal. *Plant Foods Hum. Nutr.* **62**, 139–144 (2007).

49. Zhang, L. *et al.* Chemistry and Biological Activities of Processed Camellia sinensis Teas: A Comprehensive Review. *Compr. Rev. Food Sci. Food Saf.* **18**, 1474–1495 (2019).

50. Wang, Y. S. *et al.* Influence of shade on flavonoid biosynthesis in tea (Camellia sinensis (L.) O. Kuntze). *Sci. Hortic. (Amsterdam).* **141**, 7–16 (2012).

51. Meng, X. H. *et al.* C-8 N-Ethyl-2-pyrrolidinone-Substituted Flavan-3-ols from the Leaves of Camellia sinensis var. pubilimba. *J. Agric. Food Chem.* **66**, 7150–7155 (2018).

52. Koch, W., Zagórska, J., Marzec, Z. & Kukula-Koch, W. Applications of tea (Camellia sinensis) and its active constituents in cosmetics. *Molecules* **24**, 1–28 (2019).

53. Xia, X. *et al.* Isolation and Identification of Antioxidant Flavonoids from the Seeds of Cardiocrinum Giganteum var . Yunnanense. *Pharm Biomed Sci* **06**, 374–377 (2016).

54. Kumar, D., Gupta, N., Ghosh, R., Gaonkar, R. H. & Pal, B. C. a -Glucosidase and a -amylase inhibitory constituent of Carex baccans : Bio-assay guided isolation and quantification by validated RP-HPLC – DAD. *J. Funct. Foods* **5**, 211–218 (2012).
